# Treating cognitive impairments in primary central nervous system infections: A systematic review of pharmacological interventions

**DOI:** 10.1097/MD.0000000000034151

**Published:** 2023-07-14

**Authors:** Syeda T. Rizvi, Jhankhana S. Shah, Sarah Shaaya, Tatyana Mollayeva

**Affiliations:** a University of Toronto, Toronto, ON; b KITE Research Institute University Health Network, Toronto, ON; c Trinity College Institute of Neuroscience, Trinity College Dublin, Dublin 2, Ireland.

**Keywords:** antimicrobials, clinical trial, cognitive recovery, drug therapy, infection, rehabilitation

## Abstract

This research synthesized scientific evidence on the use of pharmacotherapy as intervention to reduce cognitive impairments in adult patients with primary central nervous system (CNS) infections.

**Methods:**

We searched for experimental studies published in English prior to October 2021 in MEDLINE, Embase and Cochrane databases. We included non-randomized studies (NRS) and randomized control trials (RCT) of pharmacotherapy versus placebo, drug, or a combination of drugs in adults with primary CNS infection. The certainty of the evidence was rated according to GRADE guidelines.

**Results:**

We included 8 RCTs and 1 NRS, involving a total of 805 patients (50.77% male patients; mean age 42.67 ± 10.58) with Lyme disease (LD), herpes simplex virus type 1 (HSV-1), or Creutzfeldt–Jakob disease (CJD) studying the efficacy of antibiotics, antiviral, and non-opioid analgesic drugs, respectively. In patients with LD, antibiotics alone or in combination with other drugs enhanced certain cognitive domains relative to placebo. In patients with HSV-1, the results were inconsistent. In patients with CJD, flupirtine maleate enhanced baseline cognitive scores. The quality of RCT studies was low, and the quality of NRS of intervention was very low, suggesting low and very low certainty in the reported results.

**Conclusion:**

There is limited evidence and low certainty regarding the efficacy of antimicrobials and analgesics in reducing cognitive impairments in patients with LD, HSV-1, and CJD. Future efforts must be aimed at enhancing attention to clinical trial methodology and reporting, as well as reaching a consensus on outcome measures and the endpoint of clinical trials relevant to patients.

## 1. Introduction

Infections of the central nervous system (CNS) are a heterogeneous group of disorders with respect to etiology and the effect on the nervous system. An infection refers to “the invasion of an organism’s body tissues by disease-causing agents, their multiplication, and the reaction of host tissues to the infectious agents and the toxins they produce.”^[1]^ Many infectious agents (e.g., viruses, bacteria, fungi, prions, protozoa, and helminths)^[2]^ may impose damage via direct invasion of the nervous system and early activation of the immune system with subsequent brain tissue damage, causing primary CNS infections,^[3]^ and leaving the patient with cognitive impairments that may or may not follow a progressive neurological course.

Several longitudinal studies have reported a relationship between CNS infections during mid-life and cognitive impairment later in life,^[4]^ when the patient is no longer affected by the infection, indicating the long-term effects infection can have on cognition.^[4,5]^ Long-term cognitive impairments within one or several domains of cognitive function such as memory, orientation, comprehension, learning, and language due to CNS infections have been noted to affect 20% to 40% of patients.^[5]^ Research has shown that patients with CNS infections are also at increased risk of developing deficits in auditory differentiation and fine motor function.^[6,7]^ The pathological processes through which CNS infections may influence cognitive function are generally divided into early functional (i.e., relating to regulation and maintenance of cerebral blood flow) and structural changes in the brain (i.e., increased white matter lesions, cerebral atrophy) in response to inflammation.^[3,5]^

The “seeding” hypothesis^[8]^ suggests that infectious agents invade the CNS and stimulate the microglia to induce an immune reaction that changes the levels of enzymes (i.e., neprilysin and insulin-degrading enzymes via inflammatory cytokines) that trigger production of amyloid proteins. Failure to clear amyloid proteins raises inflammation and expedites amyloid accumulation, which is a mechanism recognized in dementia.^[8]^ These processes can be attenuated via drug interventions targeting infectious agents and/or the inflammation they cause.^[9]^ For example, antibiotics (i.e., antibacterial, antiviral, and antiparasitic) either inhibit growth or kill infectious agents that cause CNS infection, invade neurons and cause damage.^[10,11]^ Polyphenols have immunomodulatory and anti-inflammatory properties.^[12,13]^ Many other medications with anti-inflammatory or neuroprotective properties^[14,15]^ may also be beneficial in protecting neurons by slowing down or stopping microglial activation and, consequently, preventing or reversing cognitive deterioration. Hence, an evaluation of the role of pharmacotherapy in diminishing cognitive impairments stemming from primary CNS infection is warranted. We conducted a systematic review on the use of pharmacotherapy as intervention to reduce cognitive impairments in adult patients with primary CNS infections, with objectives to: provide a summary of the evidence on pharmacological interventions used in the treatment of cognitive impairments; evaluate whether these interventions improve cognitive outcomes; and report on specific intervention characteristics that may have an impact on results of interventions.

## 2. Methods

This systematic review was conducted and reported according to the Preferred Reporting Items for Systematic Reviews and Meta-Analyses guidelines.

### 2.1. Eligibility criteria

Peer-reviewed intervention studies published in English, conducted in adult patients (≥18 years old) with primary CNS infections that measured cognition through clinical diagnosis or standardized measurement tools, regardless of the setting, were considered eligible. Case reports, pediatric studies, dissertations, and articles with no primary data were excluded. Eligible studies were selected according to the inclusion criteria by the PICOS strategy:

- *Participants (P):* adult participants aged 18 years and above with primary CNS infection in any geographic region worldwide for whom intervention was prescribed to support cognitive recovery.
- *Interventions (I):* any pharmacological intervention, single or multicomponent, aiming to improve cognitive outcome.
- *Comparisons (C):* standard care (i.e., not providing any intervention specifically aimed at dealing with cognitive decline, placebo, or both) or comparisons between interventions.
- *Outcome measures (O):* change in the level of cognitive impairment in any of cognitive domains and sub-domains assessed by standardized measures of cognitive function.
- *Study design (S):* experimental studies, i.e., randomized controlled trials (RCT) and nonrandomized studies (NRS) of interventions were considered.

### 2.2. Information sources and search strategy

Researchers in collaboration with the information specialist at the large rehabilitation research teaching hospital developed a subject-specific MEDLINE search strategy and modified it several times to retrieve all relevant studies. The MEDLINE search strategy was adapted for searching all other databases, including MEDLINE(R) ALL (in Ovid, including Epub Ahead of Print, In-Process & Other Non-Indexed Citations, Ovid MEDLINE(R) Daily), Embase (Ovid), and the Cochrane CENTRAL Register of Controlled Trials (Ovid), and covered searches period from inception to November 2019. Repeated searches were performed in October 2021 to identify new publications (Table S1, Supplemental Digital Content, http://links.lww.com/MD/J199), which illustrates the full search strategies used for all databases).

### 2.3. Data collection and analysis

#### 2.3.1. Selection process.

Four review authors (J.S., S.D., S.S., S.R.) independently screened identified titles and abstracts in the searches for eligibility. Potentially relevant studies were retrieved for full-text review. Researchers were not blind to authors, institutions, or the publication sources of funding. A senior review author (T.M.) monitored the quality of the process and acted as quality assurer and arbiter if variances concerning study eligibility were noted between the reviewers. Disagreements were resolved through group discussion.

#### 2.3.2. Data collection process and data items process.

Two independent reviewers (J.S. and S.S.) independently conducted a full-text review of relevant articles, extracted, and collected data using a standardized data extraction sheet, developed by the senior review author (T.M.), which was piloted in the first 3 studies in the review, for study characteristics and outcome data. Data extracted from the studies that met the inclusion criteria included:

- *Study identifiers* (i.e., author names, publication year, setting, country).
- *Study characterizations* (type of infectious agent, objectives, groups, type of intervention, design, sample size, inclusion and exclusion criteria).
- *Methodological qualities* (i.e., study design, attrition rate, inclusion/exclusion criteria, sample size).
- *Participant characteristics* (i.e., mean age, sex, range, duration of illness, comparison at baseline).
- *Intervention characteristics* (i.e., dosage, frequency, mode of administration, duration of administration, change over time).
- *Outcomes*, tools used to measure cognitive function, cognitive domains assessed (i.e., complex attention; executive functioning; learning and memory; language; perceptual-motor ability; and social cognition,^[16]^ statistical methods and key findings (pre-intervention, post-intervention, change from pre to post and statistical results).

Two review authors (S.R. and J.S.) independently checked each study’s characteristics for accuracy of data extraction against the study report. A senior review author (T.M.) monitored the quality of the process and double-checked that data were entered correctly.

### 2.4. Assessment of risk of bias in included studies

#### 2.4.1. Study risk of bias assessment.

For data from the NRS intervention study,^[17]^ we used the Risk Of Bias in Non-randomized Studies of Interventions tool, version of 2016,^[18]^ assessing the following 7 domains: bias due to confounding, bias in the selection of patients into the study, bias in classification of interventions, bias due to deviations from the intended intervention, bias due to missing data, bias in the measurement of outcomes, and bias in the selection of the reported results. The risk of bias for RCT studies was assessed using version 2 of the Cochrane risk-of-bias tool for randomized trials (RoB 2). The RoB 2 considers the risk of bias in 5 domains which include the randomization process, deviations from the intended intervention, missing outcome data, measurement of the outcome, and selection of the reported results; each domain was judged as having “low,” “some concerns,” or “high” risk of bias based on an algorithm.^[19]^ The overall risk of bias judgment was made for each study – a study was considered “low” when all domains were designated “low” risk of bias; “some concerns” when at least 1 domain was designated as having “some concerns” but where no domains had a “high” risk of bias; and “high” when at least one domain was designated “high” risk of bias or when multiple domains had “some concerns” in a way that lowered the assessor’s confidence in the results.^[19]^ Each study was independently assessed by 2 researchers (J.S. and S.R., with T.M. overseeing the process), who then compared their final assessments. In the case of a disagreement, the study in question was independently assessed by the senior author (T.M.) and all group members had a discussion to reach a consensus.

#### 2.4.2. Effect measures.

The continuous outcome of change in cognitive function from pre- to post-intervention was presented as mean difference, group-time mean difference, or difference in change, as the data was provided in each study. The results were stratified by type of infectious agent, type and dose of drug, duration of drug administration (where possible), and cognitive domain and sub-domains assessed. When analyzing RCT studies, our interpretations were based on the principle of intention to treat (i.e., we analyzed the results of patients in their randomized group). A threshold of *P* < .05 designated statistical significance.

### 2.5. Dealing with missing data

We contacted study investigators to verify key study characteristics, aiming to obtain missing data where possible. Because of no response, we considered the impact of missing data in studies on the overall risk of bias assessed.

### 2.6. Synthesis methods

The Cochrane recommendations for conducting meta-analyses were used to determine if a meta-analysis was possible based on the assessment of clinical heterogeneity.^[20]^ Study characteristics were tabulated into a file to evaluate similarities between studies (Table S2, Supplemental Digital Content, http://links.lww.com/MD/J200). The heterogeneity observed at all levels across PICOS criteria (i.e., population, intervention type, dose and duration, comparison, outcome measures, and study design), precluded a meta-analysis in its classical form. We carried out a best evidence synthesis to synthesize the results.^[21]^ The studies were grouped based on the type of intervention they utilized, the infectious agent that caused CNS infection, and the cognitive outcome(s) measured used in clinical trials. Data conversions were not feasible as all studies reported changes in different domains and/or subdomains of cognition and used different measures (Table S2, Supplemental Digital Content, http://links.lww.com/MD/J200). Changes in scores from pre- to post-intervention in each of the reported domains and sub-domains were reported as follows: (i) “+” indicating improvement; (ii) “−” indicating decline; and (iii) “=” indicating no change. Aggregated changes reported in each study, by cognitive domain and sub-domains, were presented visually. To explore the extent and causes of heterogeneity, the study population, intervention dose(s), and duration of application were narratively synthesized. Studies with extensive missing data were noted, and methods used within those studies to handle missing data were reported.

### 2.7. Reporting biases and certainty assessment

The certainty of evidence was assessed using the Grading of Recommendations Assessment, Development, and Evaluation (GRADE) system.^[22]^ The GRADE system downgrades the evidence by evaluating the extent of study limitations, including risk of bias, inconsistency of effects, imprecision, indirectness, and publication bias.^[22]^ The certainty of evidence can also be upgraded by evaluating the extent of large effects, dose responses, and explanation of plausible confounding effects.^[22]^ The certainty of the evidence was judged as high, moderate, low, or very low.

### 2.8. Sensitivity analysis

We carried out sensitivity analyses to test whether critical methodological factors or decisions affected the results. We grouped the main results by study design (RCTs or NRS) and compared the results of studies that focused on the same outcome, stratified by infectious agent, and intervention characteristics (Table S2, Supplemental Digital Content, http://links.lww.com/MD/J200). We then organized results based on overall certainty, considering risk of bias, inconsistency, indirectness, imprecision, publication bias, and sample size (Table 5).

**Table 5:** Evaluation of the quality of evidence using the GRADE guidelines.

Anticipated effect	Certainty assessment						
Pharmacotherapy	Risk of bias	Inconsistency	Indirectness	Imprecision	Publication bias	Sample size (number of studies)	Certainty of evidence (GRADE)
Antiviral (valacyclovir)	Serious risk of bias	Some inconsistency exists	Some indirectness exists	Some imprecision exists	Undetected	256 (3)	Low
Antibiotics (ceftriaxone, doxycycline, amoxicillin, other)	Serious risk of bias	Some inconsistency exists	Some indirectness exists	Some imprecision exists	Undetected	521 (5)	Low
Non-opioid analgesic (flupirtine maleate)	Serious risk of bias	Some inconsistency exists	Some indirectness exists	Some imprecision exists	Undetected	28 (1)	Very low

### 2.9. Ethical review

This study did not involve primary data collection; therefore, ethical approval was not sought.

## 3. Results

### 3.1. Study selection

**Figure 1.**
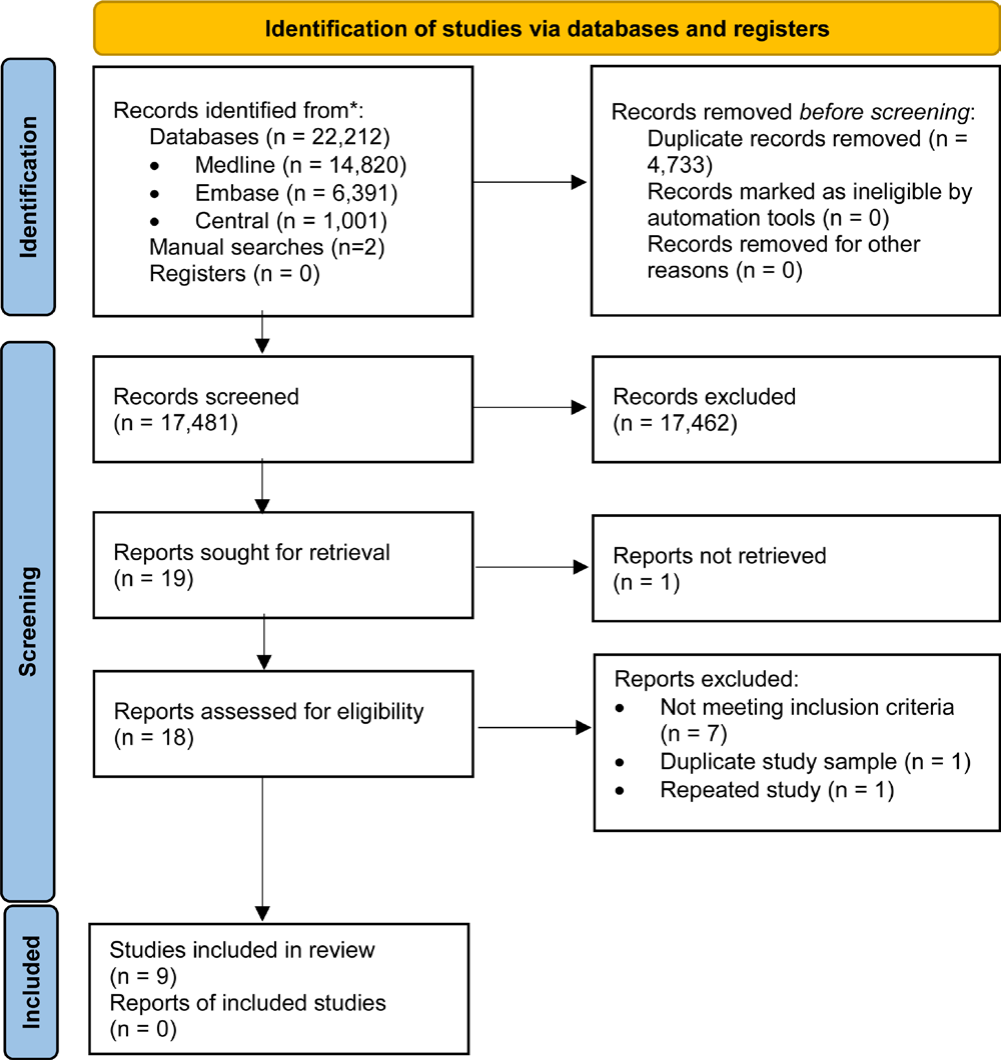
Flow chart documenting process of article selection.

The search strategy procedure yielded a total of 22,212 citations (i.e., MEDLINE n = 14,820; Embase n = 6391; and CENTRAL n = 1001) (see Fig. 1). After screening titles and abstracts, 240 studies were considered relevant and were screened for inclusion criteria, of which 221 articles did not meet the inclusion criteria and were excluded. The remaining 17 articles^[17,23–38]^ and 2 articles^[39,40]^ found from manual searches of citations of included studies, underwent full-text review (Table S3, Supplemental Digital Content, http://links.lww.com/MD/J201). Out of the 19 articles assessed, 9 studies met inclusion criteria and were selected for data synthesis.^[17,23–25,29,31,32,34,36]^

### 3.2. Study characteristics

Table 1 summarizes the data extracted from included studies. Out of the 9 studies, 8 were RCTs^[23–25,29,31,32,34,36]^ and one was NRS of intervention.^[17]^

**Table 1:** Participant demographic and study intervention characteristics.

Author (year)	Infectious agent	Drug (Dosage/frequency, mode of administration, duration of treatment, change over time)	Timeline of clinical trial (wk)	Group	Sample size (n)	Attrition rate (%)	Age		% M	Duration of illness (yr)		Range (yr)
							Mean	SD		Mean	SD	
Prasad et al (2013)^[36]^	HSV1	VAV (1 g/PO BID for 2 wk, ↑ to 1.5 g/PO BID)	18	G1: VAV + AP	12	25	29.54	9.44	50	3.49	2.82	18.89–44.12
				G2: PLA (CD) + AP	12	33	28.67	8.47	58	5.59	3.08	20.19–50.38
Berende et al (2019)^[23]^	LD	CTX (2000 mg/QD, IV for 12 d, followed by DOX (100 mg/PO BID for 12 wk), CLR (500 mg/PO BID for 12 wk) + HCQ (200 mg/PO BID for 12 wk), or PLA.	14	G1: CTX + DOX	86	23	48.3	12.6	54	2.7*		CD
				G2: CTX + CLR-HCQ	96	25	47.5	13	57	2.8*		
				G3: CTX + PLA (Sugar Pills)	98	26	50.3	9.9	52	2.3*		
Breier et al (2018)^[25]^	HSV1	VAV (1.5 g/PO BID, No change)	16	G1: HSV1+ & PLA (Sugar Pills)	40	32.5	30	6.2	65	4.4	2.3	18–40
				G2: HSV1+ & VAV	34	26.5	29.9	6.1	58.8	4.2	2.7	
				G3: HSV1- & PLA (Sugar Pill)	46	30.4	25.4	4.9	89.1	3.6	2.1	
				G4: HSV1- & VAV	50	24	27.4	6	76	3.3	1.9	
Fallon et al (1999)^[17]^	LD	AB (varied in choice and duration in oral and IV – PO (DOX, MIN, AMOX, PCN, AZM, CLR, CXM, and CFE), IM constant (BPG), IV (IPM, CTX, CRO, and VAN), at least 10 d, no change)	16	G1: No Treatment	5	CD	42.7	13.25	30	1.776	1.85	20–65
				G2: Oral AB	7							
				G3: IV AB	7							
				G4: IM AB	4							
Fallon et al (2008)^[29]^	LD	CRO (2 g/IV QD, 10 wk, no change)	10	G1: IV CTX	23	13	45.3	13.7	39	9	6.8	18–65
				G2: IV PLA	14	14	44.8	12.7	42.9			
Bhatia et al (2018)^[24]^	HSV1	VAV (1.5 g/PO BID, 16 wk, no change)	16	G1: AP + VAV	30	17	31.77	8.55	50	4.87	2.01	18–50
				G2: AP + PLA (CD)	32	3.1	30.75	8.68	56	4.96	2.35	
Kaplan et al (2003)^[31]^	LD	CRO (2 g/IV QD, 30 d) followed by DOX (200 mg/PO QD, 60 d)	13	G1: LD(+) & AB	39	23	54	13.7	57.1	3.9	2.7	> 18
				G2: LD(+) & PLA (IV & PO)	39	31						
				G3: LD(−) & AB	25	20	51.1	12	41.6	4.19	3.2	
				G4: LD(−) & PLA (IV & PO)	26	19						
Krupp et al (2003)^[32]^	LD	DOX (100 mg/PO BID, 3 wk, no change) or AMOX (500 mg/PO TID, 3 wk, no change) or CRO (2 g/IV QD, 3 wk, no change)	4	G1: DOX or AMOX or CRO	28	29	48	11.8	46.4	CD		18–70
				G2: PLA (IV)	24	29	47	9.7	48.2			
Otto et al (2004)^[34]^	CJD	FLU (100 mg/PO QD, ↑ 100 g/PO TID or QID, medication stopped when patients no longer fulfilled the inclusion criteria†)	G1: 6 G2: 5	G1: FLU	13	7.7	57	9.6	61.5	CD		35–68

AB = antibiotic, ADAS-Cog = Alzheimer’s Disease Assessment Scale–Cognitive, AMOX = amoxicillin, AP = anti-psychotics, AZM = azithromycin, BID = twice a day, BPG = benzathine penicillin G, CD = cannot determine, CFE = Cefixime, CJD = Creutzfeldt-Jakob Disease, CLR = clarithromycin, CRO = ceftriaxone, CTX = ceftriaxone, CXM = Cefuroxime, DOX = doxycycline, FLU = flupirtine maleate, G = group, GoeCJDDT = Goettingen CJD Dementia Test, HCQ = hydroxychloroquine, HSV1 = herpes simplex virus, type 1, IM = intramuscular, IPM = imipenem, IV = intravenous, LD = Lyme disease, MIN = minocycline, PCN = penicillin, PLA = placebo, PO = by mouth, QD = once a day, VAN = vancomycin, VAV = valacyclovir.

* Median Stated.

† Inclusion and continuation in the study, patients had to achieve a score of at least 50% in 2 of 12 subtests of the dementia tests (ADAS-Cog, GoeCJDDT).

#### 3.2.1. Country of study origin.

The country of study origin for each study is summarized in Table 2. Within the 9 studies, 6 studies were conducted in the United States of America,^[17,25,29,31,32,36]^ one study was conducted in India,^[24]^ one in Germany,^[34]^ and one in the Netherlands.^[23]^

**Table 2:** Studies that have examined the efficacy of drugs in patients with cognitive deficits stemming from primary infection of the central nervous system: summary of study.

Authors (year), country/cohort/ infectious agent	Sample size/age (mean ± SD) yr/sex (%M)/duration of illness (DI, yr)/attrition (by group)	Intervention type, drug, duration (by groups)	Outcome/assessment	Findings/adverse effects
Prasad et al (2013),^[36]^ USA/Western Psychiatric Inst. & Clinic, Pittsburgh, & Wayne State University, Detroit/ HSV-1	G1 (n = 12) Age: 29.54 ± 9.44 Sex: 50% M DI: 3.49 ± 2.82 Attrition = 25% G2 (n = 12) Age: 28.67 ± 8.47 Sex: 58% M DI: 5.59 ± 3.08 Attrition = 33%	Double-blind PLA-RCT G1: VAV + AP G2: PLA + AP/ 18 wks	Working memory (total & 2-back), Immediate verbal memory (processing speed & accuracy)/ PennCNB	G1 vs G2: ↑ in working + verbal memory & visual obj learning/ G1: constipation, stomach pain, motion sickness, occasional muscle twitch, tremor, and upset stomach G2: leg cramps Reported within both groups: drooling, muscle tightness, mild tremors, akathisia, bloating of the stomach, feeling tired, elbow pain, increased sexual drive, insomnia
Berende et al (2019),^[23]^ Netherlands/Sint Maartenskliniek & Radboud University Medical Center/LD	G1 (n = 86) Age: 48.3 ± 12.6 Sex: 54% M DI: 2.7* Attrition = 23% G2 (n = 96) Age: 47.5 ± 13.0 Sex: 57% M DI: 2.8* Attrition = 25% G3 (n = 98) Age: 50.3 ± 9.9 Sex: 52% M DI = 2.3* Attrition = 26%	PLA-RCT G1: CTX + DOX (NR) G2: CTX + CLR-HCQ (NR) G3: CTX + PLA 14 wks	Episodic memory, Attention/working memory, Verbal fluency, Speed Of info processing, Executive function/ RAVLT, DST, TMT-A, SCWT, DSST & CFT	↑ cog performances at wk 14, 26, & 40 compared to BL: not specific to a TX group. LT AB TX ≠ better cog performance than ST AB TX/NR
Breier et al (2018),^[25]^ USA/Indiana University School of Medicine/HSV-1	G1 (n = 40) Age: 30.0 ± 6.2 Sex: 65% M DI: 4.4 ± 2.3 Attrition = 32.5% G2 (n = 34) Age: 29.9 ± 6.1 Sex: 58.8% M DI: 4.2 ± 2.7 Attrition = 26.5% G3 (n = 46) Age: 25.4 ± 4.9 Sex: 89.1% M DI: 3.6 ± 2.1 Attrition = 30.4% G4 (n = 50) Age: 27.4 ± 6.0 Sex: 76% M DI: 3.3 ± 1.90 Attrition = 24%	Double-blind PLA-RCT G1: HSV+ & PLA G2: HSV+ & VAV G3: HSV- & PLA G4: HSV- & VAV 16 wks	Working memory, Visuospatial memory, Letter-number sequencing, Spatial span/ MATRICS & MCCB	VAV failed to demonstrate SGNFT treatment effects on the 2 primary cog outcome measures: the MCCB working memory composite & visuospatial memory scores. VAV add-on therapy may be beneficial for cog impairments but not psychotic SX/ NR but 18.8% discontinued due to “adverse event”
Fallon et al (1999),^[17]^ USA/ NR/ LD	G1 (n = 5) Attrition = CD G2 (n = 7) Attrition = CD G3 (n = 7) Attrition = CD G4 (n = 4) Attrition = CD Age: 42.7 ± 13.25 Sex: 30% M DI: 1.78 ± 1.85	Uncontrolled Study G1: No Treatment G2: Oral AB (NR) G3: IV-AB (NR) G4: IM-AB (NR) 16 wks	Verbal memory, Visual memory Attention, Delayed memory, General memory, Verbal fluency/ WAIS, WMS, & COWAT	T2: G1 overall & individual cog score ↑ than G2; G3 greatest ↑ in cog; No SGNFT correlation b/w DOT on AB & ↑ composite z-score; Repeated AB treatment may ↑ cog in pts/ NR
Fallon et al (2008),^[29]^ USA/New York State Psychiatric Institute and Columbia University Medical Center/LD	G1 (n = 23) Age: 45.3 ± 13.7 Sex: 39% M Attrition = 13% G2 (n = 14) Age: 44.8 ± 12.7 Sex: 42.9% M Attrition = 14% DI: 9.0 ± 6.8	PLA-RCT G1: IV CTX G2: IV PLA 10 wks	Motor, Psychomotor, Attention, Verbal memory, Visual memory, Working memory, Fluency/ WMS- III, N-Back Test, BVRT, BSRT, LRT, CPT, Stroop task, COWAT, CFT, finger tapping, SRT, CRT, TMT-A, TMT-B & digital symbol	12 wk: G1 ↑ in all cog domains; 24 wk: G1 & G2 ↑ in cog; IV CTX = ST ↑ in cog for LD pts/ G1: thrombus, hemolytic anemia, 3 additional Pts discontinued due to “adverse event” G2: Systemic infection, intolerable joint pain
Bhatia et al (2018),^[24]^ India/ Dr Ram Manohar Lohia Hospital, Delhi/ HSV-1	G1 (n = 30) Age: 31.77 ± 8.55 Sex: 50% M DI: 4.87 ± 2.01 Attrition = 17% G2 (n = 32) Age: 30.75 ± 8.68 Sex: 56% M DI: 4.96 ± 2.35 Attrition = 3.1%	PLA-RCT G1: AP + VAV G2: AP + PLA 16 wks	Abstraction & mental flexibility, Attention, Face memory, Spatial memory, Working memory, Spatial ability, Sensorimotor, Emotion/ PennCNB & EMOD	BL: No SGNFT differences b/w G1 & G2 cog functions except spatial ability; Group 1 ↑ in EMOD but not other cog functions/ G1: thrombus, hemolytic anemia, 3 additional Pts discontinued due to “adverse event” G2: Systemic infection, intolerable joint pain
Kaplan et al (2003),^[31]^ USA/ NR/ LD	G1 (n = 39) Attrition = 23% G2 (n = 39) Attrition = 31% G3 (n = 25) Attrition = 20% G4 (n = 26) Attrition = 19% Age: >18 Sex: 51% M DI: 4.01 ± 2.89	Double-blind PLA-RCT G1: LD(+) & AB (2g/d) G2: LD(+) & PLA G3: LD(−) & AB G4: LD(−) & PLA 13 wks	Attention & Speed of info processing, Learning & memory, Word fluency/RAVLT, BVRT, SDMT, & CalCAP	↑ cog functioning not specific to group, treatment, or interaction effects; Sero(+) & sero(−) Pts ↑ cog functioning; Additional AB = no ↑ cog function/ NR
Krupp et al (2003),^[32]^ USA/ Suffolk County, Long Island/ LD	G1 (n = 28) Age: 48.0 ± 11.8 Sex: 46.4% M DI: NR Attrition = 29% G2 (n = 24) Age: 47.0 ± 9.7 Sex: 48.2% M DI: NR Attrition = 29%	Double-blind PLA-RCT G1: AB G2: PLA 4 wk	Cog processing (mental) speed/ AAT	6 mo FU: no SGNFT ↑ cog function or group differences; AB treatment ≠ ↑ cog performance in pts w/ LD/ G1: Diarrhea, anaphylaxis, minor allergic reaction G2: IV sepsis
Otto et al (2004),^[34]^ Germany/German National CJD Surveillance Unit, Goettingen/CJD	G1 (n = 13) Age: 57.0 ± 9.6 Sex: 61.5% M DOI: NR Attrition = 7.7% G2 (n = 15) Age: 61.0 ± 10.3 Sex: 50.0% M DOI: NR Attrition = 13%	Double-blind PLA-RCT G1: FLU G2: PLA 5.5 wk	Long-term & short-term memory, Attention, Executive function, Language, Spatial processing/ADAS, MMSE & GoeCJDDT	G1 had SGNFT in ↓ cog deficits than G2; FLU has SGNFT effects on cog in pts w/ CJD/ G1: muscle weakness G2: lack of tolerability Unspecified group: gastrointestinal bleeding, urticaria

Significant: statistically significant effect was detected (*P* < .05); Non-significant trend: effect was noted however no statistically significant result was detected.

AAT = alpha-arithmetic test, AB = antibiotics, ADAS = Alzheimer’s Disease Assessment Scale, AP = Antipsychotics, Auditory Verbal learning test, BL = baseline, BSRT = Buschke Selective Reminding Test, BVRT = Benton Visual Retention Test, CalCAP = California Computerized Assessment Package, CFT = Category Fluency Test, CLR-HCQ = clarithromycin-hydroxychloroquine, cog = cognitive/cognition, COWAT = Controlled Oral Word Association Test, CPT = Continuous Performance Test, CRT = Choice Reaction Time, CTX = ceftriaxone, DI = duration of illness, DOT = duration of time, DOX = doxycycline, DSST = Symbol-Digit Substitution Test, DST = Digit Span Test, EMOD = Emotion Identification & Discrimination, F = female(s), FLU = Flupirtine maleate, FU = follow-up, G = group, GoeCJDDT = Goettingen CJD Dementia Test, HSV-1 = herpes simplex virus 1, IM = Intramuscular, Inst. = Institute, ITT = intention to treat, IV = intravenous, LD = lyme disease, LRT = Logical Reasoning Test, LT = long-term, M = male(s), MATRICS = Measurement and Treatment Research to Improve Cognition in Psychosis, MCCB = MATRICS Consensus Cognitive Battery, MMSE = Mini-Mental State Examination, NR = not reported, Obj = object, PennCNB = penn computerized neurocognitive battery, PLA = placebo, Pts = patients, RAVLT = Rey, RCT = randomized controlled trial, SCWT = Stroop Color-Word Test, SDMT = Symbol Digit Modalities Test, SGNFT = significant, SRT = Simple Reaction Time, ST = short-term, SX = symptoms, TMT-A = Trail Making Test Part A, TMT-B = Trail Making Test Part B, TX = Treatment, VAV = Valacyclovir, WAIS = Wechsler Adult Intelligence Scale, WMS = Wechsler Memory Scale.

* Median stated.

### 3.3. Sample characteristics

Sample sizes of the studies varied from 23^[17]^ to 280^[23]^ patients. The mean age of participants (in years) within the 9 studies varied from 28^[25]^ to 59,^[34]^ with a mean age (standard deviation (SD)) of 42.67 (10.5) years across the included studies. The percentage of male patients varied from 30%^[17]^ to 72%,^[25]^ with a mean percentage of male patients of 50.7% across the 9 studies. The duration (in years) of primary CNS infections varied from 1.8^[17]^ to 9,^[29]^ with a mean (SD) duration of 4.69 (3.16) across the 9 studies. Two studies did not report the duration of CNS infections,^[32,34]^ while one study reported the median duration of the disease.^[23]^

### 3.4. Interventions in patients with LD

Five studies involving patients with Lyme disease (LD) utilized antibiotics to reduce cognitive impairments.^[17,23,29,31,32]^ Three of these studies used ceftriaxone (e.g., a broad-spectrum third-generation cephalosporin antibiotic that has a long half-life and broader and stronger gram-negative coverage than first or second-generation cephalosporins) through intravenous (IV) administration in comparison to IV placebo.^[29,31,32]^

Fallon et al^[29]^ utilized 15 outcome measures to evaluate 5 cognitive domains (i.e., complex attention, executive functioning, learning and memory, language, perceptual-motor ability) at baseline and then at a follow-up at 12 and 24 weeks. The treatment, placebo, and healthy participant groups were similar in age, gender, premorbid intelligence quotient (IQ), and the treatment and placebo groups were of similar duration of LD. There were noticeable differences between treatment and placebo group compared to healthy participants in the Wechsler Memory Scale–3rd Edition ^[41]^ scores for immediate memory and delayed (general) memory. Improved cognitive scores favoring treatment (i.e., IV ceftriaxone) as compared to the placebo and healthy groups were reported at week 12. At week 24, there were no significant differences between the treatment, placebo and healthy control groups.

Kaplan et al^[31]^ investigated the efficacy of IV ceftriaxone followed by oral doxycycline (e.g., a broad-spectrum tetracycline-class antibiotic, long half-life, used in the treatment of infections caused by bacteria and certain parasites with an anti-inflammatory action). Before receiving treatment, baseline assessment of cognitive function was performed via for the neuropsychological tests; follow-up assessment took place 90 and 180 days (~13 and 26 weeks) after treatment implementation. Cognitive function was measured using 4 outcome measures that covered 4 cognitive domains (i.e., complex attention, executive functioning, learning and memory, and perceptual-motor ability). There was no significant difference between the treatment and placebo groups at baseline in age, sex ratio, scores for the neuropsychological tests, and duration of the disease. Comparing cognitive scores at follow-up showed no differences between the treatment group and the placebo.

Krupp et al^[32]^ investigated the efficacy of IV ceftriaxone in patients with LD on cognitive function and fatigue. Mental speed was assessed using the Alpha-arithmetic Test^[42]^ at baseline and then at a follow-up assessment after 6 months (~36 weeks). Baseline characteristics, including sample size, age, sex ratio, and LD symptoms, did not differ between treatment, placebo, and healthy controls groups. No group differences at follow-up assessment between treatment and placebo were reported. Mental speed processing was lower in treatment and placebo groups than in healthy controls.

Berende et al^[23]^ utilized the IV ceftriaxone and doxycycline or IV ceftriaxone and a combination of clarithromycin with hydroxychloroquine (e.g., disease-modifying antirheumatic drugs decreasing the activity of the immune system, used as antimalarials) in comparison to IV ceftriaxone and placebo. Changes in cognitive function from baseline to follow-up assessment at 14, 26, and 40 weeks were assessed using 6 outcome measures which measured 4 different cognitive domains (i.e., complex attention, executive functioning, learning and memory, perceptual-motor ability). Baseline characteristics, including sample size, age, sex ratio, and LD symptoms, did not differ between the treatment and placebo groups. The study reported improvements in several cognitive domains at 14, 26, and 40 weeks; this improvement was not specific to the treatment group.

The NRS of intervention by Fallon et al^[17]^ utilized various antibiotics administered orally, IV, or intramuscularly compared to the untreated persons with LD. Cognitive function was measured using 3 outcome measures covering 6 cognitive domains (i.e., complex attention, executive functioning, learning and memory, language, perceptual-motor ability, and social cognition) at the baseline and after 4 months (~16 weeks). The groups did not differ in baseline cognitive performance, anxiety, and depression scores. Compared to the placebo group, the group administered antibiotics showed significant overall and individual cognitive performance improvement at follow-up; IV administered antibiotics showed the greatest cognitive improvement. No improvement was observed in the non-treated persons.

### 3.5. Intervention in patients with HSV-1

Three studies reported on the use of antiviral drugs to target cognitive impairments in persons with herpes simplex virus type 1 (HSV-1).^[24,26,36]^ One study^[26]^ studied the efficacy of 16 weeks of valacyclovir administration compared to placebo in HSV-1 positive and HSV-1 negative groups. Two studies^[24,36]^ used valacyclovir (e.g., an antiviral drug that inhibits viral DNA polymerase, incorporates into and terminates the growing viral DNA chain, and inactivates the viral DNA polymerase) in combination with antipsychotics and compared results to placebo in combination with antipsychotics. Bhatia et al^[24]^ reported on specifics of antipsychotics used (i.e., trihexyphenidyl), whereas Prasad et al^[43]^ did not specify the antipsychotic drug.

Bhatia et al^[24]^ assessed cognitive functioning using 2 outcome measures (i.e., Penn Computerized Neurocognitive Battery (PennCNB)^[43]^ and Emotion Identification & Discrimination test^[44]^ which measured 6 different cognitive domains at baseline and then at a follow-up assessment after 4 and 5 months (~20 weeks). Baseline characteristics, including clinical variables, demographics, and cognitive status, except spatial ability (i.e., better in the treatment group than placebo), did not differ between the treatment and placebo groups. Comparison of cognitive function scores showed no group differences in the PennCNB at follow-up assessment between treatment and placebo groups, whereas only patients that received the treatment significantly improved the Emotion Identification & Discrimination accuracy index scores.

The study by Prasad et al^[43]^ evaluated change in 6 cognitive domains (i.e., complex attention, executive functioning, learning and memory, language, perceptual-motor ability, language, and social cognition) using the PennCNB. Cognitive performance was assessed at baseline and then at a follow-up 18 weeks later. The treatment and placebo groups were similar in age, and duration of illness with respect to baseline demographics. There were differences between the groups in Positive and Negative Syndrome Scale scores, with the placebo group scoring higher. Improvement in verbal memory, visual object learning, and working memory favoring treatment valacyclovir group compared to the placebo was reported at follow-up.

Breier et al^[26]^ assessed 4 cognitive domains (i.e., executive functioning, learning and memory, perceptual-motor ability, and language using the Measurement and Treatment Research to Improve Cognition in Schizophrenia Consensus Cognitive Battery.^[45]^ The study reported no significant differences in cognitive performance on any cognitive domains for the treatment group at follow-up compared to baseline.^[26]^

### 3.6. Interventions in patients with CJD

One study tested the analgesic drug flupirtine maleate’s efficacy compared to the placebo in patients with LD.^[34]^ Five cognitive domains (i.e., complex attention, executive functioning, learning, and memory, language, and perceptual-motor ability) were assessed using the Alzheimer’s Disease Assessment Scale–Cognitive Subscale,^[46]^ the Mini-Mental State Examination,^[47]^ and the Goettingen Creutzfeldt Jakob disease (CJD) Dementia Test^[34]^ at baseline and then at a follow-up assessment 2, 4, 8, 12, 16, and 20 weeks after treatment administration. There were no significant differences between the treatment and placebo groups in age, CJD clinical signs, and cerebrospinal fluid at the baseline. The treatment group showed greater improvement in all studied domains of cognition at follow-up as compared to the placebo.

### 3.7. Outcome measures

Summarized data results of outcome measures used across the included studies are reported in Table 3. Primary outcomes of interventions in patients with LD,^[17,23,29,31,32]^ HSV-1,^[24,26,36]^ and CJD^[34]^ included complex attention, executive functioning, learning and memory, language, perceptual-motor ability and social cognition.^[16]^

**Table 3:** Summary of the cognitive outcome measures utilized across the studies and the cognitive domains they measure.

Author	Year	Tools used to measure cognitive domain	Type of cognitive domain
Prasad et al^[36]^	2013	PennCNB	Verbal memory, visual short-term memory, episodic memory, general memory, delayed memory, long-term memory, short-term memory
Berende et al^[23]^	2019	RAVLT, DGST	
Breier et al^[25]^	2018	MCCB	
Fallon et al^[17]^	1999	WMS	
Fallon et al^[29]^	2008	WMS-III, BVRT, BSRT, LRT; N-Back Test	
Bhatia et al^[24]^	2018	PennCNB	
Kaplan et al^[31]^	2003	RAVLT, BVRT	
Otto et al^[34]^	2004	ADAS-Cog, GoeCJDDT, MMSE	
Prasad et al^[36]^	2013	PennCNB	Emotion processing
Bhatia et al^[24]^	2018	PennCNB, EMOD	
Berende et al^[23]^	2019	TMT-A, SCWT, SDST	Speed of information/cognitive processing
Kaplan et al^[31]^	2003	SDMT, CalCAP	
Krupp et al^[32]^	2003	AAT	
Fallon et al^[17]^	1999	WMS	Attention
Fallon et al^[29]^	2008	CCPT, SCWT	
Bhatia et al^[24]^	2018	PennCNB	
Kaplan et al^[31]^	2003	SDMT, CalCAP	
Otto et al^[34]^	2004	ADAS-Cog, GoeCJDDT, MMSE	
Berende et al^[23]^	2019	CFT	Language and fluency (verbal, word)
Fallon et al^[17]^	1999	COWAT	
Fallon et al^[29]^	2008	COWAT, CFT	
Kaplan et al^[31]^	2003	RAVLT, BVRT	
Otto et al^[34]^	2004	ADAS-Cog, GoeCJDDT, MMSE	
Prasad et al^[36]^	2013	PennCNB	Perceptual-motor, social cognition
Bhatia et al^[24]^	2018	PennCNB	
Fallon et al^[29]^	2008	FTT, SRT, CRTT, TMT part A and B; SDST	
Otto et al^[34]^	2004	MMSE	
Berende et al^[23]^	2019	TMT IS, SCWT IS	Executive function
Otto et al^[34]^	2004	ADAS-Cog, GoeCJDDT	

AAT = Alpha Arithmetic Test, ADAS-Cog = The Alzheimer’s Disease Assessment Scale–Cognitive test, BSRT = Buschke Selective Reminding Test, BVRT = Benton Visual Retention Test, CalCAP = California Computerized Assessment Package, CCPT = Continuous Performance Test, CFT = Category Fluency Test, COWAT = Controlled Oral Word Association Test, CRTT = Choice Reaction Time Task, DGST = Digit Span Test, EMOD = Emotion Identification and Discrimination, FTT = Finger Tapping Test, GoeCJDDT = Goettingen CJD Dementia Test, IS = Interference Score, LRT = Logical Reasoning Test, MCCB = MATRICS Consensus Cognitive Battery, MMSE = Mini-Mental State Test, PennCNB = Penn Computerized Neurocognitive Battery, RAVLT = Rey Auditory Verbal Learning Test, SCWT = Stroop Color-Word Test, SDMT = Symbol Digit Modalities Test, SDST = Symbol-Digit Substitution Test, SRT = Simple Reaction Time Task, TMT-A = Trail Making Test part A, WMS = Wechsler Memory Scale.

Across the 9 studies, 29 outcome measures were used, of which 2 assessed all 6 cognitive domains: the PennCNB,^[43]^ utilized in 2 studies,^[24,36]^ and the Wechsler Adult Intelligence Scale (WAIS),^[48]^ utilized in one study.^[17]^ The rest of the studies assessed one or more cognitive function domains using various outcome measures. The 3 most frequently evaluated cognitive domains were language, assessed within 8 studies using 7 different outcome measures;^[17,23,25,29,31,32,34,36]^ complex attention, assessed within 7 studies using 19 different outcome measures;^[17,23,24,29,31,32,34]^ and learning and memory, assessed within 7 studies using 10 different outcome measures.^[17,23–25,29,31,34]^ The least evaluated cognitive domain was social cognition, assessed within one study.^[24]^

### 3.8. Response to intervention by cognitive domain

Summarized data results of changes in different cognitive domains across the included studies are reported in Table 4. For detailed data results of changes in cognitive function from baseline to follow-up(s) in different cognitive domains across the included studies please refer to (Table S4, Supplemental Digital Content, http://links.lww.com/MD/J202).

**Table 4:** Summarized data of changes in different cognitive domains across studies.

Author (year)	Infectious agent	Complex attention	Executive functioning	Learning and memory	Language	Perceptual-motor ability	Social cognition
Prasad et al (2013)^[36]^	HSV-1		+		+		
Breier et al (2018)^[25]^	HSV-1		=	=	=	=	
Bhatia et al (2018)^[24]^	HSV-1	=	=	=		=	+
Otto et al (2004)^[34]^	CJD	+	+	+	+	+	
Berende et al (2019)^[23]^	LD	+	=	=	+		
Fallon et al (1999)^[17]^	LD	=		+	+		
Fallon et al (2008)^[29]^	LD	+	+	+	+	+	
Kaplan et al (2003)^[31]^	LD	+		+	+		
Krupp et al (2003)^[32]^	LD	=			=		

Changes in different cognitive domains reported from baseline to the last follow-up: “=” no significant change; “+” improvement; “no marks” not tested.

CJD = Creutzfeldt-Jakob disease, HSV-1 = herpes simplex virus, LD = Lyme disease.

#### 3.8.1. Complex attention.

The efficacy of pharmacotherapy on complex attention was assessed in 7 studies.^[17,23,24,29,31,32,34]^ Within the domain of complex attention, attention and speed of information processing sub-domains were assessed (Table 4; Table S4, Supplemental Digital Content, http://links.lww.com/MD/J202).

Of 5 RCTs in persons with LD that assessed complex attention,^[17,23,29,31,32]^ two^[31,32]^ assessed both attention and speed of information processing subdomains, 2 studies^[17,29]^ assessed attention sub-domain and one^[23]^ study assessed speed of information processing. Across studies that assessed the sub-domain of attention, the results were inconsistent: 2 studies^[17,32]^ reported no change in response to treatment and 2 studies^[29,31]^ reported change in response to treatment. Results across the attention processing sub-domain were also inconsistent: 2 studies^[23,31]^ reported improvement and one study^[32]^ reported no change in response to intervention (Table 4; Table S4, Supplemental Digital Content, http://links.lww.com/MD/J202).

In the 3 studies on patients with HSV-1, one study^[24]^ assessed complex attention and attention sub-domains. Researchers reported no significant change in scores in response to treatment in patients with HSV-1 disease compared to their baseline scores (Table 4; Table S4, Supplemental Digital Content, http://links.lww.com/MD/J202).

#### 3.8.2. Executive function.

Six studies included in this review^[23–25,29,34,36]^ assessed 4 sub-domains of executive function: working memory, executive function, abstraction, and mental flexibility (Table 4; Table S4, Supplemental Digital Content, http://links.lww.com/MD/J202).

Two out of the 5 studies on persons with LD assessed working memory sub-domain.^[23,29]^ The results were as follows: one study^[29]^ reported an improvement in working memory from baseline to follow-up at 10 weeks and one study^[23]^ reported improvement from baseline to follow-up at weeks 26 and 40 in the ceftriaxone followed by doxycycline treatment group but not in the treatment group that received ceftriaxone followed by clarithromycin and hydroxychloroquine. The latter study also assessed the executive function sub-domain and reported no change across both treatment groups from baseline to follow-up^[23]^ (Table 4; Table S4, Supplemental Digital Content, http://links.lww.com/MD/J202).

Of the 3 studies in persons with HSV-1 that assessed executive function,^[24,25,36]^ all assessed the sub-domain of working memory. Two studies reported no change in the working memory sub-domain from baseline to last follow-up at 22 weeks and 16 weeks, respectively,^[24,25]^ and one study^[36]^ reported improvement in working memory from baseline to follow-up at 18 weeks. One study^[24]^ that assessed abstraction and mental flexibility sub-domain reported no change from baseline in response to treatment (Table 4; Table S4, Supplemental Digital Content, http://links.lww.com/MD/J202).

In the single study of persons with CJD^[34]^ only the sub-domain of executive function was assessed. Researchers reported improvement from baseline in response to flupirtine maleate (Table 4; Table S4, Supplemental Digital Content, http://links.lww.com/MD/J202).

#### 3.8.3. Learning and memory.

Learning and memory were assessed in 7 studies.^[17,23–25,29,31,34]^ Learning and memory cognitive domain assessed in included studies included the following sub-domains: episodic memory, overall composite, visual memory, general memory, delayed memory, and face memory (Table 4; Table S4, Supplemental Digital Content, http://links.lww.com/MD/J202).

Four studies among the 5 on patients with LD evaluated the learning and memory sub-domains.^[17,23,29,31]^ General memory was the only sub-domain that was commonly assessed between the 2 studies.^[17,31]^ Both studies reported improvements in general memory from baseline to follow-up at 17 weeks and 13 weeks, respectively;^[17,31]^ no further change from 13 weeks to 26 weeks was observed.^[31]^ The former study^[29]^ also assessed the overall composite score and reported improvement. An improvement in delayed memory sub-domain but not visual memory sub-domain was reported in one study.^[17]^ One RCT assessed the episodic memory sub-domain and reported improvement from baseline to follow-up at week 26 but no change from baseline to follow-up in weeks 14 and 40 in the ceftriaxone followed by doxycycline treatment group.^[23]^ In the treatment group that received ceftriaxone followed by clarithromycin, the study reported improvement from baseline to follow-up at weeks 14 and 26 but no change in cognition from baseline to week 40 (Table 4; Table S4, Supplemental Digital Content, http://links.lww.com/MD/J202).

Among the 3 studies in persons with HSV-1, 2 assessed learning and memory,^[24,25]^ of which one assessed the sub-domain of face memory^[24]^ and one assessed overall composite memory.^[25]^ Both studies reported no changes in response to intervention (Table 4; Table S4, Supplemental Digital Content, http://links.lww.com/MD/J202).

In the single study of persons with CJD, only the overall composite cognitive score was assessed.^[34]^ Study results indicate improvement in cognition in patients after treatment compared to baseline (Table 4; Table S4, Supplemental Digital Content, http://links.lww.com/MD/J202).

#### 3.8.4. Language.

Efficacy of pharmacotherapy in language domain was evaluated in 8 studies.^[17,23,25,29,31,32,34,36]^ Sub-domains that were evaluated included verbal memory, letter-number sequencing, language, and verbal fluency (Table 4; Table S4, Supplemental Digital Content, http://links.lww.com/MD/J202).

All 5 studies on persons with LD assessed language domain.^[17,23,29,31,32]^ Four of the 5 studies assessed the sub-domain of verbal fluency.^[17,29,31,32]^ Two studies^[29,31]^ showed improvement in verbal fluency from baseline to follow-up measurement, one study^[17]^ reported no change, and one study^[23]^ reported no change from baseline to 14 weeks, but improvement from baseline to follow-up at weeks 26 and 40. Two studies^[17,32]^ assessed the verbal memory sub-domain: one study^[17]^ reported improvement from baseline to 17 weeks and one study^[32]^ reported no change from baseline to follow-up at 26 weeks (Table 4; Table S4, Supplemental Digital Content, http://links.lww.com/MD/J202).

Of the two among the 3 studies on patients with HSV-1 that assessed language,^[25,36]^ one^[25]^ that assessed the letter-number sequencing reported no change from baseline to follow-up and one study^[36]^ that assessed verbal memory reported change in response to treatment (Table 4; Table S4, Supplemental Digital Content, http://links.lww.com/MD/J202).

In the study of persons with CJD,^[34]^ verbal fluency and language were assessed. The study results indicated an improvement in language and no change in verbal fluency at follow-up compared to baseline (Table 4; Table S4, Supplemental Digital Content, http://links.lww.com/MD/J202).

#### 3.8.5. Perceptual-motor ability.

The perceptual-motor ability was evaluated in 4 studies.^[24,25,29,34]^ Sub-domains within the perceptual-motor ability domain included visuospatial memory, spatial ability, motor, psychomotor, sensorimotor, and spatial memory (Table 4; Table S4, Supplemental Digital Content, http://links.lww.com/MD/J202).

Among the 5 studies that included patients with LD, one study^[29]^ assessed perceptual-motor ability, specifically the motor and psychomotor sub-domains, and reported improvement across both sub-domains at follow-up compared to baseline assessment (Table 4; Table S4, Supplemental Digital Content, http://links.lww.com/MD/J202).

In the 3 studies on patients with HSV-1, 2 studies evaluated perceptual-motor ability.^[24,25]^ One study^[24]^ assessed spatial memory and sensorimotor ability, and one^[25]^ assessed visuospatial memory and spatial ability. Both studies^[24,25]^ reported no change in follow-up compared to baseline across all sub-domains within the perceptual-motor ability cognitive domain (Table 4; Table S4, Supplemental Digital Content, http://links.lww.com/MD/J202).

Only the spatial ability sub-domain was assessed in the study of persons with CJD.^[34]^ Study results indicated that spatial ability performance improved in patients after treatment compared to baseline assessment (Table 4; Table S4, Supplemental Digital Content, http://links.lww.com/MD/J202).

#### 3.8.6. Social cognition.

The efficacy of pharmacotherapy on social cognition was assessed in one study.^[24]^ Researchers evaluated the sub-domain of emotion within the social cognition domain and reported an improvement in emotional recognition scores at follow-up compared to a baseline assessment in response to pharmacotherapy (Table 4; Table S4, Supplemental Digital Content, http://links.lww.com/MD/J202).

### 3.9. Risk of bias in studies and certainty of the evidence

One study had an overall “low” risk of bias score^[29]^ and the rest studies scored either “some concerns”^[23,24,32,36]^ or a “high” risk of bias score^[25,31,34]^ (Table S5, Supplemental Digital Content, http://links.lww.com/MD/J203). Across the 5 domains assessed, there was substantial agreement between reviewers (kappa = 0.89). The studies were unclear regarding randomization, allocation, and adherence according to the study’s protocol.^[22]^ A NRS of intervention^[17]^ was of very low quality. According to the GRADE guidelines, the quality of the selected RCT studies was low, and the quality of the NRS of intervention was very low, suggesting low and very low certainty in the reported results (Table 5).^[22]^

### 3.10. Loss to follow-up

In the 3 studies that reported loss to follow-up,^[25,31,32]^ results show that with the intervention completion percentages of 83%,^[25]^ 98%,^[31]^ and 88%^[32]^ the majority of participants completed their treatment protocols. Prasad et al^[36]^ mentioned that loss to follow-up was measured, however, they did not report the information. The 5 remaining studies included no information regarding the completion of the intervention.^[17,23,24,29,34]^

### 3.11. Safety and adverse effects of interventions

Five studies reported adverse effects of interventions (Table 2),^[24,29,32,34,36]^ while the other 3 studies did not report any adverse effects.^[17,23,31]^ The remaining study^[25]^ reported no specific adverse effects but indicated that 18.8% of study participants discontinued treatment due to adverse events (Table S2, Supplemental Digital Content, http://links.lww.com/MD/J200).

## 4. Discussion

This systematic review provides a comprehensive evidence synthesis of intervention studies testing the efficacy of pharmacotherapy for reducing cognitive impairments in patients with primary CNS infections, a relatively unexplored topic. Nine studies comprised of 8 RCTs and one NRS of intervention described the efficacy of antibiotics, antiviral, and non-opioid analgesic drug in adult patients with LD, HSV-1, and CJD, respectively. The present review highlights the limited breadth of existing evidence and sources of variation in response to intervention even with the application of a similar class of medication.

While the available evidence suggests that antibiotics, antivirals, and muscle relaxant might improve certain domains/sub-domains of cognition in patients with LD, HSV-1, or CJD at least temporarily, of the available evidence, only one study^[29]^ had “low” risk of bias. Due to a lack of methodologically high-quality studies, variations in the timing of administration, and heterogeneity in outcome measures used to evaluate cognition, we found it difficult to draw generalizable conclusions about the efficacy of pharmacotherapy on the cognitive outcomes of patients with primary CNS infections. Further investigations are required to develop scientific hypotheses concerning optimal treatment approaches, the length and timing of administration of pharmacotherapy during the course of CNS infection, as well as qualitative insights from people about the functional meaningfulness of improvement in scores on cognitive measures in response to interventions.

### 4.1. Patient population

The lack of consistency between studies, even relating to the same infectious disease of the CNS, can be attributed to a variety of factors, the individual impacts of each a challenge to interpret. To determine whether differences in response to interventions between study samples were attributable to patients’ characteristics such as age, general and brain health, comorbid disorders, or the immune response due to interaction of the infectious agent with immune system of a patient, time since infection, level of impairment at the time of treatment initiation, or a combination of all, was a complex task. At this time the nature and implications of the results have not been adequately explored given limited number of studies and information available within them. Nevertheless, considering the extensive evidence on the complexity of the molecular mechanisms used by pathogens to colonize, invade, infect, and disrupt patients’ brain structures involved in cognitive function,^[49,50]^ the response to a variety of therapeutic strategies described in this review is likely to be influenced by all of the above. Consequently, future research needs to take these considerations into account at the design phase of clinical trials.

### 4.2. Interventions

Cognitive impairments in patients with primary CNS infection may result from damage that occurs through a variety of mechanisms within the brain tissue (i.e., excitotoxic, inflammatory, immune, necrosis, and apoptosis).^[51,52]^ The mechanisms targeted by the studies included in this review focused on reduction of the toxicity by an infectious agent and reducing the extent of secondary injury. These secondary mechanisms evolve over variable periods of time following exposure to an infectious agent, thus providing an opportunity for pharmacological interventions to minimize the long-term effect of the primary CNS infection. Timing of treatment initiation after CNS infection was variable across the studies. Most of the studies^[23–25,29,31,36]^ applied the treatment years after exposure to the infectious agent. The remaining few studies^[32,34]^ did not report such information or applied intervention to patients with variable durations of illness.^[17]^

The duration of the interventions was also variable across studies, depending on the type of CNS infection and a class of pharmacotherapy, with a range between 4 weeks in patients with LD treated with antibiotics^[32]^ and 18 weeks in patients with HSV-1 treated with an antiviral drug.^[36]^ Given the current uncertainty regarding whether cognitive improvement can be sustained in the long term after treatment discontinuation, there is a pressing need for further studies to investigate this important question.

The dosage of similar classes of drugs within the same type of CNS infection appeared to be relatively consistent across the studies. An oral dose between 1000 and 1500 mg of valacyclovir twice a day was the preferred dosage for HSV-1 patients; an intravenous 2000 mg of ceftriaxone alone or followed by an oral dose of doxycycline (100–200 mg once or twice daily) was a preferred dosage for LD patients. A combination of different antibiotic types and the mode of administration in patients with LD was performed in several studies^[17,23,31,32]^ to increase drug tolerability and expand the spectrum of action. In a sole study of patients with CJD, a flupirtine maleate was administered in the dose of 100 mg daily, followed by an increase to 100 g 3 to 4 times daily, with medication stopped when patients no longer fulfilled the inclusion criteria.

The dose utilized in older versus younger patients seems not to differ, and the dose administered in male and female patients was the same. The existent research has not yet brought forth a discussion to address drug efficacy in the youngest and the oldest adult patients with known differences in absorption, distribution, and elimination, as well as the effects of drug interactions when multiple drugs are used concomitantly. Likewise, considering the sex-linked dimorphism of human physiology and gender-linked pathology experience, reflected in drug intake, metabolism, pharmacokinetics, pharmacodynamics, and bioavailability, the activity of pharmaceutical therapy and its effectiveness is expected to be sex- and age-specific. These directions of research remain to be initiated.

In addition, over the past decades, the genome of humans has been decoded, opening the door to the study of the genetic variation that causes differences in drug response due to variability in the activity of a particular enzyme responsible for the elimination of the drug.^[53]^ Finally, adoption studies have shown that the risk of acquiring infections is heritable, and in recent years there is a growing interest in genetic variation in the immune system related to susceptibility and severity of infection and associated impairments. Future studies designing pharmacological interventions in patients with primary CNS infections expect to report on hypothetical correspondence between targets of pharmacological agents and cognitive function.

### 4.3. Outcome measures

The included 9 studies used 29 different cognitive outcome measures, evaluating one or more cognitive domains and sub-domains (i.e., complex attention, executive functioning, learning and memory, language, perceptual-motor ability, and social cognition). Only 2 outcome measures, the PennCNB^[43]^ and the WAIS,^[48]^ utilized in 3 studies,^[17,24,36]^ assessed all 6 cognitive domains. Currently, knowledge of the psychometric properties relevant to an evaluation of change over time (i.e., test-retest reliability, responsiveness to change) of the utilized 29 outcome measures in the population of interest is lacking, which brings up the question of whether the observed improvement was the result of practice effect or the true effect of an intervention. The way forward seems to be agreement on a group of outcome measures with established evaluative properties suitable for clinical trials in patients with primary CNS infections. Consistent use of outcome measures can aid future comparisons between trials, combining data from different sources to arrive at a precise estimate of the effectiveness of interventions. Additionally, there is a need to consider the utility of outcome measures that evaluate changes in cognitive capacity rather than impairment.

### 4.4. Safety and adverse effects

The patients’ adherence to antimicrobial therapy was not reported in the included studies, a significant limitation as tolerability is an important factor for patients and clinicians when making treatment choices.^[54,55]^ Reporting safety concerns is equally important and should not be omitted from consideration in future trials. Some studies have shown that broad-spectrum antimicrobials can adversely affect bodily functions, even after short-term use, in special groups of patients, such as children, the elderly, and patients with degenerative and medical disorders (e.g., cardiovascular, renal, and hepatic diseases),^[56]^ causing confusion, delirium, and decline in attention. Reporting individual patient changes in response to intervention and safety parameters, rather than group reporting, is likely the most effective approach to evaluate changes in cognitive scores and address the cause of variability in response to pharmacotherapy across different patients.

### 4.5. Study strengths and limitations

The strengths of our study include comprehensive coverage of current research findings, careful appraisal of study quality, a focus on clinically relevant endpoints, and comprehensive analyses allowing comparisons between studies.

There are several limitations to this research we wish to acknowledge. The focus of this evidence synthesis was the efficacy of pharmacotherapy in patients with primary CNS infections. Despite attempts to include all clinical trials on the topic, it is possible studies were missed. We excluded studies that focused on cognitive impairments stemming from exposure to chronic infectious agents whose primary targets are not neurotropic tissue, such as human immunodeficiency virus (HIV), hepatitis C virus, and emerging pathogens like severe acute respiratory syndrome coronavirus 2 among others. The significance of studying cognitive impairment stemming from these infectious agents cannot be overstated.

Further limitations include a focus on peer-reviewed English-language studies only, omitting potentially relevant results published in other languages, and non-peer-reviewed works. As such, there is a possibility for publication and results’ generalizability biases. There are also limitations to the presented data on cognitive outcome measures used in the reviewed studies. The evaluation properties of the outcome measures were not reported.

We were not in the position to utilize advanced statistical techniques such as meta-analytic principal component analysis and sparse meta-analytic principal component analysis, which have been suggested to aid in identification patterns and reduce complexity of clinical trial data)^[57–60]^ for several reasons. The high heterogeneity of the data in terms of study design, intervention, population, and outcome measures in principle violates the assumptions of homogeneity in the covariance organization across studies included in this evidence synthesis, as require for principal component analysis. In addition, incomplete and inconsistent data coming from different studies measuring change in similar cognitive domain via different measures requires significant human expertise to transform data into a multivariate format but limited published procedure protocols are available to do so. In the future, with additional research on the topic, it may be feasible to conduct these types of analyses for high-dimensional data for pattern recognition and vizualization of complex clinical data. This would involve careful consideration of assumptions, limitations, and appropriate validation and sensitivity analyses to ensure that the results are reliable and valid.

Finally, we acknowledge that missing data in studies included in this review may have impacted the reliability of our review findings, reflected in the risk of bias and certainty of evidence ratings. We also chose not to exclude the studies with missing data, as we believe in the importance of synthesizing all available evidence with clear critical appraisal and certainty assessment reporting. To mitigate the concern and assess the impact of missing data on our findings, we performed a proxy of qualitative sensitivity analysis. This involved re-grouping the data using different methods to determine the similarities and differences between studies included in this review within different domains of cognition, infectious agents, medication class, and duration of intervention. However, this approach should be interpreted with caution, as it is not a substitute for statistical analyses.

### 4.6. Quality of evidence

We conducted the review using a robust methodology, with at least 2 review authors independently assessing studies for eligibility, extracting data, and carrying out risk of bias assessment. For the evaluation of quality of the evidence, where some of the methodologies were not reported in sufficient detail to enable us to make a judgment, we contacted authors for clarification. We also contacted authors of primary studies when it appeared that similar groups of patients had been described in more than one publication, in order to avoid double counting participants.^[39,40]^ Although the majority of the studies were classified as “randomized,” concerns around the randomization process, deviations from the intended intervention, missing outcome data, measurement of the outcome, and selection bias of the reported results were not consistently addressed across studies.^[17,23–25,29,31,32,34,36]^ The study protocols were not available as publications for any of the included studies except for one study.^[23]^ This increases the risk of reporting bias and impacts the study quality. The Centre for Reviews and Dissemination Guidelines and the Cochrane Reviewers’ Handbook suggest that “quality” is an important consideration when performing reviews, with “quality” relating to the extent to which a study minimizes bias and maximizes internal and external validity. Because there was a limited number of studies to address our research objectives, the decision was made to show all the evidence available, even in light of the high risk of bias and low certainty.

### 4.7. Implications for clinical practice and research

The evidence synthesis results demonstrated only low certainty evidence for the efficacy of pharmacological interventions for dealing with cognitive impairments in adult patients with LD, HSV-1, or CJD. The existing evidence refers only to 3 specific classes of pharmaceuticals: antibiotics (alone or in combination with antipsychotics), antiviral drugs, and non-opioid analgesics, used in patients with LD, HSV-1, and CJD, respectively. Placebo-based comparisons of antibiotics and antivirals demonstrated inconsistent efficacy, utilizing various cognitive outcome measures in patients with LD and HSV-1. The NRS of intervention utilizing non-opioid analgesics in patients with CJD reported improvement, although this was a single study of very low quality. Also worth noting is that one third of included studies came from outside North America – one from India,^[24]^ one from Germany,^[34]^ and one from the Netherlands.^[23]^ While we did not observe patterns based on country of origin, the role of culture in the presentation of and coping approaches to cognitive impairments stemming from CNS infections cannot be fully discarded. These are particularly relevant because the psychometric properties of the outcome measures utilized in the studies included in this review have not been described in cross-cultural communities.

## 5. Conclusions

The limited number of studies on each medication class and infectious agent, high heterogeneity, and inconsistency in the data, including the outcome measures utilized, preclude definite conclusions on the ability of antimicrobials and non-opioid analgesics in reducing cognitive impairments in patients with primary CNS infection. More translational research is needed to characterize cognitive impairment and its early identification in patients with CNS infection and to develop new prognostic indicators of cognitive impairment course, adverse effects, and safety indicators in patients of various ages, sexes, baseline cognitive status, and disease severity. Efforts must also be aimed at assessing the determinants of specific domains of cognitive function that do respond to therapy, to define whether they relate to the characteristics of the patient or the infectious agent, or the timing, the dose, of mode of administration of the intervention, or treatment adherence.

To promote the advancement of clinical trial methodologies, researchers should reach a consensus on outcome measures in clinical trials and on the core endpoints of clinical trials that are relevant to patients. The development and utilization of advanced statistical methodologies that enable digital transformation of heterogeneous data and machine learning techniques to identify hidden patterns in data present promising opportunities for detecting distinct subtypes in patients, leading to the realization of personalized medicine and tailored therapeutic approaches.

## Acknowledgments

We gratefully acknowledge the involvement of Ms. Jessica Babineau, information specialist at the Toronto Rehabilitation Institute University Health Network for her help with the comprehensive literature search.

## Author contributions

**Conceptualization:** Jhankhana S. Shah, Tatyana Mollayeva.

**Data curation:** Jhankhana S. Shah, Sarah Shaaya, Tatyana Mollayeva.

**Formal analysis:** Syeda T. Rizvi, Jhankhana S. Shah, Sarah Shaaya, Tatyana Mollayeva.

**Funding acquisition:** Tatyana Mollayeva.

**Investigation:** Syeda T. Rizvi, Jhankhana S. Shah, Sarah Shaaya, Tatyana Mollayeva.

**Methodology:** Tatyana Mollayeva.

**Project administration:** Syeda T. Rizvi, Jhankhana S. Shah, Sarah Shaaya.

**Resources:** Syeda T. Rizvi, Jhankhana S. Shah, Sarah Shaaya, Tatyana Mollayeva.

**Software:** Jhankhana S. Shah, Sarah Shaaya, Tatyana Mollayeva.

**Supervision:** Tatyana Mollayeva.

**Validation:** Tatyana Mollayeva.

**Visualization:** Syeda T. Rizvi, Tatyana Mollayeva.

**Writing – original draft:** Syeda T. Rizvi.

**Writing – review & editing:** Jhankhana S. Shah, Sarah Shaaya, Tatyana Mollayeva.

## Supplementary Material










