# Supplementary material for: Treating cognitive impairments in primary central nervous system infections: A systematic review of pharmacological interventions
Source: Medicine (Baltimore). 2023 Jul 14;102(28):e34151. doi: 10.1097/MD.0000000000034151 (PMC10344564; doi:10.1097/MD.0000000000034151)
Supplement: Supplementary file 1 [file medi-102-e34151-s001.pdf]

**Table S1. Search Strategy**

Searches were conducted in MEDLINE(R) ALL (in Ovid, including Epub Ahead of Print, In-Process & Other Non-Indexed Citations, Ovid MEDLINE(R) Daily and then translated into Embase (Ovid), and the Cochrane CENTRAL Register of Controlled Trials (Ovid) from inception to November 2019. Repeated searches were performed in October 2021 to identify new publications. An information specialist and researchers developed a subject-specific MEDLINE search strategy and modified it several times to identify all relevant studies. The MEDLINE search strategy was adapted for searching all other databases.

**a. Search Strategy for from inception to November 2019**
Completed by: Jessica Babineau MLIS
Submitted: 2019.11.22

**SEARCH DETAILS (in text suggestion)**
Searches were conducted in MEDLINE(R) ALL (in Ovid, including Epub Ahead of Print, In-Process & Other Non-Indexed Citations, Ovid MEDLINE(R) Daily and then translated into Embase (Ovid), and the Cochrane CENTRAL Register of Controlled Trials (Ovid). All searches conducted in November 2019.

Search strategies included the use of text words and subject headings (e.g. MeSH, Emtree) related to (1) Central nervous system infections and (2) dementia or cognitive impairment. Searches were limited to identify either prognostic [*] or longitudinal studies, limited English language, and human adult populations when possible. Reviews, letters and conference proceedings were excluded when possible. Searches were conducted by an Information Specialist (JB).

For a more comprehensive search narrative, and Medline search strategy, see appendix (suggestion at the end of this document).

*Appendix suggestions - see page 15 of this document*

**Cite prognostic filter as: Geersing, G. J., Bouwmeester, W., Zuithoff, P., Spijker, R., Leeflang, M., & Moons, K. (2012). Search filters for finding prognostic and diagnostic prediction studies in Medline to enhance systematic reviews. PloS one, 7(2), e32844.*
*Source: https://www.ncbi.nlm.nih.gov/pmc/articles/PMC3290602/*

**PRE-DUPLICATE REMOVAL RESULTS (for PRISMA Reporting)**

*TOTAL Results: 14293 citations*
- Medline ALL (Ovid): 5636 citations
- Embase (Ovid):  8158 citations
- CENTRAL (Ovid): 499 citations

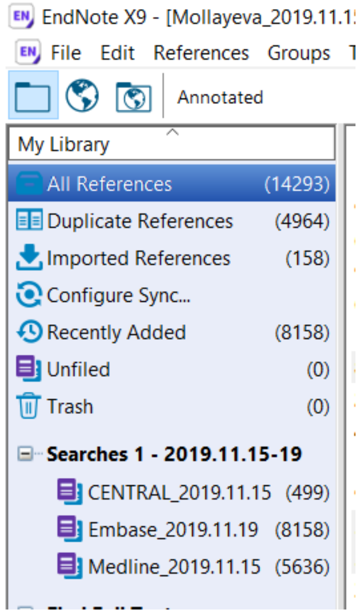


**<u>POST-DUPLICATE REMOVAL RESULTS (for PRISMA Reporting)</u>**

*TOTAL Results: 11033 citations*

- Medline ALL (Ovid): 5605 citations
- Embase (Ovid): 5213 citations
- CENTRAL (Ovid): 215 citations

Duplicates Removed: 3260

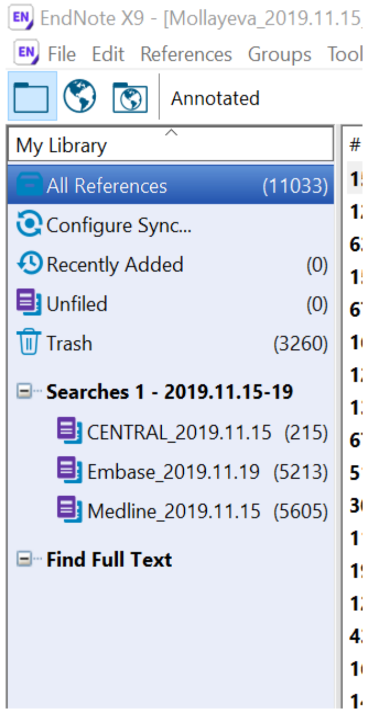


**SEARCH STRATEGIES**

Database: Ovid MEDLINE(R) ALL <1946 to November 14, 2019>
Search Strategy:

--------------------------------------------------------------------------------

1 exp Central Nervous System Infections/ (129472)
2 (central nervous system adj2 infect*).tw,kf. (3689)
3 (CNS adj2 infect*).tw,kf. (3250)
4 ((brain or cerebral or cranial or intracranial or epidural or extradural or spine or spinal or mening*) adj2 abscess*).tw,kf. (9777)
5 ((brain or cerebral or cranial or intracranial or epidural or extradural or spine or spinal or mening*) adj2 infect*).tw,kf. (10650)
6 myeloencephaliti*.tw,kf. (251)
7 encephalomyeliti*.tw,kf. (19831)
8 cerebromeningiti*.tw,kf. (11)
9 ((perimeningeal or parameningeal or encephalitis or myelitis) adj2 infect*).tw,kf. (1263)
10 ((myelitis or myelitides or myelopathies or myelopathy) adj2 (inflamma* or infect* or necrotising)).tw,kf. (200)
11 (spinal adj3 (inflamm* or infect*)).tw,kf. (2950)
12 (prion adj2 (diseas* or infect* or disorder*)).tw,kf. (7263)
13 transmissible spongiform encephalopath*.tw,kf. (2665)
14 transmissible dementia?.tw,kf. (25)
15 exp DNA Virus Infections/ (241537)
16 (dna adj2 vir* infect*).tw,kf. (407)

17 exp RNA Virus Infections/ (606174)
18 (rna adj2 vir* infect*).tw,kf. (703)
19 exp RNA Viruses/ (437239)
20 exp DNA Viruses/ (273698)
21 Virus Diseases/ (37805)
22 Influenza, Human/ (47545)
23 (flu or grippe or influenza?).tw,kf. (120955)
24 exp Measles virus/ (6388)
25 exp Measles/ (15150)
26 measles.tw,kf. (23289)
27 (edmonston adj3 virus).tw,kf. (128)
28 exp Herpes Simplex/ (23924)
29 exp Simplexvirus/ (30075)
30 Herpesviridae Infections/ (14320)
31 (herpes or herpesvirus* or herpesviridae or simplexvirus* or simplex virus* or hhv? or hsv?).tw,kf. (85925)
32 exp HIV Infections/ (275870)
33 exp HIV/ (96832)
34 HIV?.tw,kf. (306421)
35 human immunodeficiency virus*.tw,kf. (86362)
36 aids virus*.tw,kf. (1112)
37 acquired immune deficiency syndrome virus*.tw,kf. (19)
38 acquired immunodeficiency syndrome virus*.tw,kf. (33)
39 htlv-iii.tw,kf. (1659)
40 human t cell lymphotropic virus*.tw,kf. (2261)
41 human t cell leukemia virus*.tw,kf. (3223)
42 lymphadenopathy associated virus*.tw,kf. (297)
43 Herpesvirus 4, Human/ (23343)
44 burkitts lymphoma virus*.tw,kf. (2)
45 (e b virus* or eb virus* or EBV).tw,kf. (25873)
46 epstein barr virus*.tw,kf. (31430)
47 burkitt herpesvirus.tw,kf. (0)
48 infectious mononucleosis virus*.tw,kf. (7)
49 hhv-4.tw,kf. (37)
50 hhv4.tw,kf. (20)
51 exp Adenoviridae/ (35622)
52 adenovirus*.tw,kf. (44164)
53 adenoviridae?.tw,kf. (172)
54 ichtadenovirus*.tw,kf. (1)
55 atadenovirus*.tw,kf. (68)
56 adenovirus*.tw,kf. (44164)
57 apc virus*.tw,kf. (52)
58 exp Enterovirus/ (22547)

59 enterovirus infections/ or exp coxsackievirus infections/ (10544)
60 coxsackie virus*.tw,kf. (2299)
61 coxsackievirus*.tw,kf. (4695)
62 enterovirus*.tw,kf. (10280)
63 herpangina?.tw,kf. (275)
64 (epidemic adj2 (myalgia? or peurodynia?)).tw,kf. (57)
65 (hand adj1 foot adj1 mouth adj1 diseas*).tw,kf. (350)
66 exp Neisseria/ (20078)
67 neisseria?.tw,kf. (20069)
68 Pseudomonas aeruginosa/ (41455)
69 Pseudomonas Infections/ (19993)
70 pseudomonas aeruginosa.tw,kf. (55489)
71 pseudomonas pyocyanea.tw,kf. (157)
72 (pseudomonas adj2 infect*).tw,kf. (5645)
73 exp Escherichia coli/ (274218)
74 escherichia coli.tw,kf. (264514)
75 ecoli.tw,kf. (4271)
76 e? coli.tw,kf. (130994)
77 eaggec.tw,kf. (146)
78 Streptococcus pyogenes/ (13255)
79 streptococcus pyogenes.tw,kf. (7742)
80 streptococcus group a.tw,kf. (274)
81 flesh-eating bacteria.tw,kf. (31)
82 exp Legionellosis/ (5452)
83 legionellos#s.tw,kf. (1250)
84 legionella pneumophila?.tw,kf. (5512)
85 (legionnaire* adj1 disease?).tw,kf. (3195)
86 pontiac fever?.tw,kf. (145)
87 exp Yersinia/ (10681)
88 yersinia?.tw,kf. (12783)
89 Candida albicans/ (24253)
90 candida albican?.tw,kf. (31971)
91 exp Candidiasis/ (31380)
92 candidias#s.tw,kf. (15215)
93 monilias#s.tw,kf. (2057)
94 Plasmodium vivax/ (5107)
95 plasmodium vivax?.tw,kf. (5702)
96 Plasmodium falciparum/ (28766)
97 plasmodium falciparum?.tw,kf. (30402)
98 Entamoeba histolytica/ (5835)
99 entamoeba histolytica?.tw,kf. (6282)
100 exp Mycoplasma Infections/ (12163)

101 eperythrozoonos#s.tw,kf. (77)

102 contagious pleuropneumonia?.tw,kf. (50)

103 (mycoplasma adj3 (infect* or pneumonia?)).tw,kf. (8969)

104 primary atypical pneumonia?.tw,kf. (331)

105 exp Spirochaetales Infections/ (50197)

106 ((spirochete or spirochaetales) adj2 infect*).tw,kf. (154)

107 ((spirochete or spirochaetales or borrelia? or burgdorferi or leptospira or treponemal) adj2 infect*).tw,kf. (3636)

108 (lyme adj2 (diseas* or arthritis or borreliosis)).tw,kf. (10682)

109 relapsing fever?.tw,kf. (1463)

110 cane cutter fever?.tw,kf. (0)

111 leptospiros#s.tw,kf. (7666)

112 stuttgart disease?.tw,kf. (3)

113 swineherd* disease?.tw,kf. (20)

114 weil?? disease?.tw,kf. (605)

115 icterohemorrhagic leptospiros#s.tw,kf. (61)

116 spirochetal jaundice?.tw,kf. (2)

117 bejel?.tw,kf. (86)

118 pinta.tw,kf. (191)

119 syphilis.tw,kf. (28194)

120 great pox.tw,kf. (11)

121 frambesia?.tw,kf. (128)

122 yaws.tw,kf. (927)

123 Protozoan Infections/ (4634)

124 histomonias#s.tw,kf. (112)

125 protozoan infection?.tw,kf. (1191)

126 exp Helminthiasis/ (124954)

127 helminthias#s.tw,kf. (2360)

128 nematomorpha infection?.tw,kf. (2)

129 or/1-128 (2166845)

130 exp Neurocognitive Disorders/ or Cognition/ (321176)

131 exp Dementia/ (159241)

132 dement*.tw,kf. (110178)

133 alzheimer*.tw,kf. (140719)

134 (lewy* adj2 bod*).tw,kf. (8972)

135 deliri*.tw,kf. (14996)

136 (chronic adj2 cerebrovascular).tw,kf. (628)

137 (chronic adj2 cerebrovascular).tw,kf. (628)

138 ('organic brain disease' or 'organic brain syndrome').tw,kf. (795)

139 ('normal pressure hydrocephalus' and 'shunt*').tw,kf. (1219)

140 'benign senescent forgetfulness'.tw,kf. (17)

141 (cerebr* adj2 deteriorat*).tw,kf. (231)

142 (cerebral* adj2 insufficient*).tw,kf. (78)

143 (pick* adj2 disease).tw,kf. (3163)
144 (creutzfeldt or jcd or cjd).tw,kf. (6906)
145 huntington*.tw,kf. (17294)
146 korsako*.tw,kf. (1579)
147 ((cognitive or cognition or neurocognit* or mental) adj2 (disorder* or function* or dysfunction* or deteriorat* or decline? or impairment?)).tw,kf. (200807)
148 or/130-147 (522636)
149 129 and 148 (25338)
150 Validat*.mp. or Predict*.ti. or Rule*.mp. (950606)
151 (Predict* and (Outcome* or Risk* or Model*)).mp. (941688)
152 ((History or Variable* or Criteria or Scor* or Characteristic* or Finding* or Factor*) and (Predict* or Model* or Decision* or Identif* or Prognos*)).mp. (3691455)
153 Decision*.mp. and ((Model* or Clinical*).mp. or Logistic Models/) (205315)
154 (Prognostic and (History or Variable* or Criteria or Scor* or Characteristic* or Finding* or Factor* or Model*)).mp. (217987)
155 predict*.tw,kf. (1497792)
156 "Predictive Value of Tests"/ (195818)
157 scor*.tw,kf. (914845)
158 observ*.tw,kf. (3377999)
159 Observer Variation/ (41419)
160 (Stratification or Discrimination or Discriminate or "c statistic" or "Area under the curve" or AUC or Calibration or Indices or Algorithm or Multivariable).mp. (830400)
161 ROC Curve/ (54487)
162 or/150-161 (8155806)
163 exp cohort studies/ (1922372)
164 cohort?.mp. (643670)
165 Longitudinal Studies/ (128744)
166 (longitudinal* adj2 (study or studies)).mp. (173223)
167 Follow-up Studies/ (626830)
168 ((follow-up or followup) adj2 (study or studies)).mp. (654629)
169 Prospective Studies/ (520215)
170 (prospective* adj2 (study or studies)).mp. (636552)
171 Retrospective Studies/ (781261)
172 (retrospective* adj2 (study or studies)).mp. (851406)
173 Controlled Before-After Studies/ (450)
174 (cba adj1 (design? or procedure? or study or studies)).mp. (134)
175 (before-after adj2 (design? or procedure? or study or studies)).mp. (2260)
176 (before adj1 after adj2 (study or studies)).mp. (1817)
177 Interrupted Time Series Analysis/ (710)
178 (interrupt* adj1 time adj1 series).mp. (3017)
179 Cross-Over Studies/ (46492)
180 (cross over adj2 (design? or procedure? or study or studies or trial?)).mp. (55173)
181 or/163-180 (2389708)
182 162 or 181 (9222644)
183 149 and 182 (10092)
184 183 not (exp animals/ not exp humans/) (9273)

185 184 not ((exp infant/ or exp child/) not exp adult/) (8714)
186 limit 185 to (letter or "review" or "systematic review" or systematic reviews as topic) (1474)
187 185 not 186 (7240)
188 limit 187 to english language (6785)
189 limit 188 to yr="1999 -Current" (5636)

***************************

Database: Cochrane Central Register of Controlled Trials <2014 to Present>
Search Strategy:
--------------------------------------------------------------------------------
1 exp Central Nervous System Infections/ (1504)
2 (central nervous system adj2 infect*).tw,kw. (169)
3 (CNS adj2 infect*).tw,kw. (116)
4 ((brain or cerebral or cranial or intracranial or epidural or extradural or spine or spinal or mening*) adj2 abscess*).tw,kw. (95)
5 ((brain or cerebral or cranial or intracranial or epidural or extradural or spine or spinal or mening*) adj2 infect*).tw,kw. (654)
6 myeloencephaliti*.tw,kw. (1)
7 encephalomyeliti*.tw,kw. (203)
8 cerebromeningiti*.tw,kw. (0)
9 ((perimeningeal or parameningeal or encephalitis or myelitis) adj2 infect*).tw,kw. (33)
10 ((myelitis or myelitides or myelopathies or myelopathy) adj2 (inflamma* or infect* or necrotising)).tw,kw. (6)
11 (spinal adj3 (inflamm* or infect*)).tw,kw. (330)
12 (prion adj2 (diseas* or infect* or disorder*)).tw,kw. (31)
13 transmissible spongiform encephalopath*.tw,kw. (0)
14 transmissible dementia?.tw,kw. (0)
15 exp DNA Virus Infections/ (6886)
16 (dna adj2 vir* infect*).tw,kw. (9)
17 exp RNA Virus Infections/ (20616)
18 (rna adj2 vir* infect*).tw,kw. (8)
19 exp RNA Viruses/ (6970)
20 exp DNA Viruses/ (2780)
21 Virus Diseases/ (560)
22 Influenza, Human/ (2324)
23 (flu or grippe or influenza?).tw,kw. (9714)
24 exp Measles virus/ (87)
25 exp Measles/ (259)
26 measles.tw,kw. (1049)
27 (edmonston adj3 virus).tw,kw. (13)
28 exp Herpes Simplex/ (1003)
29 exp Simplexvirus/ (368)
30 Herpesviridae Infections/ (84)

31 (herpes or herpesvirus* or herpesviridae or simplexvirus* or simplex virus* or hhv? or hsv?).tw,kw. (4062)
32 exp HIV Infections/ (11918)
33 exp HIV/ (3173)
34 HIV?.tw,kw. (23816)
35 human immunodeficiency virus*.tw,kw. (10978)
36 aids virus*.tw,kw. (11)
37 acquired immune deficiency syndrome virus*.tw,kw. (0)
38 acquired immunodeficiency syndrome virus*.tw,kw. (0)
39 htlv-iii.tw,kw. (12)
40 human t cell lymphotropic virus*.tw,kw. (19)
41 human t cell leukemia virus*.tw,kw. (22)
42 lymphadenopathy associated virus*.tw,kw. (1)
43 Herpesvirus 4, Human/ (109)
44 burkitts lymphoma virus*.tw,kw. (0)
45 (e b virus* or eb virus* or EBV).tw,kw. (586)
46 epstein barr virus*.tw,kw. (431)
47 burkitt herpesvirus.tw,kw. (0)
48 infectious mononucleosis virus*.tw,kw. (0)
49 hhv-4.tw,kw. (0)
50 hhv4.tw,kw. (0)
51 exp Adenoviridae/ (194)
52 adenovirus*.tw,kw. (689)
53 adenoviridae?.tw,kw. (112)
54 ichtadenovirus*.tw,kw. (0)
55 atadenovirus*.tw,kw. (0)
56 adenovirus*.tw,kw. (689)
57 apc virus*.tw,kw. (0)
58 exp Enterovirus/ (166)
59 enterovirus infections/ or exp coxsackievirus infections/ (117)
60 coxsackie virus*.tw,kw. (12)
61 coxsackievirus*.tw,kw. (28)
62 enterovirus*.tw,kw. (199)
63 herpangina?.tw,kw. (13)
64 (epidemic adj2 (myalgia? or peurodynia?)).tw,kw. (0)
65 (hand adj1 foot adj1 mouth adj1 diseas*).tw,kw. (114)
66 exp Neisseria/ (484)
67 neisseria?.tw,kw. (1051)
68 Pseudomonas aeruginosa/ (388)
69 Pseudomonas Infections/ (482)
70 pseudomonas aeruginosa.tw,kw. (1989)
71 pseudomonas pyocyanea.tw,kw. (7)
72 (pseudomonas adj2 infect*).tw,kw. (587)

73 exp Escherichia coli/ (733)
74 escherichia coli.tw,kw. (2519)
75 ecoli.tw,kw. (125)
76 e? coli.tw,kw. (1313)
77 eaggec.tw,kw. (3)
78 Streptococcus pyogenes/ (329)
79 streptococcus pyogenes.tw,kw. (265)
80 streptococcus group a.tw,kw. (67)
81 flesh-eating bacteria.tw,kw. (0)
82 exp Legionellosis/ (21)
83 legionellos#s.tw,kw. (6)
84 legionella pneumophila?.tw,kw. (67)
85 (legionnaire* adj1 disease?).tw,kw. (24)
86 pontiac fever?.tw,kw. (0)
87 exp Yersinia/ (15)
88 yersinia?.tw,kw. (42)
89 Candida albicans/ (242)
90 candida albican?.tw,kw. (646)
91 exp Candidiasis/ (996)
92 candidias#s.tw,kw. (1803)
93 monilias#s.tw,kw. (19)
94 Plasmodium vivax/ (108)
95 plasmodium vivax?.tw,kw. (391)
96 Plasmodium falciparum/ (849)
97 plasmodium falciparum?.tw,kw. (2181)
98 Entamoeba histolytica/ (25)
99 entamoeba histolytica?.tw,kw. (92)
100 exp Mycoplasma Infections/ (123)
101 eperythrozoonos#s.tw,kw. (1)
102 contagious pleuropneumonia?.tw,kw. (1)
103 (mycoplasma adj3 (infect* or pneumonia?)).tw,kw. (306)
104 primary atypical pneumonia?.tw,kw. (3)
105 exp Spirochaetales Infections/ (332)
106 ((spirochete or spirochaetales) adj2 infect*).tw,kw. (3)
107 ((spirochete or spirochaetales or borrelia? or burgdorferi or leptospira or treponemal) adj2 infect*).tw,kw. (50)
108 (lyme adj2 (diseas* or arthritis or borreliosis)).tw,kw. (208)
109 relapsing fever?.tw,kw. (23)
110 cane cutter fever?.tw,kw. (0)
111 leptospiros#s.tw,kw. (82)
112 stuttgart disease?.tw,kw. (0)
113 swineherd* disease?.tw,kw. (0)
114 weil?? disease?.tw,kw. (1)

115 icterohemorrhagic leptospiros#s.tw,kw. (0)
116 spirochetal jaundice?.tw,kw. (0)
117 bejel?.tw,kw. (0)
118 pinta.tw,kw. (3)
119 syphilis.tw,kw. (638)
120 great pox.tw,kw. (0)
121 frambesia?.tw,kw. (0)
122 yaws.tw,kw. (23)
123 Protozoan Infections/ (15)
124 histomonias#s.tw,kw. (0)
125 protozoan infection?.tw,kw. (15)
126 exp Helminthiasis/ (1323)
127 helminthias#s.tw,kw. (212)
128 nematomorpha infection?.tw,kw. (0)
129 or/1-128 (67646)
130 exp Neurocognitive Disorders/ or Cognition/ (7837)
131 exp Dementia/ (5566)
132 dement*.tw,kw. (12297)
133 alzheimer*.tw,kw. (10706)
134 (lewy* adj2 bod*).tw,kw. (407)
135 deliri*.tw,kw. (3007)
136 (chronic adj2 cerebrovascular).tw,kw. (509)
137 (chronic adj2 cerebrovascular).tw,kw. (509)
138 ('organic brain disease' or 'organic brain syndrome').tw,kw. (149)
139 ('normal pressure hydrocephalus' and 'shunt*').tw,kw. (81)
140 'benign senescent forgetfulness'.tw,kw. (2)
141 (cerebr* adj2 deteriorat*).tw,kw. (36)
142 (cerebral* adj2 insufficient*).tw,kw. (5)
143 (pick* adj2 disease).tw,kw. (29)
144 (creutzfeldt or jcd or cjd).tw,kw. (67)
145 huntington*.tw,kw. (675)
146 korsako*.tw,kw. (81)
147 ((cognitive or cognition or neurocognit* or mental) adj2 (disorder* or function* or dysfunction* or deteriorat* or decline? or impairment?)).tw,kw. (34212)
148 or/130-147 (54268)
149 129 and 148 (937)
150 Validat*.mp. or Predict*.ti. or Rule*.mp. (63199)
151 (Predict* and (Outcome* or Risk* or Model*)).mp. (69266)
152 ((History or Variable* or Criteria or Scor* or Characteristic* or Finding* or Factor*) and (Predict* or Model* or Decision* or Identif* or Prognos*)).mp. (214061)
153 Decision*.mp. and ((Model* or Clinical*).mp. or Logistic Models/) (21933)
154 (Prognostic and (History or Variable* or Criteria or Scor* or Characteristic* or Finding* or Factor* or Model*)).mp. (16178)
155 predict*.tw,kw. (96911)
156 "Predictive Value of Tests"/ (7627)

157 scor*.tw,kw. (247174)
158 observ*.tw,kw. (263600)
159 Observer Variation/ (2328)
160 (Stratification or Discrimination or Discriminate or "c statistic" or "Area under the curve" or AUC or Calibration or Indices or Algorithm or Multivariable).mp. (78941)
161 ROC Curve/ (1284)
162 or/150-161 (660363)
163 exp cohort studies/ (157379)
164 cohort?.mp. (60513)
165 Longitudinal Studies/ (6637)
166 (longitudinal* adj2 (study or studies)).mp. (12320)
167 Follow-up Studies/ (62517)
168 ((follow-up or followup) adj2 (study or studies)).mp. (83662)
169 Prospective Studies/ (97111)
170 (prospective* adj2 (study or studies)).mp. (172664)
171 Retrospective Studies/ (8790)
172 (retrospective* adj2 (study or studies)).mp. (25229)
173 Controlled Before-After Studies/ (18)
174 (cba adj1 (design? or procedure? or study or studies)).mp. (24)
175 (before-after adj2 (design? or procedure? or study or studies)).mp. (1233978)
176 (before adj1 after adj2 (study or studies)).mp. (1198127)
177 Interrupted Time Series Analysis/ (11)
178 (interrupt* adj1 time adj1 series).mp. (317)
179 Cross-Over Studies/ (39244)
180 (cross over adj2 (design? or procedure? or study or studies or trial?)).mp. (56920)
181 or/163-180 (1237560)
182 162 or 181 (1325240)
183 149 and 182 (865)
184 183 not (exp animals/ not exp humans/) (865)
185 184 not ((exp infant/ or exp child/) not exp adult/) (818)
186 limit 185 to (letter or "review" or "review literature" or review, academic or review, tutorial) (3)
187 185 not 186 (815)
188 limit 187 to english language (543)
189 limit 188 to yr="1999 -Current" (499)

***************************

Database: Embase <1974 to 2019 November 15>
Search Strategy:
--------------------------------------------------------------------------------
1 exp Central Nervous System Infection/ (167050)
2 (central nervous system adj2 infect*).tw,kw. (4756)
3 (CNS adj2 infect*).tw,kw. (5016)

4 ((brain or cerebral or cranial or intracranial or epidural or extradural or spine or spinal or mening*) adj2 abscess*).tw,kw. (10914)
5 ((brain or cerebral or cranial or intracranial or epidural or extradural or spine or spinal or mening*) adj2 infect*).tw,kw. (13964)
6 myeloencephaliti*.tw,kw. (269)
7 encephalomyeliti*.tw,kw. (23266)
8 cerebromeningiti*.tw,kw. (12)
9 ((perimeningeal or parameningeal or encephalitis or myelitis) adj2 infect*).tw,kw. (1511)
10 ((myelitis or myelitides or myelopathies or myelopathy) adj2 (inflamma* or infect* or necrotising)).tw,kw. (305)
11 (spinal adj3 (inflamm* or infect*)).tw,kw. (4235)
12 (prion adj2 (diseas* or infect* or disorder*)).tw,kw. (10004)
13 transmissible spongiform encephalopath*.tw,kw. (3371)
14 transmissible dementia?.tw,kw. (38)
15 exp DNA Virus Infection/ (290285)
16 (dna adj2 vir* infect*).tw,kw. (458)
17 exp RNA Virus Infection/ (703073)
18 (rna adj2 vir* infect*).tw,kw. (778)
19 exp RNA Virus/ (265070)
20 exp DNA Virus/ (239739)
21 Virus infection/ (157763)
22 exp Influenza/ (82286)
23 (flu or grippe or influenza?).tw,kw. (138633)
24 exp Measles virus/ (8343)
25 exp Measles/ (17417)
26 measles.tw,kw. (22991)
27 (edmonston adj3 virus).tw,kw. (131)
28 exp Herpes Simplex/ (31737)
29 Simplexvirus/ or exp Herpes simplex virus/ (33966)
30 Herpesviridae Infections/ (6456)
31 (herpes or herpesvirus* or herpesviridae or simplexvirus* or simplex virus* or hhv? or hsv?).tw,kw. (101414)
32 exp Human immunodeficiency virus infection/ (369646)
33 exp Human immunodeficiency virus/ (190545)
34 HIV?.tw,kw. (391799)
35 human immunodeficiency virus*.tw,kw. (96187)
36 aids virus*.tw,kw. (1131)
37 acquired immune deficiency syndrome virus*.tw,kw. (18)
38 acquired immunodeficiency syndrome virus*.tw,kw. (30)
39 htlv-iii.tw,kw. (1857)
40 human t cell lymphotropic virus*.tw,kw. (2621)
41 human t cell leukemia virus*.tw,kw. (3650)
42 lymphadenopathy associated virus*.tw,kw. (299)
43 Epstein Barr virus/ (39231)
44 burkitts lymphoma virus*.tw,kw. (0)
45 (e b virus* or eb virus* or EBV).tw,kw. (36830)

46 epstein barr virus*.tw,kw. (37751)
47 burkitt herpesvirus.tw,kw. (0)
48 infectious mononucleosis virus*.tw,kw. (4)
49 hhv-4.tw,kw. (62)
50 hhv4.tw,kw. (32)
51 exp Adenoviridae/ (9603)
52 adenovirus*.tw,kw. (53032)
53 adenoviridae?.tw,kw. (316)
54 ichtadenovirus*.tw,kw. (3)
55 atadenovirus*.tw,kw. (81)
56 adenovirus*.tw,kw. (53032)
57 apc virus*.tw,kw. (1)
58 exp Enterovirus/ (18857)
59 enterovirus infections/ or exp coxsackie virus infections/ (2497)
60 coxsackie virus*.tw,kw. (1310)
61 coxsackievirus*.tw,kw. (5375)
62 enterovirus*.tw,kw. (11871)
63 herpangina?.tw,kw. (240)
64 (epidemic adj2 (myalgia? or peurodynia?)).tw,kw. (23)
65 (hand adj1 foot adj1 mouth adj1 diseas*).tw,kw. (469)
66 exp Neisseria/ (31484)
67 neisseria?.tw,kw. (22841)
68 exp Pseudomonas aeruginosa/ (93310)
69 Pseudomonas Infections/ (2803)
70 pseudomonas aeruginosa.tw,kw. (69763)
71 pseudomonas pyocyanea.tw,kw. (33)
72 (pseudomonas adj2 infect*).tw,kw. (6609)
73 exp Escherichia coli/ (350101)
74 escherichia coli.tw,kw. (268789)
75 ecoli.tw,kw. (9198)
76 e? coli.tw,kw. (150618)
77 eaggec.tw,kw. (159)
78 Streptococcus pyogenes/ (14723)
79 streptococcus pyogenes.tw,kw. (9187)
80 streptococcus group a.tw,kw. (335)
81 flesh-eating bacteria.tw,kw. (45)
82 legionnaire disease/ (5957)
83 legionellos#s.tw,kw. (1439)
84 legionella pneumophila?.tw,kw. (6285)
85 (legionnaire* adj1 disease?).tw,kw. (3683)
86 pontiac fever?.tw,kw. (170)
87 exp Yersinia/ (15547)

88 yersinia?.tw,kw. (13968)
89 Candida albicans/ (53384)
90 candida albican?.tw,kw. (39804)
91 exp Candidiasis/ (46862)
92 candidias#s.tw,kw. (19849)
93 monilias#s.tw,kw. (249)
94 Plasmodium vivax/ (8162)
95 plasmodium vivax?.tw,kw. (6798)
96 exp Plasmodium falciparum/ (39514)
97 plasmodium falciparum?.tw,kw. (36295)
98 Entamoeba histolytica/ (8509)
99 entamoeba histolytica?.tw,kw. (6585)
100 exp Mycoplasma Infections/ (6892)
101 eperythrozoonos#s.tw,kw. (59)
102 contagious pleuropneumonia?.tw,kw. (46)
103 (mycoplasma adj3 (infect* or pneumonia?)).tw,kw. (10327)
104 primary atypical pneumonia?.tw,kw. (84)
105 exp Spirochaetales Infections/ (54728)
106 ((spirochete or spirochaetales) adj2 infect*).tw,kw. (164)
107 ((spirochete or spirochaetales or borrelia? or burgdorferi or leptospira or treponemal) adj2 infect*).tw,kw. (3807)
108 (lyme adj2 (diseas* or arthritis or borreliosis)).tw,kw. (12894)
109 relapsing fever?.tw,kw. (1197)
110 cane cutter fever?.tw,kw. (0)
111 leptospiros#s.tw,kw. (6739)
112 stuttgart disease?.tw,kw. (2)
113 swineherd* disease?.tw,kw. (2)
114 weil?? disease?.tw,kw. (332)
115 icterohemorrhagic leptospiros#s.tw,kw. (41)
116 spirochetal jaundice?.tw,kw. (1)
117 bejel?.tw,kw. (72)
118 pinta.tw,kw. (109)
119 syphilis.tw,kw. (22828)
120 great pox.tw,kw. (10)
121 frambesia?.tw,kw. (13)
122 yaws.tw,kw. (545)
123 Protozoan Infections/ (4379)
124 histomonias#s.tw,kw. (82)
125 protozoan infection?.tw,kw. (957)
126 exp Helminthiasis/ (104055)
127 helminthias#s.tw,kw. (2125)
128 nematomorpha infection?.tw,kw. (3)
129 or/1-128 (2472509)

| | |
|---|---|
| 130 | exp Neurocognitive Disorders/ or Cognition/ (922749) |
| 131 | exp Dementia/ (346997) |
| 132 | dement*.tw,kw. (164702) |
| 133 | alzheimer*.tw,kw. (200422) |
| 134 | (lewy* adj2 bod*).tw,kw. (14100) |
| 135 | deliri*.tw,kw. (23168) |
| 136 | (chronic adj2 cerebrovascular).tw,kw. (1002) |
| 137 | (chronic adj2 cerebrovascular).tw,kw. (1002) |
| 138 | ('organic brain disease' or 'organic brain syndrome').tw,kw. (1033) |
| 139 | ('normal pressure hydrocephalus' and 'shunt*').tw,kw. (1761) |
| 140 | 'benign senescent forgetfulness'.tw,kw. (30) |
| 141 | (cerebr* adj2 deteriorat*).tw,kw. (322) |
| 142 | (cerebral* adj2 insufficient*).tw,kw. (101) |
| 143 | (pick* adj2 disease).tw,kw. (3978) |
| 144 | (creutzfeldt or jcd or cjd).tw,kw. (9189) |
| 145 | huntington*.tw,kw. (23380) |
| 146 | korsako*.tw,kw. (1744) |
| 147 | ((cognitive or cognition or neurocognit* or mental) adj2 (disorder* or function* or dysfunction* or deteriorat* or decline? or impairment?)).tw,kw. (286494) |
| 148 | or/130-147 (1064443) |
| 149 | 129 and 148 (56574) |
| 150 | Validat*.mp. or Predict*.ti. or Rule*.mp. (1355732) |
| 151 | (Predict* and (Outcome* or Risk* or Model*)).mp. (1286885) |
| 152 | ((History or Variable* or Criteria or Scor* or Characteristic* or Finding* or Factor*) and (Predict* or Model* or Decision* or Identif* or Prognos*)).mp. (4887209) |
| 153 | Decision*.mp. and ((Model* or Clinical*).mp. or statistical model/) (399841) |
| 154 | (Prognostic and (History or Variable* or Criteria or Scor* or Characteristic* or Finding* or Factor* or Model*)).mp. (345199) |
| 155 | predict*.tw,kw. (2018213) |
| 156 | exp predictive value/ (159845) |
| 157 | scor*.tw,kw. (1439013) |
| 158 | observ*.tw,kw. (4265594) |
| 159 | Observer Variation/ (19757) |
| 160 | (Stratification or Discrimination or Discriminate or "c statistic" or "Area under the curve" or AUC or Calibration or Indices or Algorithm or Multivariable).mp. (1283692) |
| 161 | receiver operating characteristic/ (114445) |
| 162 | or/150-161 (10647841) |
| 163 | cohort analysis/ (529450) |
| 164 | cohort?.mp. (1035191) |
| 165 | exp Longitudinal Study/ (133639) |
| 166 | (longitudinal* adj2 (study or studies)).mp. (182723) |
| 167 | Follow-up/ (1474745) |
| 168 | ((follow-up or followup) adj2 (study or studies)).mp. (80506) |
| 169 | Prospective Study/ (566158) |
| 170 | (prospective* adj2 (study or studies)).mp. (747022) |
| 171 | Retrospective Study/ (851053) |

172 (retrospective* adj2 (study or studies)).mp. (947032)
173 (cba adj1 (design? or procedure? or study or studies)).mp. (163)
174 (before-after adj2 (design? or procedure? or study or studies)).mp. (2580)
175 (before adj1 after adj2 (study or studies)).mp. (2025)
176 Interrupted Time Series Analysis/ (187510)
177 (interrupt* adj1 time adj1 series).mp. (3550)
178 Crossover procedure/ (61385)
179 (cross over adj2 (design? or procedure? or study or studies or trial?)).mp. (22808)
180 or/163-179 (3650198)
181 162 or 180 (12241463)
182 149 and 181 (24439)
183 182 not ((exp animals/ or exp animal experimentation/ or nonhuman/) not exp human/) (20548)
184 183 not ((exp embryo/ or exp fetus/ or exp juvenile/) not exp adult/) (18332)
185 limit 184 to (conference abstract or conference paper or "conference review") (4791)
186 184 not 185 (13541)
187 limit 186 to (books or chapter or letter or "review") (3170)
188 186 not 187 (10371)
189 188 not medline.cr. (9389)
190 limit 189 to english language (8992)
191 limit 190 to yr="1999 -Current" (8158)

****************************

## APPENDIX SUGGESTION

**Search Narrative**

We first developed the Medline strategy, which was then translated to other databases. It uses a number of concepts:
Concept A: lines 1 to 129 = Central nervous system infections (both broadly and specific infections)
Concept B: lines 130 to 148 = Cognitive impairment, or dementia
Filter C: lines 150 to 162 = Prognostic studies
Filter D: lines 163 to 180 = Longitudinal studies

The search conducted is: (A AND B) AND (C OR D)

This strategy was used in all searched databases. Searches were also limited to adult human populations, and English language when possible.

Additional limits and search fields have been used when applicable such as to exclude conference proceedings, reviews and letters.

**Medline (Ovid) Search Strategy**

1 exp Central Nervous System Infections/
2 (central nervous system adj2 infect*).tw,kf.

3 (CNS adj2 infect*).tw,kf.
4 ((brain or cerebral or cranial or intracranial or epidural or extradural or spine or spinal or mening*) adj2 abscess*).tw,kf.
5 ((brain or cerebral or cranial or intracranial or epidural or extradural or spine or spinal or mening*) adj2 infect*).tw,kf.
6 myeloencephaliti*.tw,kf.
7 encephalomyeliti*.tw,kf.
8 cerebromeningiti*.tw,kf.
9 ((perimeningeal or parameningeal or encephalitis or myelitis) adj2 infect*).tw,kf. (1263)
10 ((myelitis or myelitides or myelopathies or myelopathy) adj2 (inflamma* or infect* or necrotising)).tw,kf.
11 (spinal adj3 (inflamm* or infect*)).tw,kf.
12 (prion adj2 (diseas* or infect* or disorder*)).tw,kf.
13 transmissible spongiform encephalopath*.tw,kf.
14 transmissible dementia?.tw,kf.
15 exp DNA Virus Infections/
16 (dna adj2 vir* infect*).tw,kf.
17 exp RNA Virus Infections/
18 (rna adj2 vir* infect*).tw,kf.
19 exp RNA Viruses/
20 exp DNA Viruses/
21 Virus Diseases/
22 Influenza, Human/
23 (flu or grippe or influenza?).tw,kf.
24 exp Measles virus/
25 exp Measles/
26 measles.tw,kf.
27 (edmonston adj3 virus).tw,kf.
28 exp Herpes Simplex/
29 exp Simplexvirus/
30 Herpesviridae Infections/
31 (herpes or herpesvirus* or herpesviridae or simplexvirus* or simplex virus* or hhv? or hsv?).tw,kf.
32 exp HIV Infections/
33 exp HIV/
34 HIV?.tw,kf.
35 human immunodeficiency virus*.tw,kf.
36 aids virus*.tw,kf.
37 acquired immune deficiency syndrome virus*.tw,kf.
38 acquired immunodeficiency syndrome virus*.tw,kf.
39 htlv-iii.tw,kf.
40 human t cell lymphotropic virus*.tw,kf.
41 human t cell leukemia virus*.tw,kf.
42 lymphadenopathy associated virus*.tw,kf.
43 Herpesvirus 4, Human/
44 burkitts lymphoma virus*.tw,kf.

45 (e b virus* or eb virus* or EBV).tw,kf.
46 epstein barr virus*.tw,kf.
47 burkitt herpesvirus.tw,kf.
48 infectious mononucleosis virus*.tw,kf.
49 hhv-4.tw,kf.
50 hhv4.tw,kf.
51 exp Adenoviridae/
52 adenovirus*.tw,kf.
53 adenoviridae?.tw,kf.
54 ichtadenovirus*.tw,kf.
55 atadenovirus*.tw,kf.
56 adenovirus*.tw,kf.
57 apc virus*.tw,kf.
58 exp Enterovirus/
59 enterovirus infections/ or exp coxsackievirus infections/
60 coxsackie virus*.tw,kf.
61 coxsackievirus*.tw,kf.
62 enterovirus*.tw,kf.
63 herpangina?.tw,kf.
64 (epidemic adj2 (myalgia? or peurodynia?)).tw,kf.
65 (hand adj1 foot adj1 mouth adj1 diseas*).tw,kf.
66 exp Neisseria/
67 neisseria?.tw,kf.
68 Pseudomonas aeruginosa/
69 Pseudomonas Infections/
70 pseudomonas aeruginosa.tw,kf.
71 pseudomonas pyocyanea.tw,kf.
72 (pseudomonas adj2 infect*).tw,kf.
73 exp Escherichia coli/
74 escherichia coli.tw,kf.
75 ecoli.tw,kf.
76 e? coli.tw,kf.
77 eaggec.tw,kf.
78 Streptococcus pyogenes/
79 streptococcus pyogenes.tw,kf.
80 streptococcus group a.tw,kf.
81 flesh-eating bacteria.tw,kf.
82 exp Legionellosis/
83 legionellos#s.tw,kf.
84 legionella pneumophila?.tw,kf.
85 (legionnaire* adj1 disease?).tw,kf.
86 pontiac fever?.tw,kf.

87 exp Yersinia/
88 yersinia?.tw,kf.
89 Candida albicans/
90 candida albican?.tw,kf.
91 exp Candidiasis/
92 candidias#s.tw,kf.
93 monilias#s.tw,kf.
94 Plasmodium vivax/
95 plasmodium vivax?.tw,kf.
96 Plasmodium falciparum/
97 plasmodium falciparum?.tw,kf.
98 Entamoeba histolytica/
99 entamoeba histolytica?.tw,kf.
100 exp Mycoplasma Infections/
101 eperythrozoonos#s.tw,kf.
102 contagious pleuropneumonia?.tw,kf.
103 (mycoplasma adj3 (infect* or pneumonia?)).tw,kf.
104 primary atypical pneumonia?.tw,kf.
105 exp Spirochaetales Infections/
106 ((spirochete or spirochaetales) adj2 infect*).tw,kf.
107 ((spirochete or spirochaetales or borrelia? or burgdorferi or leptospira or treponemal) adj2 infect*).tw,kf.
108 (lyme adj2 (diseas* or arthritis or borreliosis)).tw,kf.
109 relapsing fever?.tw,kf.
110 cane cutter fever?.tw,kf.
111 leptospiros#s.tw,kf.
112 stuttgart disease?.tw,kf.
113 swineherd* disease?.tw,kf.
114 weil?? disease?.tw,kf.
115 icterohemorrhagic leptospiros#s.tw,kf.
116 spirochetal jaundice?.tw,kf.
117 bejel?.tw,kf.
118 pinta.tw,kf.
119 syphilis.tw,kf.
120 great pox.tw,kf.
121 frambesia?.tw,kf.
122 yaws.tw,kf.
123 Protozoan Infections/
124 histomonias#s.tw,kf.
125 protozoan infection?.tw,kf.
126 exp Helminthiasis/
127 helminthias#s.tw,kf.
128 nematomorpha infection?.tw,kf.

| | |
|---|---|
| 129 | or/1-128 |
| 130 | exp Neurocognitive Disorders/ or Cognition/ |
| 131 | exp Dementia/ |
| 132 | dement*.tw,kf. |
| 133 | alzheimer*.tw,kf. |
| 134 | (lewy* adj2 bod*).tw,kf. |
| 135 | deliri*.tw,kf. |
| 136 | (chronic adj2 cerebrovascular).tw,kf. |
| 137 | (chronic adj2 cerebrovascular).tw,kf. |
| 138 | ('organic brain disease' or 'organic brain syndrome').tw,kf. |
| 139 | ('normal pressure hydrocephalus' and 'shunt*').tw,kf. |
| 140 | 'benign senescent forgetfulness'.tw,kf. |
| 141 | (cerebr* adj2 deteriorat*).tw,kf. |
| 142 | (cerebral* adj2 insufficient*).tw,kf. |
| 143 | (pick* adj2 disease).tw,kf. |
| 144 | (creutzfeldt or jcd or cjd).tw,kf. |
| 145 | huntington*.tw,kf. |
| 146 | korsako*.tw,kf. |
| 147 | ((cognitive or cognition or neurocognit* or mental) adj2 (disorder* or function* or dysfunction* or deteriorat* or decline? or impairment?)).tw,kf. |
| 148 | or/130-147 |
| 149 | 129 and 148 |
| 150 | Validat*.mp. or Predict*.ti. or Rule*.mp. |
| 151 | (Predict* and (Outcome* or Risk* or Model*)).mp. |
| 152 | ((History or Variable* or Criteria or Scor* or Characteristic* or Finding* or Factor*) and (Predict* or Model* or Decision* or Identif* or Prognos*)).mp. |
| 153 | Decision*.mp. and ((Model* or Clinical*).mp. or Logistic Models/) |
| 154 | (Prognostic and (History or Variable* or Criteria or Scor* or Characteristic* or Finding* or Factor* or Model*)).mp. |
| 155 | predict*.tw,kf. |
| 156 | "Predictive Value of Tests"/ |
| 157 | scor*.tw,kf. |
| 158 | observ*.tw,kf. |
| 159 | Observer Variation/ |
| 160 | (Stratification or Discrimination or Discriminate or "c statistic" or "Area under the curve" or AUC or Calibration or Indices or Algorithm or Multivariable).mp. |
| 161 | ROC Curve/ |
| 162 | or/150-161 |
| 163 | exp cohort studies/ |
| 164 | cohort?.mp. |
| 165 | Longitudinal Studies/ |
| 166 | (longitudinal* adj2 (study or studies)).mp. |
| 167 | Follow-up Studies/ |
| 168 | ((follow-up or followup) adj2 (study or studies)).mp. |
| 169 | Prospective Studies/ |
| 170 | (prospective* adj2 (study or studies)).mp. |

171 Retrospective Studies/
172 (retrospective* adj2 (study or studies)).mp.
173 Controlled Before-After Studies/
174 (cba adj1 (design? or procedure? or study or studies)).mp.
175 (before-after adj2 (design? or procedure? or study or studies)).mp.
176 (before adj1 after adj2 (study or studies)).mp.
177 Interrupted Time Series Analysis/
178 (interrupt* adj1 time adj1 series).mp.
179 Cross-Over Studies/
180 (cross over adj2 (design? or procedure? or study or studies or trial?)).mp.
181 or/163-180
182 162 or 181
183 149 and 182
184 183 not (exp animals/ not exp humans/)
185 184 not ((exp infant/ or exp child/) not exp adult/)
186 limit 185 to (letter or "review" or "systematic review" or systematic reviews as topic)
187 185 not 186
188 limit 187 to english language
189 limit 188 to yr="1999 -Current"

**b. Search Strategy for updated searches for October 2021**
Completed by: Jessica Babineau MLIS
Submitted: 2021-10-04

**SEARCH DETAILS (in text suggestion)**
Searches were conducted in MEDLINE(R) ALL (in Ovid, including Epub Ahead of Print, In-Process & Other Non-Indexed Citations, Ovid MEDLINE(R) Daily and then translated into Embase (Ovid), and the Cochrane CENTRAL Register of Controlled Trials (Ovid). All searches conducted in November 2019 and updated October 2021.

Search strategies included the use of text words and subject headings (e.g. MeSH, Emtree) related to (1) Central nervous system infections and (2) dementia or cognitive impairment. Searches were limited to identify either prognostic [*] or longitudinal studies, limited English language, and human adult populations when possible. Reviews, letters and conference proceedings were excluded when possible. Searches were conducted by an Information Specialist (JB).

**Cite prognostic filter as: Geersing, G. J., Bouwmeester, W., Zuithoff, P., Spijker, R., Leeflang, M., & Moons, K. (2012). Search filters for finding prognostic and diagnostic prediction studies in Medline to enhance systematic reviews. PloS one, 7(2), e32844.*
*Source: https://www.ncbi.nlm.nih.gov/pmc/articles/PMC3290602/*

**2019-11 PRE-DUPLICATE REMOVAL RESULTS (for PRISMA Reporting)**

*TOTAL Results: 14293 citations*
- Medline ALL (Ovid): 5636 citations

- Embase (Ovid):  8158 citations
- CENTRAL (Ovid): 499 citations

**2021-10 PRE-DUPLICATE REMOVAL RESULTS (for PRISMA Reporting)**

*TOTAL Results:* ***18699 citations*** *(exported 5260 results)*

- Medline ALL (Ovid): 7192 citations (exported 1916 results)
- Embase (Ovid):  10912 citations (exported 3027 results)
- CENTRAL (Ovid): 595 citations (exported 317 results)

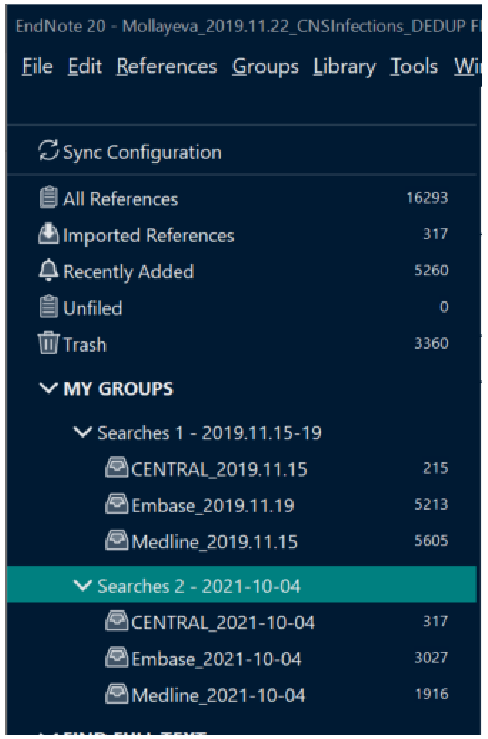


**POST-DUPLICATE REMOVAL RESULTS (for PRISMA Reporting) - TOTALS SCREENED AT TI/AB**

*TOTAL Results:  14,820 citations (11,033 OLD + 3,787 NEW)*

- Medline ALL (Ovid): 14,820 (5605 citations OLD + 786 NEW)
- Embase (Ovid): 6,391 (5213  citations + 2,950 NEW)
- CENTRAL (Ovid): 1001 (215 citations + 786 NEW)

2019 Duplicates Removed: 3260
2021 Duplicates Removed: 1,473

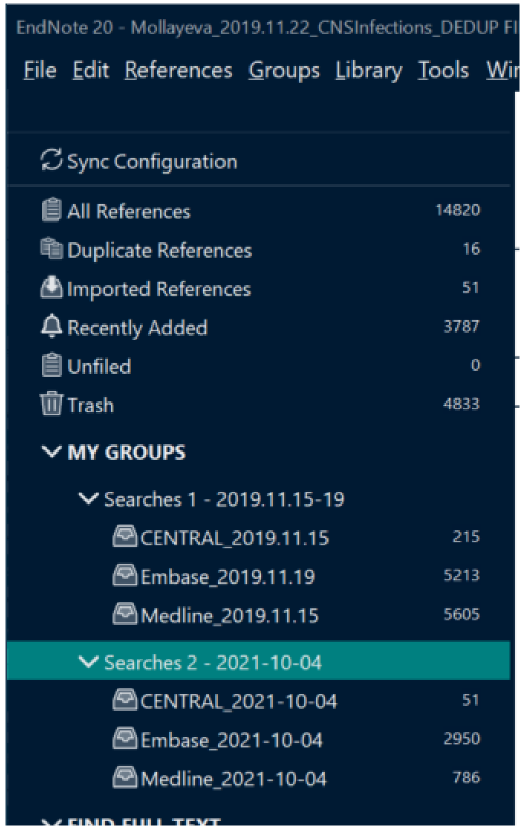


**SEARCH STRATEGIES**

Database: Ovid MEDLINE(R) ALL <1946 to October 01, 2021>
Search Strategy:
--------------------------------------------------------------------------------
1 exp Central Nervous System Infections/ (135787)
2 (central nervous system adj2 infect*).tw,kf. (4254)
3 (CNS adj2 infect*).tw,kf. (3716)
4 ((brain or cerebral or cranial or intracranial or epidural or extradural or spine or spinal or mening*) adj2 abscess*).tw,kf. (10558)
5 ((brain or cerebral or cranial or intracranial or epidural or extradural or spine or spinal or mening*) adj2 infect*).tw,kf. (11896)
6 myeloencephaliti*.tw,kf. (263)
7 encephalomyeliti*.tw,kf. (21633)

8 cerebromeningiti*.tw,kf. (12)
9 ((perimeningeal or parameningeal or encephalitis or myelitis) adj2 infect*).tw,kf. (1422)
10 ((myelitis or myelitides or myelopathies or myelopathy) adj2 (inflamma* or infect* or necrotising)).tw,kf. (238)
11 (spinal adj3 (inflamm* or infect*)).tw,kf. (3408)
12 (prion adj2 (diseas* or infect* or disorder*)).tw,kf. (7899)
13 transmissible spongiform encephalopath*.tw,kf. (2783)
14 transmissible dementia?.tw,kf. (25)
15 exp DNA Virus Infections/ (259778)
16 (dna adj2 vir* infect*).tw,kf. (475)
17 exp RNA Virus Infections/ (762050)
18 (rna adj2 vir* infect*).tw,kf. (856)
19 exp RNA Viruses/ (557819)
20 exp DNA Viruses/ (291364)
21 Virus Diseases/ (39947)
22 Influenza, Human/ (52986)
23 (flu or grippe or influenza?).tw,kf. (134814)
24 exp Measles virus/ (6623)
25 exp Measles/ (16142)
26 measles.tw,kf. (25015)
27 (edmonston adj3 virus).tw,kf. (131)
28 exp Herpes Simplex/ (24810)
29 exp Simplexvirus/ (31236)
30 Herpesviridae Infections/ (14967)
31 (herpes or herpesvirus* or herpesviridae or simplexvirus* or simplex virus* or hhv? or hsv?).tw,kf. (91760)
32 exp HIV Infections/ (296739)
33 exp HIV/ (102966)
34 HIV?.tw,kf. (333379)
35 human immunodeficiency virus*.tw,kf. (92439)
36 aids virus*.tw,kf. (1126)
37 acquired immune deficiency syndrome virus*.tw,kf. (19)
38 acquired immunodeficiency syndrome virus*.tw,kf. (33)
39 htlv-iii.tw,kf. (1659)
40 human t cell lymphotropic virus*.tw,kf. (2378)
41 human t cell leukemia virus*.tw,kf. (3423)
42 lymphadenopathy associated virus*.tw,kf. (297)
43 Herpesvirus 4, Human/ (24809)
44 burkitts lymphoma virus*.tw,kf. (2)
45 (e b virus* or eb virus* or EBV).tw,kf. (28309)
46 epstein barr virus*.tw,kf. (34034)
47 burkitt herpesvirus.tw,kf. (0)
48 infectious mononucleosis virus*.tw,kf. (7)
49 hhv-4.tw,kf. (47)

50 hhv4.tw,kf. (23)
51 exp Adenoviridae/ (36737)
52 adenovirus*.tw,kf. (47006)
53 adenoviridae?.tw,kf. (197)
54 ichtadenovirus*.tw,kf. (2)
55 atadenovirus*.tw,kf. (80)
56 adenovirus*.tw,kf. (47006)
57 apc virus*.tw,kf. (52)
58 exp Enterovirus/ (23759)
59 enterovirus infections/ or exp coxsackievirus infections/ (11454)
60 coxsackie virus*.tw,kf. (2350)
61 coxsackievirus*.tw,kf. (5151)
62 enterovirus*.tw,kf. (11498)
63 herpangina?.tw,kf. (300)
64 (epidemic adj2 (myalgia? or peurodynia?)).tw,kf. (61)
65 (hand adj1 foot adj1 mouth adj1 diseas*).tw,kf. (410)
66 exp Neisseria/ (21283)
67 neisseria?.tw,kf. (21608)
68 Pseudomonas aeruginosa/ (45310)
69 Pseudomonas Infections/ (21370)
70 pseudomonas aeruginosa.tw,kf. (62063)
71 pseudomonas pyocyanea.tw,kf. (157)
72 (pseudomonas adj2 infect*).tw,kf. (6139)
73 exp Escherichia coli/ (289644)
74 escherichia coli.tw,kf. (282855)
75 ecoli.tw,kf. (4699)
76 e? coli.tw,kf. (143429)
77 eaggec.tw,kf. (148)
78 Streptococcus pyogenes/ (13898)
79 streptococcus pyogenes.tw,kf. (8435)
80 streptococcus group a.tw,kf. (283)
81 flesh-eating bacteria.tw,kf. (32)
82 exp Legionellosis/ (5669)
83 legionellos#s.tw,kf. (1316)
84 legionella pneumophila?.tw,kf. (5867)
85 (legionnaire* adj1 disease?).tw,kf. (3381)
86 pontiac fever?.tw,kf. (158)
87 exp Yersinia/ (11100)
88 yersinia?.tw,kf. (13429)
89 Candida albicans/ (26171)
90 candida albican?.tw,kf. (35133)
91 exp Candidiasis/ (33006)

92 candidias#s.tw,kf. (16631)
93 monilias#s.tw,kf. (2058)
94 Plasmodium vivax/ (5569)
95 plasmodium vivax?.tw,kf. (6373)
96 Plasmodium falciparum/ (31074)
97 plasmodium falciparum?.tw,kf. (33077)
98 Entamoeba histolytica/ (6057)
99 entamoeba histolytica?.tw,kf. (6646)
100 exp Mycoplasma Infections/ (12923)
101 eperythrozoonos#s.tw,kf. (78)
102 contagious pleuropneumonia?.tw,kf. (53)
103 (mycoplasma adj3 (infect* or pneumonia?)).tw,kf. (9778)
104 primary atypical pneumonia?.tw,kf. (348)
105 exp Spirochaetales Infections/ (52643)
106 ((spirochete or spirochaetales) adj2 infect*).tw,kf. (161)
107 ((spirochete or spirochaetales or borrelia? or burgdorferi or leptospira or treponemal) adj2 infect*).tw,kf. (3956)
108 (lyme adj2 (diseas* or arthritis or borreliosis)).tw,kf. (11564)
109 relapsing fever?.tw,kf. (1590)
110 cane cutter fever?.tw,kf. (0)
111 leptospiros#s.tw,kf. (8365)
112 stuttgart disease?.tw,kf. (3)
113 swineherd* disease?.tw,kf. (19)
114 weil?? disease?.tw,kf. (642)
115 icterohemorrhagic leptospiros#s.tw,kf. (63)
116 spirochetal jaundice?.tw,kf. (2)
117 bejel?.tw,kf. (93)
118 pinta.tw,kf. (194)
119 syphilis.tw,kf. (29918)
120 great pox.tw,kf. (11)
121 frambesia?.tw,kf. (127)
122 yaws.tw,kf. (976)
123 Protozoan Infections/ (4745)
124 histomonias#s.tw,kf. (120)
125 protozoan infection?.tw,kf. (1299)
126 exp Helminthiasis/ (130546)
127 helminthias#s.tw,kf. (2562)
128 nematomorpha infection?.tw,kf. (2)
129 or/1-128 (2429878)
130 exp Neurocognitive Disorders/ or Cognition/ (366083)
131 exp Dementia/ (180874)
132 dement*.tw,kf. (128532)
133 alzheimer*.tw,kf. (164122)

134 (lewy* adj2 bod*).tw,kf. (10393)
135 deliri*.tw,kf. (18180)
136 (chronic adj2 cerebrovascular).tw,kf. (712)
137 (chronic adj2 cerebrovascular).tw,kf. (712)
138 ('organic brain disease' or 'organic brain syndrome').tw,kf. (798)
139 ('normal pressure hydrocephalus' and 'shunt*').tw,kf. (1399)
140 'benign senescent forgetfulness'.tw,kf. (18)
141 (cerebr* adj2 deteriorat*).tw,kf. (245)
142 (cerebral* adj2 insufficient*).tw,kf. (84)
143 (pick* adj2 disease).tw,kf. (3441)
144 (creutzfeldt or jcd or cjd).tw,kf. (7328)
145 huntington*.tw,kf. (19204)
146 korsako*.tw,kf. (1670)
147 ((cognitive or cognition or neurocognit* or mental) adj2 (disorder* or function* or dysfunction* or deteriorat* or decline? or impairment?)).tw,kf. (240651)
148 or/130-147 (601581)
149 129 and 148 (29250)
150 Validat*.mp. or Predict*.ti. or Rule*.mp. (1142274)
151 (Predict* and (Outcome* or Risk* or Model*)).mp. (1124190)
152 ((History or Variable* or Criteria or Scor* or Characteristic* or Finding* or Factor*) and (Predict* or Model* or Decision* or Identif* or Prognos*)).mp. (4319291)
153 Decision*.mp. and ((Model* or Clinical*).mp. or Logistic Models/) (252494)
154 (Prognostic and (History or Variable* or Criteria or Scor* or Characteristic* or Finding* or Factor* or Model*)).mp. (261372)
155 predict*.tw,kf. (1765224)
156 "Predictive Value of Tests"/ (214833)
157 scor*.tw,kf. (1107444)
158 observ*.tw,kf. (3775138)
159 Observer Variation/ (43987)
160 (Stratification or Discrimination or Discriminate or "c statistic" or "Area under the curve" or AUC or Calibration or Indices or Algorithm or Multivariable).mp. (991374)
161 ROC Curve/ (65025)
162 or/150-161 (9340593)
163 exp cohort studies/ (2218162)
164 cohort?.mp. (803432)
165 Longitudinal Studies/ (150204)
166 (longitudinal* adj2 (study or studies)).mp. (201134)
167 Follow-up Studies/ (671986)
168 ((follow-up or followup) adj2 (study or studies)).mp. (702829)
169 Prospective Studies/ (596185)
170 (prospective* adj2 (study or studies)).mp. (727092)
171 Retrospective Studies/ (947473)
172 (retrospective* adj2 (study or studies)).mp. (1034984)
173 Controlled Before-After Studies/ (654)
174 (cba adj1 (design? or procedure? or study or studies)).mp. (148)
175 (before-after adj2 (design? or procedure? or study or studies)).mp. (2793)

176 (before adj1 after adj2 (study or studies)).mp. (2302)
177 Interrupted Time Series Analysis/ (1400)
178 (interrupt* adj1 time adj1 series).mp. (4389)
179 Cross-Over Studies/ (51603)
180 (cross over adj2 (design? or procedure? or study or studies or trial?)).mp. (60730)
181 or/163-180 (2778427)
182 162 or 181 (10529020)
183 149 and 182 (12235)
184 183 not (exp animals/ not exp humans/) (11299)
185 184 not ((exp infant/ or exp child/) not exp adult/) (10649)
186 limit 185 to (letter or "review" or "systematic review" or systematic reviews as topic) [Limit not valid in Ovid MEDLINE(R),Ovid MEDLINE(R) Daily Update,Ovid MEDLINE(R) PubMed not MEDLINE,Ovid MEDLINE(R) In-Process,Ovid MEDLINE(R) Publisher; records were retained] (1819)
187 185 not 186 (8830)
188 limit 187 to english language (8340)
189 limit 188 to yr="1999 -Current" (7192)
190 ("20191115" or "20191116" or "20191117" or "20191118" or "20191119" or 2019112* or 2019113* or 201912* or 2020* or 2021* or 2022*).dt,ez,da. (3547217)
191 189 and 190 (1916)

***************************

Database: Cochrane Central Register of Controlled Trials <2014 to Present>
Search Strategy:
--------------------------------------------------------------------------------
1 exp Central Nervous System Infections/ (1615)
2 (central nervous system adj2 infect*).tw,kw. (193)
3 (CNS adj2 infect*).tw,kw. (129)
4 ((brain or cerebral or cranial or intracranial or epidural or extradural or spine or spinal or mening*) adj2 abscess*).tw,kw. (117)
5 ((brain or cerebral or cranial or intracranial or epidural or extradural or spine or spinal or mening*) adj2 infect*).tw,kw. (759)
6 myeloencephaliti*.tw,kw. (1)
7 encephalomyeliti*.tw,kw. (234)
8 cerebromeningiti*.tw,kw. (0)
9 ((perimeningeal or parameningeal or encephalitis or myelitis) adj2 infect*).tw,kw. (37)
10 ((myelitis or myelitides or myelopathies or myelopathy) adj2 (inflamma* or infect* or necrotising)).tw,kw. (6)
11 (spinal adj3 (inflamm* or infect*)).tw,kw. (375)
12 (prion adj2 (diseas* or infect* or disorder*)).tw,kw. (33)
13 transmissible spongiform encephalopath*.tw,kw. (0)
14 transmissible dementia?.tw,kw. (0)
15 exp DNA Virus Infections/ (7271)
16 (dna adj2 vir* infect*).tw,kw. (14)
17 exp RNA Virus Infections/ (23542)
18 (rna adj2 vir* infect*).tw,kw. (10)
19 exp RNA Viruses/ (6977)

20 exp DNA Viruses/ (2785)
21 Virus Diseases/ (785)
22 Influenza, Human/ (2814)
23 (flu or grippe or influenza?).tw,kw. (11059)
24 exp Measles virus/ (82)
25 exp Measles/ (298)
26 measles.tw,kw. (1207)
27 (edmonston adj3 virus).tw,kw. (13)
28 exp Herpes Simplex/ (990)
29 exp Simplexvirus/ (362)
30 Herpesviridae Infections/ (86)
31 (herpes or herpesvirus* or herpesviridae or simplexvirus* or simplex virus* or hhv? or hsv?).tw,kw. (4525)
32 exp HIV Infections/ (13092)
33 exp HIV/ (3212)
34 HIV?.tw,kw. (28176)
35 human immunodeficiency virus*.tw,kw. (13074)
36 aids virus*.tw,kw. (10)
37 acquired immune deficiency syndrome virus*.tw,kw. (0)
38 acquired immunodeficiency syndrome virus*.tw,kw. (0)
39 htlv-iii.tw,kw. (10)
40 human t cell lymphotropic virus*.tw,kw. (21)
41 human t cell leukemia virus*.tw,kw. (24)
42 lymphadenopathy associated virus*.tw,kw. (1)
43 Herpesvirus 4, Human/ (104)
44 burkitts lymphoma virus*.tw,kw. (0)
45 (e b virus* or eb virus* or EBV).tw,kw. (711)
46 epstein barr virus*.tw,kw. (502)
47 burkitt herpesvirus.tw,kw. (0)
48 infectious mononucleosis virus*.tw,kw. (0)
49 hhv-4.tw,kw. (1)
50 hhv4.tw,kw. (0)
51 exp Adenoviridae/ (203)
52 adenovirus*.tw,kw. (847)
53 adenoviridae?.tw,kw. (147)
54 ichtadenovirus*.tw,kw. (0)
55 atadenovirus*.tw,kw. (0)
56 adenovirus*.tw,kw. (847)
57 apc virus*.tw,kw. (0)
58 exp Enterovirus/ (177)
59 enterovirus infections/ or exp coxsackievirus infections/ (144)
60 coxsackie virus*.tw,kw. (12)
61 coxsackievirus*.tw,kw. (35)

62 enterovirus*.tw,kw. (245)
63 herpangina?.tw,kw. (17)
64 (epidemic adj2 (myalgia? or peurodynia?)).tw,kw. (0)
65 (hand adj1 foot adj1 mouth adj1 diseas*).tw,kw. (138)
66 exp Neisseria/ (477)
67 neisseria?.tw,kw. (1089)
68 Pseudomonas aeruginosa/ (367)
69 Pseudomonas Infections/ (461)
70 pseudomonas aeruginosa.tw,kw. (2077)
71 pseudomonas pyocyanea.tw,kw. (4)
72 (pseudomonas adj2 infect*).tw,kw. (599)
73 exp Escherichia coli/ (683)
74 escherichia coli.tw,kw. (2653)
75 ecoli.tw,kw. (165)
76 e? coli.tw,kw. (1422)
77 eaggec.tw,kw. (3)
78 Streptococcus pyogenes/ (271)
79 streptococcus pyogenes.tw,kw. (251)
80 streptococcus group a.tw,kw. (71)
81 flesh-eating bacteria.tw,kw. (0)
82 exp Legionellosis/ (19)
83 legionellos#s.tw,kw. (6)
84 legionella pneumophila?.tw,kw. (73)
85 (legionnaire* adj1 disease?).tw,kw. (23)
86 pontiac fever?.tw,kw. (0)
87 exp Yersinia/ (12)
88 yersinia?.tw,kw. (49)
89 Candida albicans/ (218)
90 candida albican?.tw,kw. (680)
91 exp Candidiasis/ (958)
92 candidias#s.tw,kw. (1940)
93 monilias#s.tw,kw. (17)
94 Plasmodium vivax/ (114)
95 plasmodium vivax?.tw,kw. (443)
96 Plasmodium falciparum/ (865)
97 plasmodium falciparum?.tw,kw. (2381)
98 Entamoeba histolytica/ (23)
99 entamoeba histolytica?.tw,kw. (96)
100 exp Mycoplasma Infections/ (114)
101 eperythrozoonos#s.tw,kw. (1)
102 contagious pleuropneumonia?.tw,kw. (1)
103 (mycoplasma adj3 (infect* or pneumonia?)).tw,kw. (331)

104 primary atypical pneumonia?.tw,kw. (4)
105 exp Spirochaetales Infections/ (344)
106 ((spirochete or spirochaetales) adj2 infect*).tw,kw. (4)
107 ((spirochete or spirochaetales or borrelia? or burgdorferi or leptospira or treponemal) adj2 infect*).tw,kw. (55)
108 (lyme adj2 (diseas* or arthritis or borreliosis)).tw,kw. (229)
109 relapsing fever?.tw,kw. (25)
110 cane cutter fever?.tw,kw. (0)
111 leptospiros#s.tw,kw. (95)
112 stuttgart disease?.tw,kw. (0)
113 swineherd* disease?.tw,kw. (0)
114 weil?? disease?.tw,kw. (1)
115 icterohemorrhagic leptospiros#s.tw,kw. (0)
116 spirochetal jaundice?.tw,kw. (0)
117 bejel?.tw,kw. (0)
118 pinta.tw,kw. (4)
119 syphilis.tw,kw. (758)
120 great pox.tw,kw. (0)
121 frambesia?.tw,kw. (0)
122 yaws.tw,kw. (31)
123 Protozoan Infections/ (15)
124 histomonias#s.tw,kw. (0)
125 protozoan infection?.tw,kw. (16)
126 exp Helminthiasis/ (1388)
127 helminthias#s.tw,kw. (265)
128 nematomorpha infection?.tw,kw. (0)
129 or/1-128 (76268)
130 exp Neurocognitive Disorders/ or Cognition/ (8229)
131 exp Dementia/ (6277)
132 dement*.tw,kw. (14586)
133 alzheimer*.tw,kw. (12296)
134 (lewy* adj2 bod*).tw,kw. (476)
135 deliri*.tw,kw. (3868)
136 (chronic adj2 cerebrovascular).tw,kw. (640)
137 (chronic adj2 cerebrovascular).tw,kw. (640)
138 ('organic brain disease' or 'organic brain syndrome').tw,kw. (145)
139 ('normal pressure hydrocephalus' and 'shunt*').tw,kw. (86)
140 'benign senescent forgetfulness'.tw,kw. (2)
141 (cerebr* adj2 deteriorat*).tw,kw. (34)
142 (cerebral* adj2 insufficient*).tw,kw. (6)
143 (pick* adj2 disease).tw,kw. (40)
144 (creutzfeldt or jcd or cjd).tw,kw. (73)
145 huntington*.tw,kw. (742)

146 korsako*.tw,kw. (74)
147 ((cognitive or cognition or neurocognit* or mental) adj2 (disorder* or function* or dysfunction* or deteriorat* or decline? or impairment?)).tw,kw. (42020)
148 or/130-147 (64621)
149 129 and 148 (1145)
150 Validat*.mp. or Predict*.ti. or Rule*.mp. (76048)
151 (Predict* and (Outcome* or Risk* or Model*)).mp. (81287)
152 ((History or Variable* or Criteria or Scor* or Characteristic* or Finding* or Factor*) and (Predict* or Model* or Decision* or Identif* or Prognos*)).mp. (254067)
153 Decision*.mp. and ((Model* or Clinical*).mp. or Logistic Models/) (27771)
154 (Prognostic and (History or Variable* or Criteria or Scor* or Characteristic* or Finding* or Factor* or Model*)).mp. (18787)
155 predict*.tw,kw. (111614)
156 "Predictive Value of Tests"/ (7532)
157 scor*.tw,kw. (291489)
158 observ*.tw,kw. (294161)
159 Observer Variation/ (2156)
160 (Stratification or Discrimination or Discriminate or "c statistic" or "Area under the curve" or AUC or Calibration or Indices or Algorithm or Multivariable).mp. (91202)
161 ROC Curve/ (1317)
162 or/150-161 (757019)
163 exp cohort studies/ (156694)
164 cohort?.mp. (74324)
165 Longitudinal Studies/ (6586)
166 (longitudinal* adj2 (study or studies)).mp. (13330)
167 Follow-up Studies/ (61842)
168 ((follow-up or followup) adj2 (study or studies)).mp. (88760)
169 Prospective Studies/ (96612)
170 (prospective* adj2 (study or studies)).mp. (189833)
171 Retrospective Studies/ (9394)
172 (retrospective* adj2 (study or studies)).mp. (31435)
173 Controlled Before-After Studies/ (29)
174 (cba adj1 (design? or procedure? or study or studies)).mp. (27)
175 (before-after adj2 (design? or procedure? or study or studies)).mp. (1418774)
176 (before adj1 after adj2 (study or studies)).mp. (1379173)
177 Interrupted Time Series Analysis/ (32)
178 (interrupt* adj1 time adj1 series).mp. (402)
179 Cross-Over Studies/ (39169)
180 (cross over adj2 (design? or procedure? or study or studies or trial?)).mp. (58543)
181 or/163-180 (1422637)
182 162 or 181 (1515427)
183 149 and 182 (1060)
184 183 not (exp animals/ not exp humans/) (1060)
185 184 not ((exp infant/ or exp child/) not exp adult/) (1011)
186 limit 185 to (letter or "review" or "review literature" or review, academic or review, tutorial) (5)
187 185 not 186 (1006)

188 limit 187 to english language (635)
189 limit 188 to yr="1999 -Current" (595)
190 ("2019111" or "201912" or 2020* or 2021* or 2022*).up. (1137880)
191 189 and 190 (317)

***************************

Database: Embase <1974 to 2021 October 01>
Search Strategy:
--------------------------------------------------------------------------------
1 exp Central Nervous System Infection/ (183414)
2 (central nervous system adj2 infect*).tw,kw. (5251)
3 (CNS adj2 infect*).tw,kw. (5562)
4 ((brain or cerebral or cranial or intracranial or epidural or extradural or spine or spinal or mening*) adj2 abscess*).tw,kw. (11591)
5 ((brain or cerebral or cranial or intracranial or epidural or extradural or spine or spinal or mening*) adj2 infect*).tw,kw. (15697)
6 myeloencephaliti*.tw,kw. (272)
7 encephalomyeliti*.tw,kw. (24934)
8 cerebromeningiti*.tw,kw. (13)
9 ((perimeningeal or parameningeal or encephalitis or myelitis) adj2 infect*).tw,kw. (1804)
10 ((myelitis or myelitides or myelopathies or myelopathy) adj2 (inflamma* or infect* or necrotising)).tw,kw. (403)
11 (spinal adj3 (inflamm* or infect*)).tw,kw. (4731)
12 (prion adj2 (diseas* or infect* or disorder*)).tw,kw. (10313)
13 transmissible spongiform encephalopath*.tw,kw. (3483)
14 transmissible dementia?.tw,kw. (38)
15 exp DNA Virus Infection/ (323645)
16 (dna adj2 vir* infect*).tw,kw. (531)
17 exp RNA Virus Infection/ (918553)
18 (rna adj2 vir* infect*).tw,kw. (942)
19 exp RNA Virus/ (351037)
20 exp DNA Virus/ (267773)
21 Virus infection/ (164841)
22 exp Influenza/ (94336)
23 (flu or grippe or influenza?).tw,kw. (154756)
24 exp Measles virus/ (8690)
25 exp Measles/ (19062)
26 measles.tw,kw. (24998)
27 (edmonston adj3 virus).tw,kw. (136)
28 exp Herpes Simplex/ (34328)
29 Simplexvirus/ or exp Herpes simplex virus/ (36901)
30 Herpesviridae Infections/ (7173)
31 (herpes or herpesvirus* or herpesviridae or simplexvirus* or simplex virus* or hhv? or hsv?).tw,kw. (108217)
32 exp Human immunodeficiency virus infection/ (392086)

33 exp Human immunodeficiency virus/ (205970)
34 HIV?.tw,kw. (426730)
35 human immunodeficiency virus*.tw,kw. (102933)
36 aids virus*.tw,kw. (1142)
37 acquired immune deficiency syndrome virus*.tw,kw. (19)
38 acquired immunodeficiency syndrome virus*.tw,kw. (30)
39 htlv-iii.tw,kw. (1859)
40 human t cell lymphotropic virus*.tw,kw. (2753)
41 human t cell leukemia virus*.tw,kw. (3879)
42 lymphadenopathy associated virus*.tw,kw. (299)
43 Epstein Barr virus/ (42499)
44 burkitts lymphoma virus*.tw,kw. (0)
45 (e b virus* or eb virus* or EBV).tw,kw. (41039)
46 epstein barr virus*.tw,kw. (41317)
47 burkitt herpesvirus.tw,kw. (0)
48 infectious mononucleosis virus*.tw,kw. (4)
49 hhv-4.tw,kw. (74)
50 hhv4.tw,kw. (34)
51 exp Adenoviridae/ (12334)
52 adenovirus*.tw,kw. (56666)
53 adenoviridae?.tw,kw. (322)
54 ichtadenovirus*.tw,kw. (4)
55 atadenovirus*.tw,kw. (92)
56 adenovirus*.tw,kw. (56666)
57 apc virus*.tw,kw. (0)
58 exp Enterovirus/ (21077)
59 enterovirus infections/ or exp coxsackie virus infections/ (2948)
60 coxsackie virus*.tw,kw. (1394)
61 coxsackievirus*.tw,kw. (5894)
62 enterovirus*.tw,kw. (13321)
63 herpangina?.tw,kw. (269)
64 (epidemic adj2 (myalgia? or peurodynia?)).tw,kw. (24)
65 (hand adj1 foot adj1 mouth adj1 diseas*).tw,kw. (524)
66 exp Neisseria/ (33876)
67 neisseria?.tw,kw. (23998)
68 exp Pseudomonas aeruginosa/ (106183)
69 Pseudomonas Infections/ (4058)
70 pseudomonas aeruginosa.tw,kw. (78295)
71 pseudomonas pyocyanea.tw,kw. (33)
72 (pseudomonas adj2 infect*).tw,kw. (7280)
73 exp Escherichia coli/ (385086)
74 escherichia coli.tw,kw. (288528)

75 ecoli.tw,kw. (10223)
76 e? coli.tw,kw. (163876)
77 eaggec.tw,kw. (160)
78 Streptococcus pyogenes/ (16121)
79 streptococcus pyogenes.tw,kw. (10036)
80 streptococcus group a.tw,kw. (341)
81 flesh-eating bacteria.tw,kw. (47)
82 legionnaire disease/ (6283)
83 legionellos#s.tw,kw. (1509)
84 legionella pneumophila?.tw,kw. (6631)
85 (legionnaire* adj1 disease?).tw,kw. (3762)
86 pontiac fever?.tw,kw. (184)
87 exp Yersinia/ (16480)
88 yersinia?.tw,kw. (14546)
89 Candida albicans/ (59791)
90 candida albican?.tw,kw. (43804)
91 exp Candidiasis/ (51236)
92 candidias#s.tw,kw. (21482)
93 monilias#s.tw,kw. (253)
94 Plasmodium vivax/ (8830)
95 plasmodium vivax?.tw,kw. (7547)
96 exp Plasmodium falciparum/ (42630)
97 plasmodium falciparum?.tw,kw. (39297)
98 Entamoeba histolytica/ (9084)
99 entamoeba histolytica?.tw,kw. (7061)
100 exp Mycoplasma Infections/ (8127)
101 eperythrozoonos#s.tw,kw. (61)
102 contagious pleuropneumonia?.tw,kw. (49)
103 (mycoplasma adj3 (infect* or pneumonia?)).tw,kw. (11292)
104 primary atypical pneumonia?.tw,kw. (85)
105 exp Spirochaetales Infections/ (60214)
106 ((spirochete or spirochaetales) adj2 infect*).tw,kw. (170)
107 ((spirochete or spirochaetales or borrelia? or burgdorferi or leptospira or treponemal) adj2 infect*).tw,kw. (4135)
108 (lyme adj2 (diseas* or arthritis or borreliosis)).tw,kw. (13358)
109 relapsing fever?.tw,kw. (1321)
110 cane cutter fever?.tw,kw. (0)
111 leptospiros#s.tw,kw. (7581)
112 stuttgart disease?.tw,kw. (2)
113 swineherd* disease?.tw,kw. (2)
114 weil?? disease?.tw,kw. (368)
115 icterohemorrhagic leptospiros#s.tw,kw. (42)
116 spirochetal jaundice?.tw,kw. (1)

117 bejel?.tw,kw. (81)
118 pinta.tw,kw. (118)
119 syphilis.tw,kw. (25518)
120 great pox.tw,kw. (11)
121 frambesia?.tw,kw. (11)
122 yaws.tw,kw. (589)
123 Protozoan Infections/ (4608)
124 histomonias#s.tw,kw. (91)
125 protozoan infection?.tw,kw. (1076)
126 exp Helminthiasis/ (112655)
127 helminthias#s.tw,kw. (2349)
128 nematomorpha infection?.tw,kw. (3)
129 or/1-128 (2860746)
130 exp Neurocognitive Disorders/ or Cognition/ (1032496)
131 exp Dementia/ (388793)
132 dement*.tw,kw. (186879)
133 alzheimer*.tw,kw. (227092)
134 (lewy* adj2 bod*).tw,kw. (15534)
135 deliri*.tw,kw. (27691)
136 (chronic adj2 cerebrovascular).tw,kw. (1129)
137 (chronic adj2 cerebrovascular).tw,kw. (1129)
138 ('organic brain disease' or 'organic brain syndrome').tw,kw. (1036)
139 ('normal pressure hydrocephalus' and 'shunt*').tw,kw. (1986)
140 'benign senescent forgetfulness'.tw,kw. (31)
141 (cerebr* adj2 deteriorat*).tw,kw. (347)
142 (cerebral* adj2 insufficient*).tw,kw. (107)
143 (pick* adj2 disease).tw,kw. (4119)
144 (creutzfeldt or jcd or cjd).tw,kw. (9398)
145 huntington*.tw,kw. (26003)
146 korsako*.tw,kw. (1819)
147 ((cognitive or cognition or neurocognit* or mental) adj2 (disorder* or function* or dysfunction* or deteriorat* or decline? or impairment?)).tw,kw. (329801)
148 or/130-147 (1196213)
149 129 and 148 (67163)
150 Validat*.mp. or Predict*.ti. or Rule*.mp. (1617411)
151 (Predict* and (Outcome* or Risk* or Model*)).mp. (1540052)
152 ((History or Variable* or Criteria or Scor* or Characteristic* or Finding* or Factor*) and (Predict* or Model* or Decision* or Identif* or Prognos*)).mp. (5783288)
153 Decision*.mp. and ((Model* or Clinical*).mp. or statistical model/) (487113)
154 (Prognostic and (History or Variable* or Criteria or Scor* or Characteristic* or Finding* or Factor* or Model*)).mp. (413472)
155 predict*.tw,kw. (2371244)
156 exp predictive value/ (199814)
157 scor*.tw,kw. (1721214)
158 observ*.tw,kw. (4804895)

159 Observer Variation/ (20479)
160 (Stratification or Discrimination or Discriminate or "c statistic" or "Area under the curve" or AUC or Calibration or Indices or Algorithm or Multivariable).mp. (1535178)
161 receiver operating characteristic/ (153211)
162 or/150-161 (12282392)
163 cohort analysis/ (757911)
164 cohort?.mp. (1324044)
165 exp Longitudinal Study/ (161919)
166 (longitudinal* adj2 (study or studies)).mp. (217262)
167 Follow-up/ (1744008)
168 ((follow-up or followup) adj2 (study or studies)).mp. (88991)
169 Prospective Study/ (716248)
170 (prospective* adj2 (study or studies)).mp. (906306)
171 Retrospective Study/ (1139668)
172 (retrospective* adj2 (study or studies)).mp. (1240424)
173 (cba adj1 (design? or procedure? or study or studies)).mp. (191)
174 (before-after adj2 (design? or procedure? or study or studies)).mp. (3119)
175 (before adj1 after adj2 (study or studies)).mp. (2497)
176 Interrupted Time Series Analysis/ (203571)
177 (interrupt* adj1 time adj1 series).mp. (5078)
178 Crossover procedure/ (68275)
179 (cross over adj2 (design? or procedure? or study or studies or trial?)).mp. (25002)
180 or/163-179 (4401538)
181 162 or 180 (14132353)
182 149 and 181 (30309)
183 182 not ((exp animals/ or exp animal experimentation/ or nonhuman/) not exp human/) (26065)
184 183 not ((exp embryo/ or exp fetus/ or exp juvenile/) not exp adult/) (23340)
185 limit 184 to (conference abstract or conference paper or "conference review") (5995)
186 184 not 185 (17345)
187 limit 186 to (books or chapter or letter or "review") (3860)
188 186 not 187 (13485)
189 188 not medline.cr. (12168)
190 limit 189 to english language (11731)
191 limit 190 to yr="1999 -Current" (10912)
192 ("20191115" or "20191116" or "20191117" or "20191118" or "20191119" or 2019112* or 2019113* or 201912* or 2020* or 2021* or 2022*).dc. (3944917)
193 191 and 192 (3027)

***************************
