# Supplementary material for: Treating cognitive impairments in primary central nervous system infections: A systematic review of pharmacological interventions
Source: Medicine (Baltimore). 2023 Jul 14;102(28):e34151. doi: 10.1097/MD.0000000000034151 (PMC10344564; doi:10.1097/MD.0000000000034151)
Supplement: Supplementary file 2 [file medi-102-e34151-s002.pdf]

Table S2. Summary of study characteristics

1) Author (Year)<br>2) Setting<br>3) Country<br>4) Study funding	1) Infectious agent<br>2) Study Objectives<br>3) Groups (Dosage)<br>4) Intervention Type<br>5) Duration of Intervention<br>6) Other Co-Occurring Interventions	1) Study Design<br>2) Inclusion Criteria<br>3) Exclusion Criteria	1) Sample size (n)<br>Attrition<br>Age (years)<br>Sex (%M)<br>Duration of Illness (DOI; years)<br>2) Comparisons at BL	1) Assessment measure<br>2) Outcome measure	Statistical method	Results		1) Adherence (%)<br>2) Adverse effect
						Pre- intervention (BL); Post- intervention (FU, t_1, t_2, etc.)<br>Change from Pre to Post	Group comparisons	
1) Prasad KM, Eack SM, Keshavan MS, Yolken RH, Iyengar S, Nimgaonkar VL. (2013) [36]<br>2) Western Psychiatric Inst. & Clinic, Pittsburgh, & Wayne State University, Detroit<br>3) USA<br>4) SMRI & NIMH	1) HSV-1<br><br>2) To test if VAV + AP ↑ cog performance & psychopathology in Scz pts w/ HSV-1<br><br>3) G1: VAV + AP (3g/day)<br>G2: PLA +AP<br><br>4) Drug (VAV)<br><br>5) 18 wks<br><br>6) NR	1) Double-blind PLA-RCT<br><br>2) (+) test for HSV-1 IgG Ab; Stable doses of APs for ≥1 mo; Score ≥4 on ≥1 item in PANSS<br><br>3) Pts meeting DSM-IV substance abuse, dependence, mental retardation criteria; Medical/neurological, immune disorders; On immunosuppressants, regular antiherpes treatment	1) G1 (n = 12)<br>Attrition = 25%<br>Age: 29.54 ± 9.44<br>Sex: 50% M<br>DOI: 3.49 ± 2.82<br><br>G2 (n = 12)<br>Attrition = 33%<br>Age: 28.67 ± 8.47<br>Sex: 58% M<br>DOI: 5.59 ± 3.08<br><br>2) Age sex, SES, DOI, & PANSS	1) PennCNB<br><br>2) Working memory (total & 2-back)<br>Immediate verbal memory (processing speed & accuracy)	Completer analysis<br><br>ITT analysis	IIT:<br>G1<br>*Pre;Post (mean ± SD)*<br>NR<br>*Change*<br>Working memory (total)<br>3.76 ± 4.90/0.25 ± 4.90<br>Working memory (2-back)<br>2.13 ± 2.68/0.13 ± 2.97<br>Verbal memory (processing speed) −0.11 ± 0.09/−0.01 ± 0.08<br>Verbal memory (accuracy)<br>1.03 ± 2.98/−0.38 ± 3.64<br><br>Completer Analysis<br>G1<br>*Pre;Post (mean ± SD)*<br>NR<br>*Change*<br>Working memory (total)<br>2.50 ± 4.16<br>Working memory (2-back)<br>1.56 ± 2.41<br>Verbal memory (processing speed) −0.09 ± 0.09<br>Verbal memory (accuracy)<br>0.99 ± 2.75<br><br>G2<br>*Pre;Post (mean ± SD)*	G1 v. G2<br>↑ in working + verbal memory & visual obj learning<br><br>ITT<br>β; $F(df)$; p; *Cohen's d*<br>Working memory (total)<br>3.86; 2.71 (1, 127); NS; 0.79<br>Working memory (2-back)<br>2.25; 4.42 (1, 190); 0.037; 0.72<br>Verbal memory (processing speed)<br>−0.097; 4.49 (1, 169); 0.036; 1.21<br>Verbal memory (accuracy)<br>1.23; 0.45 (1, 162); NS; 0.38<br><br>Completer Analysis<br>β; $F(df)$; p; *Cohen's d*<br>Working memory (total)<br>1.80; 3.24 (1, 11); 0.099; 0.79<br>Working memory (2-back)<br>2.41; 4.23 (1, 11); 0.064; 0.82<br>Verbal memory (processing speed)<br>−1.88; 3.53 (1, 11); 0.087; 1.06<br>Verbal memory (accuracy)<br>0.59; 0.34 (1, 11); NS; 0.33	1) NR<br><br>2)<br>G1: constipation, stomach pain, motion sickness, occasional muscle twitch, tremor, and upset stomach<br><br>G2: leg cramps<br><br>Reported within both groups: drooling, muscle tightness, mild tremors, akathisia, bloating of the stomach, feeling tired, elbow pain, increased sexual drive, insomnia

						NR<br>*Change*<br>Working memory (total)<br>−1.06 ± 5.85<br>Working memory (2-back)<br>−0.86 ± 3.33<br>Verbal memory (processing speed) −0.02 ± 0.08<br>Verbal memory (accuracy)<br>0.01 ± 4.03		
1) Berende A, Ter Hofstede HJM, Vos FJ, et al.<br>(2019) [23]<br>2) Sint Maartenskliniek & Radboud University Medical Center<br>3) Netherlands<br>4) NNOSR	1) LD<br><br>2) To test if LT AB treatment+2 wks of CTX followed by 12 wks of DOX or CLR-HCQ improves cog performance in pts with LD compared to ST AB treatment+2 wks of CTX.<br><br>3) G1: CTX + DOX (NR)<br>G2: CTX + CLR-HCQ (NR)<br>G3: CTX + PLA<br><br>4) Drug (DOX or CLR-HCQ)<br><br>5) 14 wks<br><br>6) NR	1) PLA-RCT<br><br>2) M or non-pregnant, non-lactating F ≥18; MSK pain, arthritis, arthralgia, neuralgia, sensory disturbances, neuropsychological/cog disorders, w/ or w/o persistent fatigue, that are: either temporally related to episode of EM or proven symptomatic LD or accompanied by (+) B. burgdorferi IgG or IgM immunoblot<br><br>3) Allergy/intolerance to TCN, macrolides, HCQ, or CTX; AMT for > 5 d. DX requiring IV AMT; HIV(+)/other immune (+)syphilis/other spirochetal, mod/sev liver, rheumatic, CFS, chronic pain DX; On cisapride, astemizole, terfenadine,	1) G1 (n = 86)<br>Attrition = 23%<br>Age: 48.3 ± 12.6<br>Sex: 54% M<br>DOI: 2.7*<br><br>G2 (n = 96)<br>Attrition = 25%<br>Age: 47.5 ± 13.0<br>Sex: 57% M<br>DOI: 2.8*<br><br>G3 (n = 98)<br>Attrition = 26%<br>Age: 50.3 ± 9.9<br>Sex: 52% M<br>DOI = 2.3*<br><br>2) Age, sex, %white, current SX, duration of SX, history of LD, previous AMT, Edu, EMPL, & Cog domain compound score	1) RAVLT, DST, TMT-A, SCWT, DSST & CFT<br><br>2) Episodic memory<br>Attention/Working memory<br>Verbal fluency<br>Speed of info processing<br>Executive function	Modified ITT<br><br>Multivariate Normative Comparison<br><br>Covariance<br><br>Linear Mixed Models	G1<br>*BL; 14 wks, mean (95% CI)*<br>Episodic memory<br>−0.08 (−0.29 to 0.12); 0.19 (−0.03 to 0.35)<br>Attention/Working memory<br>−0.11 (−0.35 to 0.13); 0.21 (0.05 to 0.37)<br>Verbal fluency<br>−0.09 (−0.31 to 0.13); 0.18 (−0.02 to 0.35)<br>Speed of info processing<br>0.00 (−0.20 to 0.19); 0.25 (0.15 to 0.36)<br>Executive function<br>−0.02 (−0.21 to 0.17); 0.14 (−0.01 to 0.28)<br>*Change* NR<br><br>G2<br>*BL; 14 wks, mean (95% CI)*<br>Episodic memory<br>0.06 (−0.12 to 0.24); 0.27 (0.13 to 0.41)<br>Attention/Working memory<br>0.26 (0.06 to 0.46); 0.16 (0.01 to 0.30)<br>Verbal fluency<br>−0.01 (−0.22 to 0.20); 0.24 (0.09 to 0.39)<br>Speed of info processing	*p*<br>Episodic memory<br>NS<br>Attention/Working memory<br>NS<br>Verbal fluency<br>NS<br>Speed of info processing<br>NS<br>Executive function<br>NS<br><br>After 14 wks: no SGFNT difference b/w treatments for any cog domains (p = 0.49–0.82).<br>At FU: no additional treatment effect (p = 0.35–0.98) or difference b/w groups (p = 0.37–0.93).<br>↑ cog performances at wks 14, 26, & 40 compared to BL: not specific to a TX group.<br>LT AB TX ≠ better cog performance than ST AB TX.	1) NR<br><br>2) NR

		barbiturates, phenytoin, carbamazepine; In drug trials; Sev comorbidity; Insufficient in Dutch; Unwilling to use contraception other than oral				0.25 (0.15 to 0.36); 0.30 (0.21 to 0.39)<br>Executive function<br>0.14 (−0.01 to 0.28); 0.13 (0.00 to 0.27)<br>*Change* NR<br><br>G3<br>*BL; 14 wks, mean (95% CI)*<br>Episodic memory<br>0.19 (0.02 to 0.37); 0.27 (0.12 to 0.42)<br>Attention/Working memory<br>0.06 (−0.17 to 0.29); 0.25 (0.10 to 0.40)<br>Verbal fluency<br>0.18 (−0.08 to 0.27); 0.13 (−0.03 to 0.28)<br>Speed of info processing<br>0.10 (−0.08 to 0.27); 0.34 (0.24 to 0.44)<br>Executive function<br>0.11 (−0.06 to 0.27); 0.19 (0.05 to 0.32)<br>*Change* NR		
1) Breier A, Dickerson F, Buchanan R, et al. (2018) [25]<br>2) Indiana University School of Medicine<br>3) USA<br>4) SMRI	1) HSV-1<br><br>2) To test if VAV ↑ cog performance in Scz pts w/ HSV-1.<br><br>3) G1: HSV+ & PLA<br>G2: HSV+ & VAV (3g/day)<br>G3: HSV- & PLA<br>G4: HSV- & VAV<br><br>4) Drug (VAV)<br><br>5) 16 wks<br><br>6) NR	1) Double-blind PLA-RCT<br><br>2) DSM IV-TR Scz, schizophreniform, or schizoaffective DX; ≤4 CGI-S score; No illness which requires intensification of psychiatric care; No changes in AP 4 wks before randomization<br><br>3) DSM-IV-TR substance dependence DX (exception: nicotine/ caffeine);	1) G1 (n = 40)<br>Attrition = 32.5%<br>Age: 30.0 $\pm$ 6.2<br>Sex: 65% M<br>DOI: 4.4 $\pm$ 2.3<br><br>G2 (n = 34)<br>Attrition = 26.5%<br>Age: 29.9 $\pm$ 6.1<br>Sex: 58.8% M<br>DOI: 4.2 $\pm$ 2.7<br><br>G3 (n = 46)<br>Attrition = 30.4%<br>Age: 25.4 $\pm$ 4.9<br>Sex: 89.1% M<br>DOI: 3.6 $\pm$ 2.1	1) MATRICS & MCCB<br><br>2) Working memory<br>Visuospatial memory<br>Letter-number sequencing<br>Spatial span	ITT analysis<br><br>ANCOVA	G1<br>*BL; 8 wks; 16 wks, mean* $\pm$ *SD*<br>MATRICS Working memory<br>34.1 $\pm$ 1.8; 34.7 $\pm$ 1.8; 36.4 $\pm$ 1.8<br>MATRICS Visuospatial memory<br>33.9 $\pm$ 1.9; 34.6 $\pm$ 1.9; 37.3 $\pm$ 1.9<br>MATRICS Letter-number sequencing<br>35.6 $\pm$ 1.6; 35.8 $\pm$ 1.6; 39.5 $\pm$ 1.6<br>MATRICS Spatial span<br>38.1 $\pm$ 1.7; 39.3 $\pm$ 1.7; 38.1 $\pm$ 1.7<br>MATRICS Overall composite	$F(df);p$<br>MATRICS Working memory<br>3.47; 0.032<br>MATRICS Visuospatial memory<br>1.35; NS<br>MATRICS Letter-number sequencing<br>4.98; 0.007<br>MATRICS Spatial span<br>0.99; NS<br>MATRICS Overall composite<br>0.49; NS<br><br>VAV failed to demonstrate significant treatment effects on the two primary cog outcome	1) 83.3%<br><br>2) NR but 18.8% discontinued due to "adverse event"

		High suicide risk; Serious active medical condition affecting brain/cog functioning; IQ < 70; Current acute, serious, unstable medical conditions	G4 (n = 50)<br>Attrition = 24%<br>Age: 27.4 ± 6.0<br>Sex: 76% M<br>DOI: 3.3 ± 1.90<br><br>2) Age, sex, gender, lifetime AP log, DOI, race, Edu, current EMPL, MATRICS, & PANSS			29.4 ± 2.1; 30.0 ± 2.1; 32.6 ± 2.1<br>*Change* NR<br><br>G2<br>*BL; 8 wks; 16 wks, mean ± SD*<br>MATRICS Working memory<br>31.8 ± 1.9; 34.4 ± 1.9; 34.7 ± 1.9<br>MATRICS Visuospatial memory<br>37.1 ± 2.1; 38.2 ± 2.1; 39.6 ± 2.1<br>MATRICS Letter-number sequencing<br>34.2 ± 1.7; 37.0 ± 1.7; 37.5 ± 1.7<br>MATRICS Spatial span<br>35.9 ± 1.8; 37.1 ± 1.8; 37.3 ± 1.8<br>MATRICS Overall composite<br>27.8 ± 2.3; 27.8 ± 2.3; 29.6 ± 2.3<br>*Change* NR<br><br>G3<br>*BL; 8 wks; 16 wks, mean ± SD*<br>MATRICS Working memory<br>35.5 ± 1.8; 39.5 ± 1.8; 39.0 ± 1.8<br>MATRICS Visuospatial memory<br>34.2 ± 1.9; 38.0 ± 1.9; 41.3 ± 1.9<br>MATRICS Letter-number sequencing<br>38.6 ± 1.6; 42.2 ± 1.6; 41.3 ± 1.6<br>MATRICS Spatial span<br>37.5 ± 1.7; 40.0 ± 1.7; 40.8 ± 1.7<br>MATRICS Overall composite	measures: the MCCB working memory composite & visuospatial memory scores. VAV add-on therapy may be beneficial for cog impairments but not psychotic SX.	

						30.2 ± 2.2; 33.3 ± 2.2; 36.4 ± 2.2<br>*Change* NR<br><br>G4<br>*BL; 8 wks; 16 wks, mean ± SD*<br>MATRICS Working memory<br>40.7 ± 1.6; 41.6 ± 1.6; 42.6 ± 1.6<br>MATRICS Visuospatial memory<br>39.6 ± 1.7; 39.4 ± 1.7; 42.2 ± 1.7<br>MATRICS Letter-number sequencing<br>42.0 ± 1.5; 43.1 ± 1.5; 43.7 ± 1.5<br>MATRICS Spatial span<br>42.5 ± 1.5; 42.9 ± 1.5; 44.1 ± 1.5<br>MATRICS Overall composite<br>34.8 ± 1.9; 35.6 ± 1.9; 39.2 ± 1.9<br>*Change* NR		
1) Fallon BA, Tager F, Fein L, Liegner K, Keilp J, Weiss N, Liebowitz MR. (1999) [17]<br>2) NR<br>3) USA<br>4) LDANJ & NPIRSG	1) LD<br><br>2) To test if repeated AB treatment over 4-mo interval ↑ cog functioning LD pts.<br><br>3) G1: No Treatment<br>G2: Oral AB (NR)<br>G3: IV-AB (NR)<br>G4: IM-AB (NR)<br><br>4) Drug (AB)<br><br>5) 16 wks<br><br>6) NR	1) Uncontrolled Study<br><br>2) Diagnosed & treated LD w/ complaints of persistent cog SX; ≥4 & ≤16 wks of IV-AB before study enrollment<br><br>3) NR	1) G1 (n = 5)<br>Attrition = CD<br><br>G2 (n = 7)<br>Attrition = CD<br><br>G3 (n = 7)<br>Attrition = CD<br><br>G4 (n = 4)<br>Attrition = CD<br><br>Age: 42.7 ± 13.25<br>Sex: 30% M<br>DOI: 1.78 ± 1.85<br><br>2) NR	1) WAIS, WMS, & COWAT<br><br>2) Verbal memory<br>Visual memory<br>Attention<br>Delayed memory<br>General memory<br>Verbal fluency	Paired Sample t-tests<br><br>ANOVA<br><br>Tukey's HSD<br><br>Pearson Correlation<br><br>ANCOVA	G2, G2 & G4<br>*BL; Time 2, mean ± SD*<br>Verbal memory<br>92.96 ± 19.1; 102.36 ± 14.9<br>Visual memory<br>104.16 ± 19.5; 110.46 ± 12.7<br>General memory<br>95.16 ± 16.7; 106.66 ± 14.9<br>Attention/concentration<br>101.36 ± 17.9; 108.46 ± 12.6<br>Delayed memory<br>94.96 ± 16.9; 109.96 ± 15.8<br>Verbal fluency<br>42.26 ± 17.2; 45.46 ± 15.9<br><br>*Change* NR	$t - score\ (df); p$<br>Verbal memory<br>−3.09 (17); 0.007<br>Visual memory<br>−1.50 (17); NS<br>General memory<br>−4.57 (17); <0.001<br>Attention/concentration<br>−1.96 (17); 0.066<br>Delayed memory<br>−4.14 (17); 0.001<br>Verbal fluency<br>−1.03 (17); NS<br><br>T2: G1 overall & individual cog score ↑ than G2; G3 greatest ↑ in cog; No SGNFT correlation b/w DOT on AB &	1) NR<br><br>2) NR

							↑ composite z-score; Repeated AB treatment may ↑ cog in pts	
1) Fallon BA, Keilp JG, Corbera KM, et al. (2008) [29]<br>2) New York State Psychiatric Institute & Columbia University Medical Center<br>3) USA<br>4) NINDS	1) LD<br><br>2) To test the effect of additional IV-AB (CTX) ↑ cog function in pts w/ LD.<br><br>3) G1: IV CTX (2g/d)<br>G2: IV PLA<br><br>4) Drug (AB)<br><br>5) 10 wks<br><br>6) NR	1) PLA-RCT<br><br>2) LD (+)/(-) controls; (+) IgG Western blot/(-) IgG western blot controls; ≥3 wks of IV-CTX; Memory impairment/no impairment for controls<br><br>3) Learning disability/medical condition; Cephalosporin allergy/history of major psychiatric disorder before LD onset	1) G1 (n = 23)<br>Attrition = 13%<br>Age: 45.3 ± 13.7<br>Sex: 39% M<br><br>G2 (n = 14)<br>Attrition = 14%<br>Age: 44.8 ± 12.7<br>Sex: 42.9% M<br><br>DOI: 9.0 ± 6.8<br><br>2) Age, sex, years of Edu, %white, EMPL, LD SX history, prior IV-AB treatment, prior oral-AB treatment, rheumatology exam, & neurology exam	1) WMS- III, N-Back Test, BVRT, BSRT, LRT, CPT, Stroop task, COWAT, CFT, finger tapping, SRT, CRT, TMT-A, TMT-B & digital symbol<br><br>2) Motor<br>Psychomotor<br>Attention<br>Verbal memory<br>Visual memory<br>Working memory<br>Fluency	ITT analysis<br><br>t tests<br><br>AVCOVA<br><br>χ^2	G1<br>*BL; 12 wks; 24 wks, mean ± SD*<br>Motor<br>−0.23 ± 1.34; 0.58 ± 0.88; 0.33 ± 1.05<br>Psychomotor<br>−0.21 ± 0.75; 0.19 ± 0.89; 0.12 ± 0.88<br>Attention<br>−0.12 ± 0.76; 0.15 ± 0.80; 0.18 ± 0.83<br>Verbal memory<br>−1.13 ± 1.33; −0.79 ± 1.71; −0.98 ± 1.44<br>Visual memory<br>−0.36 ± 1.21; −0.08 ± 1.2; −0.26 ± 1.43<br>Working memory<br>−0.92 ± 1.09; −0.42 ± 0.94; −0.54 ± 0.89<br>Fluency<br>−0.73 ± 0.94; −0.38 ± 1.04; −0.3 ± 0.98<br><br>*Change* NR<br><br>G2<br>*BL; 12 wks; 24 wks, mean ± SD*<br>Motor<br>−0.06 ± 1.19; 0.06 ± 1.31; 0.36 ± 0.64	G1<br>*Effect size, 12 wks; 24 wks*<br>Motor<br>0.67; 0.70<br>Psychomotor<br>0.54; 0.56<br>Attention<br>0.27; 0.36<br>Verbal memory<br>0.42; 0.31<br>Visual memory<br>0.25; 0.16<br>Working memory<br>0.52; 0.33<br>Fluency<br>0.55; 1.1<br><br>G2<br>*Effect size, 12 wks; 24 wks*<br>Motor<br>0.13; 0.49<br>Psychomotor<br>0.58; 0.88<br>Attention<br>0.40; 0.35<br>Verbal memory<br>−0.13; −0.18<br>Visual memory<br>0.22; 0.29<br>Working memory<br>−0.34; −0.37	1) NR<br><br>2)<br>G1: thrombus, hemolytic anemia, 3 additional Pts discontinued due to "adverse event"<br>G2: Systemic infection, intolerable joint pain

						Psychomotor<br>−0.16 ± 0.61; 0.14 ± 0.57; 0.29 ± 0.71<br>Attention<br>0.04 ± 1.20; 0.34 ± 0.70; 0.37 ± 0.92<br>Verbal memory<br>−0.78 ± 1.37; −0.72 ± 1.44; −0.86 ± 1.26<br>Visual memory<br>0.06 ± 1.09; 0.33 ± 0.73; 0.42 ± 0.62<br>Working memory<br>−0.32 ± 0.73; −0.37 ± 0.75; −0.04 ± 0.70<br>Fluency<br>−0.80 ± 0.38; −0.49 ± 0.39; −0.46 ± 0.44<br><br>*Change* NR	Fluency<br>0.53; 0.60<br><br>12 wks: G1 ↑ in all cog domains; 24 wks: G1 & G2 ↑ in cog; IV CTX = ST ↑ in cog for LD pts	
1) Bhatia T, Wood J, Iyengar S, et al. (2018) [24]<br>2) Dr. Ram Manohar Lohia Hospital, Delhi Delhi<br>3) India<br>4) DSTGI, SMRI & NIH	1) HSV-1<br><br>2) To test patterns of temporal change in cog functions among persons w/ & w/o HSV-1.<br><br>3) G1: AP + VAV (3g/d)<br>G2: AP + PLA<br><br>4) Drug (VAV)<br><br>5) 16 wks<br><br>6) NR	1) PLA-RCT<br><br>2) (+) HSV-1-specific IgG; Stable doses of APs for ≥1 mo; Score ≥4 on ≥1 item of PANSS.<br><br>3) DSM-IV substance abuse, dependence, mental retardation; History of medical, neurological illnesses, immune disorders; On immunosuppressants, regular antiherpes treatment	1) G1 (n = 30)<br>Attrition = 17%<br>Age: 31.77 ± 8.55<br>Sex: 50% M<br>DOI: 4.87 ± 2.01<br><br>G2 (n = 32)<br>Attrition = 3.1%<br>Age: 30.75 ± 8.68<br>Sex: 56% M<br>DOI: 4.96 ± 2.35<br><br>2) Age, sex, HOH occupation, diagnostic status, DOI	1) PennCNB & EMOD<br><br>2) Abstraction & mental flexibility<br>Attention<br>Face memory<br>Spatial memory<br>Working memory<br>Spatial ability<br>Sensorimotor<br>Emotion	ITT analysis	G1<br>*BL; T1; T2 (mean ± SD)*<br>Abstraction & mental flexibility<br>−1.74 ± 0.575; −1.78 ± 0.847; −1.8 ± 0.408<br>Attention<br>−0.81 ± 1.25; −0.74 ± 0.864; −0.95 ± 1.191<br>Face memory<br>−0.52 ± 1.122; −0.5 ± 1.503; −0.17± 1.579<br>Spatial memory<br>−0.69 ± 0.93; −0.54 ± 0.811; −0.48± 0.918<br>Working memory<br>−1.67 ± 1.398; −1.38 ± 0.97; −1.24± 1.091<br>Spatial ability<br>0.06 ± 0.539; 0.07 ± 0.704; 0.08 ± 0.76<br>Sensorimotor	BL: No SGNFT differences b/w G1 & G2 cog functions except spatial ability (p=0.021);<br>Group 1 ↑ in EMOD (p=0.048, Cohen's d = 0.43) but not other cog functions	1) NR<br><br>2)<br>G1: vomiting, nausea<br><br>G2: dizziness<br><br>Reported within both groups: constipation, body ache, 'rolling of eyes'

						−0.28 ± 1.579; 0.07 ± 1.269; 0.58± 0.929<br>Emotion<br>−1.13 ± 1.118; −0.78 ± 1.086; −1.0 ± 1.041<br><br>*Change* NR<br><br>G2<br>*BL; T1; T2 (mean ± SD)*<br>Abstraction & mental flexibility<br>−1.88 ± 0.833; −1.73 ± 0.74; −1.66 ± 0.721<br>Attention<br>−0.59 ± 0.959; −0.5 ± 0.978; −0.71 ± 1.122<br>Face memory<br>−0.78 ± 1.289; −0.83 ± 1.416; −0.47± 1.697<br>Spatial memory<br>−0.87 ± 0.718; −0.53 ± 0.86; −0.73± 0.944<br>Working memory<br>−1.45 ± 1.179; −1.17 ± 1.416; −1.17± 1.466<br>Spatial ability<br>−0.5 ± 0.516; −0.29 ± 0.825; −0.31± 0.602<br><br>Sensorimotor<br>−0.03 ± 1.356; 0.5 ± 0.938; 0.48± 0.949<br>Emotion<br>−0.97 ± 0.836; −0.97 ± 0.809; −1.07 ± 0.842<br><br>*Change* NR		

1) Kaplan RF, Trevino RP, Johnson GM, et al. (2003) [31]<br>2) NR<br>3) USA<br>4) NIAID, NIH & HHS	1) LD<br><br>2) To test if intensive AB therapy ↑ cog in pts w/ LD.<br><br>3) G1: LD(+) & AB (2g/d)<br>G2: LD(+) & PLA<br>G3: LD(-) & AB<br>G4: LD(-) & PLA<br><br>4) Drug (IV CTX)<br><br>5) 13 wks<br><br>6) NR	1) Double-blind PLA-RCT<br><br>2) (+)western blot IgG/EM skin lesion for (-) controls; History of acute LD; History of single or multiple EM skin lesions, early neurologic or cardiac SX attributed to LD, radiculoneuropathy, or Lyme arthritis; Previous treatment for LD<br><br>3) Hypersensitivity to study medications; Previously received CTX or cefotaxime; Inflammatory synovitis, coexisting condition; Medication that interferes with treatment regimen; (+)PCR for *B. burgdorferi* DNA in plasma or CSF at BL	1) G1 (n = 39)<br>Attrition = 23%<br><br>G2 (n = 39)<br>Attrition = 31%<br><br>G3 (n = 25)<br>Attrition = 20%<br><br>G4 (n = 26)<br>Attrition = 19%<br><br>Age: >18<br>Sex: 51% M<br>DOI: 4.01 ± 2.89<br><br>2) Age, edu, sex, years infected	1) RAVLT, BVRT, SDMT, & CalCAP<br><br>2) Attention & Speed of info processing<br>Learning & memory<br>Word fluency	Independent t-tests<br><br>Tukey Method<br><br>F statistic	G1 & G3<br>*BL; 80d; 180d (mean ± SD)*<br>Learning & memory<br>AVLT trial 1<br>6.0 ± 1.5; 6.3 ± 1.5; 6.4 ± 2.0<br>AVLT trial 5<br>11.9 ± 2.5; 11.6 ± 2.3; 11.9 ± 2.6<br>AVLT total score<br>48.7 ± 9.8; 48.3 ± 9.3; 49.6 ± 11.3<br>AVLT trial B<br>5.4 ± 1.8; 5.9 ± 2.0; 6.0 ± 1.9<br>AVLT short delay<br>9.5 ± 2.9; 9.3 ± 3.1; 10.0 ± 3.0<br>AVLT long delay<br>9.7 ± 3.2; 9.2 ± 3.3; 9.6 ± 3.3<br>AVLT recognition<br>13.0 ± 2.2; 13.2 ± 2.3; 13.3 ± 2.1<br>BVRT correct<br>7.1 ± 1.7; NA; 6.8 ± 2.0<br>BVRT errors<br>4.2 ± 2.9; NA; 3.9 ± 2.9<br>Attention & Speed of info<br>SDMT written<br>49.5 ± 8.8; 52.51 ± 9.3; 52.5 ± 9.2<br>SDMT oral<br>56.3 ± 10.9; 59.5 ± 12.7; 60.1 ± 11.8<br>CalCAP simple RT<br>46.5 ± 11.1; 48.6 ± 12.0; 46.9 ± 13.4<br>CalCAP choice RT<br>42.5 ± 13.4; 46.6 ± 10.7; 44.1 ± 11.6<br>CalCAP Seq RT1<br>47.4 ± 11.3; 49.5 ± 10.3; 48.9 ± 10.1<br>CalCAP Seq RT2	↑ cog functioning not specific to group, treatment or interaction effects; Sero(+) & sero(-) Pts ↑ cog functioning; Additional AB = no ↑ cog function	1) 78%<br><br>2) NR

						47.7 ± 9.4; 49.6 ± 9.6; 49.7 ± 9.7<br>Word fluency<br>COWA<br>37.6 ± 13.0; 41.6 ± 14.3; 41.9 ± 14.0<br><br>G2 & G4<br>*BL; 80d; 180d (mean ± SD)*<br>Learning & memory<br>AVLT trial 1<br>6.2 ± 2.1; 6.0 ± 1.9; 6.2 ± 2.3<br>AVLT trial 5<br>11.1 ± 2.7; 10.5 ± 2.7; 11.3 ± 2.5<br>AVLT total score<br>46.6 ± 10.7; 44.1 ± 11.1; 47.6 ± 10.1<br>AVLT trial B<br>5.6 ± 2.0; 5.6 ± 1.8; 6.0 ± 2.2<br>AVLT short delay<br>8.9 ± 3.3; 8.6 ± 3.4; 9.2 ± 2.8<br>AVLT long delay<br>9.2 ± 3.4; 8.5 ± 3.4; 9.0 ± 2.9<br>AVLT recognition<br>14.3 ± 6.14; 12.4 ± 2.5; 13.4 ± 1.8<br>BVRT correct<br>6.9 ± 1.5; NA; 6.7 ± 2.07<br>BVRT errors<br>4.8 ± 2.7; NA; 4.4 ± 3.4<br>Attention & Speed of info<br>SDMT written<br>48.3 ± 9.6; 51.5 ± 9.7; 53.2 ± 10.4<br>SDMT oral<br>55.6 ± 11.4; 59.2 ± 12.8; 60.6 ± 13.2<br>CalCAP simple RT<br>40.0 ± 15.8; 43.5 ± 14.8; 45.4 ± 11.1<br>CalCAP choice RT		

						42.5 ± 17.1; 45.0± 14.4; 45.0 ± 14.6<br>CalCAP Seq RT1<br>47.8 ± 11.9; 49.5 ± 10.3; 49.0 ± 10.4<br>CalCAP Seq RT2<br>48.4 ± 8.4; 49.77 ± 9.7; 51.3 ± 10.0<br>Word fluency<br>COWA<br>40.1 ± 12.7; 44.5 ± 12.6; 45.1 ± 13.1<br><br>*Change* NR		
1) Krupp LB, Hyman LG, Grimson R, et al. (2003) [32]<br>2) Suffolk County, Long Island<br>3) USA<br>4) NCRR, NIAID, NIH & HHS	1) LD<br><br>2) To test if AB ↑ cog functioning in pts w/ LD who already received one or more courses of treatment (have Post Lyme Syndrome).<br><br>3) G1: AB (2g/d)<br>G2: PLA<br><br>4) Drug (AB)<br><br>5) 4 wks<br><br>6) NR	1) Double-blind PLA-RCT<br><br>2) History of EM/LD; ≥ 3 wks oral-AB/IV-CTX; Severe fatigue.<br><br>3) Medical disorder; Cephalosporin allergy; Severe psychiatric disorders	1) G 1 (n = 28)<br>Attrition = 29%<br>Age: 48.0 ± 11.8<br>Sex: 46.4% M<br>DOI: NR<br><br>G 2 (n = 24)<br>Attrition = 29%<br>Age: 47.0 ± 9.7<br>Sex: 48.2% M<br>DOI: NR<br><br>2) Age, sex, %white, edu, EMPL, LD characteristics, ELISA+Western Blot (+), prior AB treatment, fatigue+cog+other clinical measures	1) AAT<br><br>2) Cog processing (mental) speed	ITT analysis<br><br>χ^2<br><br>Fisher exact tests<br><br>Wilcoxon rank sum tests	G1<br>*BL; 6 mo (mean ± SD)*<br>Mental speed<br>4.1 ± 1.5; 3.8 ± 1.7<br><br>G2<br>*BL; 6 mo (mean ± SD)*<br>Mental speed<br>3.8 ± 1.3; 3.4 ± 1.0<br><br>*Change*<br>G1<br>−0.3 ± 1.0<br>G2<br>−0.5 ± 0.8	*p*<br>Mental speed<br>NS<br>Change in mental speed<br>NS<br><br>6 mo FU: no SGNFT ↑ cog function or group differences; AB treatment ≠ ↑ cog performance in pts w/ LD	1) 88%<br><br>2)<br>G1: Diarrhea, anaphylaxis, minor allergic reaction<br>G2: IV sepsis
1) Otto M, Cepek L, Ratzka P, et al. (2004) [34]<br>2) German National CJD	1) CJD<br><br>2) To test if FLU ↓ cog deficits related to AD in Pts w/ CJD.<br><br>3) G1: FLU (100 mg)	1) Double-blind PLA-RCT<br><br>2) ≥50% score in 2/12 subtests of dementia tests	1) G 1 (n = 13)<br>Attrition = 7.7%<br>Age: 57.0 ± 9.6<br>Sex: 61.5% M<br>DOI: NR<br><br>G 2 (n = 15)	1) ADAS<br>MMSE<br>GoeCJDDT<br><br>2) long-term & short-term memory	One-sided t-test<br><br>ITT analysis	G1<br>*BL; 6 mo (mean ± SD)*<br>ADAS-Cog<br>28.2 ± 9.4; NR<br>MMSE<br>19.2 ± 3.5; NR	*p*<br>ADAS-Cog<br>0.02<br>MMSE<br>0.07	1) NR<br><br>2)<br>G1: muscle weakness<br>G2: lack of tolerability

Surveillance Unit, Goettingen<br>3) Germany<br>4) BMG & BMBF	G2: PLA<br><br>4) Drug (FLU)<br><br>5) 5.5 wks<br><br>6) NR	3) NR	Attrition = 13%<br>Age: 61.0 ± 10.3<br>Sex: 50.0% M<br>DOI: NR<br><br>2) Age, sex, clinical signs, CSF parameter, codon 129 polymorphism, genetic CJD, MMSE, ADAS-Cog	Attention<br>Executive functions<br>Language<br>Spatial processing		G2<br>*BL; 6 mo (mean ± SD)*<br>ADAS-Cog<br>25.1 ± 10.1; NR<br>MMSE<br>20.5 ± 4.7; NR<br><br>*Change*<br>G1<br>ADAS-Cog<br>8.4 ± 15.3<br>MMSE<br>−3.3 ± 7.7<br><br>G2<br>ADAS-Cog<br>20.6 ± 15.1<br>MMSE<br>−8.0 ± 7.6	G1 had SGNFT in ↓ cog deficits than G2; FLU has SGNFT effects on cog in pts w/ CJD	Unspecified group: gastrointestinal bleeding, urticaria

Abbreviations: M = male(s); BL = baseline; FU = follow-up; t1 = Time 1; t2 = Time 2; Inst. = Institute; SMRI = Stanley Medical Research Institute; NIMH = National Institute of Mental Health; HSV-1 = herpes simplex virus 1; VAV = Valacyclovir; AP = Antipsychotics; cog = cognitive/cognition; Scz = Schizophrenia; Pts = patients; G = group; PLA = placebo; Wks = weeks; NR = not reported; RCT = randomized controlled trial; Ab = antibody/antibodies; mo = months; PANSS = positive & negative syndrome scale; DSM-IV = Diagnostic and Statistical Manual of Mental Disorders, fourth edition; SES = socioeconomic status; DOI = duration of illness; PennCNB = penn computerized neurocognitive battery; ITT = intention to treat; Obj = object; NS = not significant; NNOSR = Netherlands National Organization for Scientific Research; LD = lyme disease; LT = long-term; AB = antibiotics; CTX = ceftriaxone; DOX = doxycycline; CLR-HCQ = clarithromycin-hydroxychloroquine; ST = short-term; F = female(s); MSK = musculoskeletal; EM = erythema migrans; TCN = tetracycline; d = day/days; IV = intravenous; AMT = antimicrobial therapy; CFS = chronic fatigue syndrome; Sev = severe; SX = symptoms; Edu = education; EMPL = employment; RAVLT = Rey; Auditory Verbal learning test; DST = Digit Span Test; TMT-A = Trail Making Test Part A; SCWT = Stroop Color-Word Test; DSST = Symbol-Digit Substitution Test; CFT = Category Fluency Test; SGFNT = significant; TX = Treatment; DSM IV-TR = Diagnostic and Statistical Manual of Mental Disorders Text Revision Fourth Edition; CGI-S = Clinical Global Impression Severity scale; MATRICS = Measurement and Treatment Research to Improve Cognition in Psychosis; MCCB = MATRICS Consensus Cognitive Battery; LDANJ = Lyme Disease Association of New Jersey; NPIRSG = NYS Psychiatric Institute Research Support Grant; IM = Intramuscular; WAIS = Wechsler Adult Intelligence Scale; WMS = Wechsler Memory Scale; COWAT = Controlled Oral Word Association Test; DOT = duration of time; NINDS = National Institute of Neurological Disorders and Stroke; BVRT = Benton Visual Retention Test; BSRT = Buschke Selective Reminding Test; LRT = Logical Reasoning Test; CPT = Continuous Performance Test; SRT = Simple Reaction Time; CRT = Choice Reaction Time; TMT-B = Trail Making Test Part B; DSTGI = Department of Science and Technology, Government of India; NIH = National Institutes of Health; HOH = head of household; EMOD = Emotion Identification & Discrimination; NIAID = National Institute of Allergy and Infectious Diseases; HHS = U.S. Department of Health and Human Services; CSF = cerebrospinal fluid; SDMT = Symbol Digit Modalities Test; CalCAP = California Computerized Assessment Package; NCRR = National Center for Research Resources; AAT = Alpha-arithmetic Test; BMG = German Federal Ministry of Health; BMBF = Federal Ministry of Science and Technology; FLU = Flupirtine maleate; AD = Alzheimer Disease; ADAS = Alzheimer's Disease Assessment Scale; MMSE = Mini-Mental State Examination; GoeCJDDT = Goettingen CJD Dementia Test

* = Median stated
