# Supplementary material for: Treating cognitive impairments in primary central nervous system infections: A systematic review of pharmacological interventions
Source: Medicine (Baltimore). 2023 Jul 14;102(28):e34151. doi: 10.1097/MD.0000000000034151 (PMC10344564; doi:10.1097/MD.0000000000034151)
Supplement: Supplementary file 3 [file medi-102-e34151-s003.pdf]

**Table S3. Articles excluded after full-text review and their corresponding reasons for exclusion**

| Citation | Reasons for exclusion |
|---|---|
| [26]. Breier A, Buchanan RW, D'Souza D, et al. Herpes simplex virus 1 infection and valacyclovir treatment in schizophrenia: Results from the VISTA study. *Schizophr Res*. 2019;206:291-299. doi:10.1016/j.schres.2018.11.002 | Repeated study |
| [27]. Carlson RD, Rolfes MA, Birkenkamp KE, et al. Predictors of neurocognitive outcomes on antiretroviral therapy after cryptococcal meningitis: a prospective cohort study. *Metab Brain Dis*. 2014;29(2):269-279. doi:10.1007/s11011-013-9476-1 | Did not focus on primary CNS infection |
| [28]. Chen CH, Chang CC, Chang WN, et al. Neuro-psychological sequelae in HIV-negative cryptococcal meningitis after complete anti-fungal treatment. *Acta Neurol Taiwan*. 2012;21(1):8-17. | Not a pre-post intervention, randomized controlled trial or nonrandomized study |
| [30]. Gnann JW Jr, Agrawal A, Hart J, et al. Lack of Efficacy of High-Titered Immunoglobulin in Patients with West Nile Virus Central Nervous System Disease. *Emerg Infect Dis*. 2019;25(11):2064-2073. doi:10.3201/eid2511.190537 | No longitudinal assessment of cognition |
| [33]. Tzeng NS, Chung CH, Lin FH, et al. Anti-herpetic Medications and Reduced Risk of Dementia in Patients with Herpes Simplex Virus Infections-a Nationwide, Population-Based Cohort Study in Taiwan. *Neurotherapeutics*. 2018;15(2):417-429. doi:10.1007/s13311-018-0611-x | Not a pre-post intervention, randomized controlled trial or nonrandomized study |
| [35]. Wahlund LO, Lindqvist L, Astrom M, et al. Enterovirus might be involved in Alzheimer's disease-results from a phase iia trial evaluating apovir, an antiviral drug combination. *Journal of prevention of alzheimer's disease*. 2018;5(1): S165-S166. doi:10.14283/jpad.2018.40 | Full study not found, no response from author |
| [37]. Weisfelt M, Hoogman M, van de Beek D, de Gans J, Dreschler WA, Schmand BA. Dexamethasone and long-term outcome in adults with bacterial meningitis. *Ann Neurol*. 2006;60(4):456-468. doi:10.1002/ana.20944 | Not an interventional study |
| [38]. Westman G, Studahl M, Ahlm C, et al. N-methyl-d-aspartate receptor autoimmunity affects cognitive performance in herpes simplex encephalitis. *Clin Microbiol Infect*. 2016;22(11):934-940. doi:10.1016/j.cmi.2016.07.028 | Did not meet inclusion criteria |
| [39]. Nimgaonkar VL, Bhatia T, Wood J, Gur R, Deshpande S. Dysfunctional emotion discrimination in Schizophrenia is associated with hsv-1 infection and improves with antiviral treatment. *Biological Psychiatry*. 2017;81(10):S163-. https://doi.org/10.1016/j.biopsych.2017.02.417 | Did not meet inclusion criteria |
| [40]. Prasad K, et al. Treatment of neurotropic infectious agents to alleviate cognitive deficits in schizophrenia: A test of concept randomized double blind placebo controlled trial. *Neuropsychopharmacology*. 2010;35(5). | MS is the same as another included study |
