# Supplementary material for: Treating cognitive impairments in primary central nervous system infections: A systematic review of pharmacological interventions
Source: Medicine (Baltimore). 2023 Jul 14;102(28):e34151. doi: 10.1097/MD.0000000000034151 (PMC10344564; doi:10.1097/MD.0000000000034151)
Supplement: Supplementary file 4 [file medi-102-e34151-s004.pdf]

Table S4: Summarized data of changes in different cognitive domains across studies.

Author (Year)	Infectious agent	Complex attention	Executive functioning	Learning and memory	Language	Perceptual-motor ability	Social cognition
Prasad KM, Eack SM, Keshavan MS, Yolken RH, Iyengar S, Nimgaonkar VL. (2013) [36]	HSV-1		+		+		
Breier A, Dickerson F, Buchanan R, et al. (2018) [25]	HSV-1		=	=	=	=	
Bhatia T, Wood J, Iyengar S, et al. (2018) [24]	HSV-1	=	=	=		=	+
Otto M, Cepek L, Ratzka P, et al. (2004) [34]	CJD	+	+	+	+	+	
Berende A, Ter Hofstede HJM, Vos FJ, et al. (2019) [23]	LD	+	=	=	+		
Fallon BA, Tager F, Fein L, Liegner K, Keilp J, Weiss N, Liebowitz MR. (1999) [17]	LD	=		+	+		
Fallon BA, Keilp JG, Corbera KM, et al. (2008) [29]	LD	+	+	+	+	+	
Kaplan RF, Trevino RP, Johnson GM, et al. (2003) [31]	LD	+		+	+		
Krupp LB, Hyman LG, Grimson R, et al. (2003) [32]	LD	=			=		

Abbreviations: HSV-1 = herpes simplex virus; CJD = Creutzfeldt-Jakob disease; LD = Lyme disease. * Reported from baseline to the last follow-up

Legend:

=	No significant change

+	Improvement
	Not tested
