# Supplementary material for: Treating cognitive impairments in primary central nervous system infections: A systematic review of pharmacological interventions
Source: Medicine (Baltimore). 2023 Jul 14;102(28):e34151. doi: 10.1097/MD.0000000000034151 (PMC10344564; doi:10.1097/MD.0000000000034151)
Supplement: Supplementary file 5 [file medi-102-e34151-s005.pdf]

Table S5. Risk of bias study quality assessment using the Risk Of Bias in Non-randomised Studies of Interventions (ROBINS-I) tool, version of 2016 and the risk of bias for RCT studies using version 2 of the Cochrane risk-of-bias tool for randomized trials (RoB 2).

Risk of bias summary (ROBINS 1): review authors' judgements about each risk of bias item for each included NRS study. The overall quality (certainty) of the evidence was assessed as high, moderate, low, or very low as per Guyatt et al., 2011*.

Study	D1	D2	D3	D4	D5	D6	D7	Overall	Certainty assessment*
Fallon et al. (1999) *	-	-	+	X	+	-	-	X	Very low

Domains:
D1: Bias arising from bias due to confounding
D2: Bias due to selection of participants into the study
D3: Bias due to classification of interventions
D4: Bias due to deviations from the intended intervention
D5: Bias due to missing data
D6: Bias in measurement of outcomes
D7: Bias in selection of the reported results

Judgement:
X High
- Some concern
+ Low

Figure. Risk of bias summary (RoB 2): review authors' judgements about each risk of bias item for each included study. The overall quality (certainty) of the evidence was assessed as high, moderate, low, or very low as per Guyatt et al., 2011*.

Study	D1	D2	D3	D4	D5	Overall	Certainty assessment*
Berende, et al. (2019)	-	-	-	+	+	-	Low
Bhatia et al. (2018)	+	-	+	+	+	-	Low
Breier et al. (2018)	-	-	X	+	-	X	Very low

Fallon et al. (2008)	+	-	+	+	+	+	Moderate
Kaplan et al. (2003)	-	-	-	X	-	X	Very low
Krupp et al. (2003)	-	-	+	+	-	-	Low
Prasad et al. (2013)	-	-	-	-	+	-	Low
Otto et al. (2004)	X	-	+	+	-	X	Very low

Domains:
D1: Bias arising from the randomization process
D2: Bias due to deviations from intended intervention
D3: Bias due to missing outcome data
D4: Bias in measurement of the outcome
D5: Bias in selection of the reporting results

Judgement:
X High
- Some concern
+ Low
